# Supplementary material for: Heterogeneity induced GZMA-F2R communication inefficient impairs antitumor immunotherapy of PD-1 mAb through JAK2/STAT1 signal suppression in hepatocellular carcinoma
Source: Cell Death Dis. 2022 Mar 7;13(3):213. doi: 10.1038/s41419-022-04654-7 (PMC8901912; doi:10.1038/s41419-022-04654-7)
Supplement: Supplementary file 1 — Table S1 [file 41419_2022_4654_MOESM1_ESM.docx]

| Table S1: Marker gene in subpopulation of HCC patients | | | | | | |
| --- | --- | --- | --- | --- | --- | --- |
| Genes | P_val | Avg_logfc | Pct.1 | Pct.2 | P_val_adj | Cluster |
| KLRB1 | 0 | 1.757993 | 0.71 | 0.227 | 0 | 0 |
| CXCR4 | 0 | 1.086825 | 0.981 | 0.721 | 0 | 0 |
| KLRD1 | 0 | 1.079673 | 0.534 | 0.146 | 0 | 0 |
| IL7R | 0 | 1.062422 | 0.49 | 0.21 | 0 | 0 |
| CD8A | 0 | 0.942221 | 0.323 | 0.069 | 0 | 0 |
| CD3D | 0 | 0.87426 | 0.7 | 0.283 | 0 | 0 |
| PTPRC | 0 | 0.731916 | 0.801 | 0.506 | 0 | 0 |
| CD3G | 0 | 0.637292 | 0.382 | 0.143 | 0 | 0 |
| ID2 | 2.64E-300 | 0.456957 | 0.729 | 0.671 | 5.96E-296 | 0 |
| CD37 | 1.07E-286 | 0.358214 | 0.527 | 0.39 | 2.41E-282 | 0 |
| ANXA1 | 1.05E-266 | 0.384239 | 0.674 | 0.573 | 2.38E-262 | 0 |
| EZR | 1.57E-261 | 0.399725 | 0.556 | 0.461 | 3.55E-257 | 0 |
| AAK1 | 6.85E-207 | 0.468867 | 0.332 | 0.232 | 1.54E-202 | 0 |
| CD44 | 2.68E-180 | 0.347286 | 0.549 | 0.47 | 6.05E-176 | 0 |
| PBXIP1 | 3.69E-167 | 0.400793 | 0.257 | 0.161 | 8.33E-163 | 0 |
| FABP5 | 0 | 1.686402 | 0.652 | 0.326 | 0 | 1 |
| BCL2A1 | 0 | 1.646404 | 0.792 | 0.229 | 0 | 1 |
| LYZ | 0 | 1.523703 | 0.929 | 0.319 | 0 | 1 |
| AIF1 | 0 | 1.413284 | 0.957 | 0.296 | 0 | 1 |
| CD68 | 0 | 1.377852 | 0.858 | 0.237 | 0 | 1 |
| LGALS3 | 0 | 1.210465 | 0.747 | 0.277 | 0 | 1 |
| CD14 | 0 | 1.129981 | 0.618 | 0.207 | 0 | 1 |
| ANXA5 | 0 | 0.892043 | 0.832 | 0.407 | 0 | 1 |
| FPR1 | 0 | 0.877476 | 0.424 | 0.069 | 0 | 1 |
| GLUL | 0 | 0.86945 | 0.866 | 0.494 | 0 | 1 |
| CD9 | 0 | 0.789044 | 0.538 | 0.221 | 0 | 1 |
| FCGR3A | 0 | 0.681483 | 0.635 | 0.242 | 0 | 1 |
| ABCA1 | 0 | 0.495537 | 0.327 | 0.097 | 0 | 1 |
| ITGAX | 0 | 0.49271 | 0.302 | 0.057 | 0 | 1 |
| CCL3 | 1.33E-289 | 1.19975 | 0.742 | 0.481 | 3.01E-285 | 1 |
| CD74 | 3.20E-279 | 0.299669 | 0.982 | 0.893 | 7.21E-275 | 1 |
| CD163 | 4.78E-279 | 0.321694 | 0.46 | 0.168 | 1.08E-274 | 1 |
| ACTB | 8.72E-221 | 0.385942 | 0.994 | 0.991 | 1.97E-216 | 1 |
| CD83 | 2.09E-203 | 0.364308 | 0.575 | 0.284 | 4.72E-199 | 1 |
| ICAM1 | 3.91E-185 | 0.40111 | 0.46 | 0.218 | 8.82E-181 | 1 |
| CD441 | 8.16E-154 | 0.408696 | 0.735 | 0.478 | 1.84E-149 | 1 |
| ANXA11 | 1.01E-132 | 0.250997 | 0.835 | 0.589 | 2.29E-128 | 1 |
| TFRC | 2.63E-128 | 0.333025 | 0.265 | 0.112 | 5.93E-124 | 1 |
| RTN4 | 7.86E-78 | 0.272809 | 0.621 | 0.433 | 1.77E-73 | 1 |
| CTNNB1 | 8.68E-73 | 0.387867 | 0.497 | 0.328 | 1.96E-68 | 1 |
| CD741 | 0 | 1.680043 | 1 | 0.893 | 0 | 2 |
| LYZ1 | 0 | 1.663363 | 0.979 | 0.323 | 0 | 2 |
| CD1C | 0 | 1.02147 | 0.354 | 0.013 | 0 | 2 |
| AIF11 | 0 | 0.916374 | 0.966 | 0.304 | 0 | 2 |
| CPM | 0 | 0.843488 | 0.515 | 0.139 | 0 | 2 |
| CD831 | 0 | 0.667586 | 0.701 | 0.28 | 0 | 2 |
| CD681 | 0 | 0.62051 | 0.713 | 0.254 | 0 | 2 |
| AXL | 0 | 0.524043 | 0.44 | 0.111 | 0 | 2 |
| CD1631 | 0 | 0.504835 | 0.517 | 0.168 | 0 | 2 |
| ITGAX1 | 0 | 0.449295 | 0.348 | 0.057 | 0 | 2 |
| ADAM28 | 0 | 0.301709 | 0.275 | 0.035 | 0 | 2 |
| LGALS31 | 1.24E-256 | 0.58931 | 0.643 | 0.29 | 2.80E-252 | 2 |
| BCL2A11 | 1.77E-256 | 0.416148 | 0.601 | 0.248 | 3.98E-252 | 2 |
| ANXA51 | 1.05E-242 | 0.380807 | 0.822 | 0.413 | 2.36E-238 | 2 |
| FPR11 | 4.14E-214 | 0.294705 | 0.28 | 0.082 | 9.34E-210 | 2 |
| ACTB1 | 7.10E-190 | 0.425879 | 0.999 | 0.99 | 1.60E-185 | 2 |
| FABP51 | 1.73E-158 | 0.420553 | 0.634 | 0.331 | 3.90E-154 | 2 |
| RTN41 | 1.21E-137 | 0.2906 | 0.744 | 0.428 | 2.73E-133 | 2 |
| CD141 | 2.46E-116 | 0.316112 | 0.438 | 0.223 | 5.54E-112 | 2 |
| CCL31 | 6.45E-57 | 0.447024 | 0.672 | 0.488 | 1.46E-52 | 2 |
| KLRD11 | 0 | 1.278838 | 0.808 | 0.241 | 0 | 3 |
| FCGR3A1 | 0 | 1.047972 | 0.619 | 0.248 | 0 | 3 |
| B2M | 0 | 0.33232 | 1 | 0.999 | 0 | 3 |
| CXCR41 | 1.47E-218 | 0.466818 | 0.958 | 0.798 | 3.31E-214 | 3 |
| ANXA12 | 4.00E-200 | 0.591247 | 0.804 | 0.594 | 9.02E-196 | 3 |
| PTPRC1 | 3.02E-165 | 0.481706 | 0.781 | 0.592 | 6.81E-161 | 3 |
| KLRB11 | 1.08E-124 | 0.31594 | 0.633 | 0.371 | 2.45E-120 | 3 |
| CD99 | 2.48E-123 | 0.512398 | 0.655 | 0.542 | 5.59E-119 | 3 |
| SPN | 1.14E-114 | 0.530004 | 0.3 | 0.142 | 2.56E-110 | 3 |
| CD3G1 | 1.79E-97 | 0.561932 | 0.377 | 0.212 | 4.05E-93 | 3 |
| ID21 | 7.29E-91 | 0.399784 | 0.767 | 0.685 | 1.64E-86 | 3 |
| CD371 | 2.24E-82 | 0.367335 | 0.576 | 0.426 | 5.06E-78 | 3 |
| CD3D1 | 6.55E-75 | 0.437653 | 0.568 | 0.411 | 1.48E-70 | 3 |
| CD8A1 | 1.75E-73 | 0.407597 | 0.28 | 0.145 | 3.94E-69 | 3 |
| AAK11 | 9.88E-24 | 0.305859 | 0.322 | 0.261 | 2.23E-19 | 3 |
| ITGB1 | 1.04E-14 | 0.286167 | 0.431 | 0.42 | 2.35E-10 | 3 |
| CD1632 | 0 | 2.045923 | 0.928 | 0.151 | 0 | 4 |
| VCAM1 | 0 | 1.90103 | 0.915 | 0.127 | 0 | 4 |
| CCL32 | 0 | 1.802558 | 0.952 | 0.476 | 0 | 4 |
| FCGR3A2 | 0 | 1.640277 | 0.929 | 0.236 | 0 | 4 |
| AIF12 | 0 | 1.513051 | 0.988 | 0.31 | 0 | 4 |
| AXL1 | 0 | 1.407648 | 0.802 | 0.096 | 0 | 4 |
| CD682 | 0 | 1.233148 | 0.903 | 0.249 | 0 | 4 |
| GLUL1 | 0 | 0.910976 | 0.97 | 0.497 | 0 | 4 |
| CD742 | 0 | 0.847635 | 0.999 | 0.895 | 0 | 4 |
| CD832 | 0 | 0.754839 | 0.678 | 0.286 | 0 | 4 |
| CD4 | 0 | 0.718716 | 0.654 | 0.148 | 0 | 4 |
| ABCA11 | 0 | 0.689589 | 0.538 | 0.091 | 0 | 4 |
| ICAM11 | 0 | 0.684552 | 0.61 | 0.216 | 0 | 4 |
| CD142 | 0 | 0.677736 | 0.663 | 0.214 | 0 | 4 |
| CPM1 | 0 | 0.639784 | 0.592 | 0.139 | 0 | 4 |
| FPR12 | 0 | 0.604175 | 0.502 | 0.073 | 0 | 4 |
| ANXA52 | 0 | 0.564247 | 0.838 | 0.417 | 0 | 4 |
| ACTN1 | 0 | 0.543146 | 0.501 | 0.129 | 0 | 4 |
| SLC8A1 | 0 | 0.522891 | 0.397 | 0.044 | 0 | 4 |
| ALDH1A1 | 0 | 0.419053 | 0.652 | 0.204 | 0 | 4 |
| B2M1 | 0 | 0.399311 | 1 | 0.999 | 0 | 4 |
| ACVRL1 | 0 | 0.388671 | 0.309 | 0.031 | 0 | 4 |
| CDH5 | 0 | 0.370551 | 0.313 | 0.038 | 0 | 4 |
| CD38 | 0 | 0.315348 | 0.387 | 0.091 | 0 | 4 |
| ABRACL | 3.01E-243 | 0.489201 | 0.764 | 0.387 | 6.78E-239 | 4 |
| NRP1 | 5.29E-209 | 0.313056 | 0.341 | 0.106 | 1.19E-204 | 4 |
| RTN42 | 1.85E-171 | 0.403693 | 0.764 | 0.43 | 4.18E-167 | 4 |
| KLF6 | 1.39E-164 | 0.508531 | 0.906 | 0.657 | 3.14E-160 | 4 |
| CD442 | 6.54E-123 | 0.2973 | 0.805 | 0.48 | 1.47E-118 | 4 |
| TNFRSF18 | 0 | 1.216626 | 0.301 | 0.048 | 0 | 5 |
| CD27 | 0 | 1.072196 | 0.403 | 0.092 | 0 | 5 |
| CD3D2 | 0 | 1.002946 | 0.814 | 0.402 | 0 | 5 |
| B2M2 | 0 | 0.485859 | 1 | 0.999 | 0 | 5 |
| PTPRC2 | 1.03E-136 | 0.53795 | 0.764 | 0.595 | 2.33E-132 | 5 |
| CD3G2 | 3.57E-89 | 0.491272 | 0.396 | 0.213 | 8.05E-85 | 5 |
| ANXA13 | 3.25E-22 | 0.333892 | 0.399 | 0.617 | 7.33E-18 | 5 |
| PBXIP11 | 5.37E-18 | 0.344169 | 0.251 | 0.19 | 1.21E-13 | 5 |
| ANP32E | 3.69E-15 | 0.351353 | 0.264 | 0.216 | 8.33E-11 | 5 |
| TM4SF1 | 0 | 2.65078 | 0.937 | 0.162 | 0 | 6 |
| AKAP12 | 0 | 2.156277 | 0.57 | 0.06 | 0 | 6 |
| A2M | 0 | 1.542858 | 0.818 | 0.22 | 0 | 6 |
| ENG | 0 | 1.503453 | 0.688 | 0.077 | 0 | 6 |
| CD91 | 0 | 1.494025 | 0.728 | 0.221 | 0 | 6 |
| ADAMTS1 | 0 | 1.476356 | 0.498 | 0.035 | 0 | 6 |
| TSPAN7 | 0 | 1.443464 | 0.607 | 0.027 | 0 | 6 |
| PECAM1 | 0 | 1.417509 | 0.692 | 0.112 | 0 | 6 |
| FLT1 | 0 | 1.366982 | 0.648 | 0.036 | 0 | 6 |
| IL6ST | 0 | 1.312293 | 0.741 | 0.178 | 0 | 6 |
| ADAMTS4 | 0 | 1.255488 | 0.422 | 0.03 | 0 | 6 |
| MYC | 0 | 1.120694 | 0.489 | 0.146 | 0 | 6 |
| VWF | 0 | 1.075306 | 0.351 | 0.017 | 0 | 6 |
| CDH51 | 0 | 1.020542 | 0.549 | 0.028 | 0 | 6 |
| ADAM15 | 0 | 0.858358 | 0.404 | 0.056 | 0 | 6 |
| CD34 | 0 | 0.836059 | 0.392 | 0.015 | 0 | 6 |
| LRRC32 | 0 | 0.740369 | 0.341 | 0.024 | 0 | 6 |
| NRP11 | 0 | 0.714209 | 0.426 | 0.103 | 0 | 6 |
| ITGA5 | 0 | 0.574128 | 0.366 | 0.065 | 0 | 6 |
| ITGA6 | 0 | 0.508565 | 0.266 | 0.037 | 0 | 6 |
| IL3RA | 0 | 0.498855 | 0.276 | 0.023 | 0 | 6 |
| ITGB11 | 1.79E-288 | 0.676135 | 0.774 | 0.405 | 4.03E-284 | 6 |
| CTNNB11 | 1.57E-154 | 0.641657 | 0.608 | 0.328 | 3.54E-150 | 6 |
| ICAM12 | 3.65E-143 | 0.692181 | 0.472 | 0.224 | 8.24E-139 | 6 |
| CD40 | 5.10E-123 | 0.306448 | 0.266 | 0.095 | 1.15E-118 | 6 |
| CD46 | 1.02E-120 | 0.358506 | 0.427 | 0.198 | 2.31E-116 | 6 |
| CD41 | 2.32E-92 | 0.69437 | 0.33 | 0.166 | 5.24E-88 | 6 |
| ACTN11 | 1.81E-84 | 0.315053 | 0.301 | 0.14 | 4.09E-80 | 6 |
| RTN43 | 1.67E-66 | 0.309832 | 0.652 | 0.437 | 3.77E-62 | 6 |
| CD991 | 7.31E-64 | 0.270797 | 0.743 | 0.539 | 1.65E-59 | 6 |
| KLF61 | 8.07E-56 | 0.286711 | 0.829 | 0.662 | 1.82E-51 | 6 |
| CD143 | 3.95E-49 | 0.938292 | 0.356 | 0.23 | 8.90E-45 | 6 |
| APOE | 0 | 2.950242 | 0.991 | 0.572 | 0 | 7 |
| TF | 0 | 2.313183 | 0.984 | 0.226 | 0 | 7 |
| A1BG | 0 | 2.071976 | 0.97 | 0.21 | 0 | 7 |
| AADAC | 0 | 1.505479 | 0.882 | 0.058 | 0 | 7 |
| SLC2A2 | 0 | 1.407996 | 0.854 | 0.067 | 0 | 7 |
| ADI1 | 0 | 1.378785 | 0.964 | 0.289 | 0 | 7 |
| KRT18 | 0 | 1.356317 | 0.951 | 0.189 | 0 | 7 |
| CYP17A1 | 0 | 1.226 | 0.779 | 0.052 | 0 | 7 |
| SDC1 | 0 | 0.714137 | 0.622 | 0.059 | 0 | 7 |
| CD24 | 0 | 0.698819 | 0.625 | 0.082 | 0 | 7 |
| CADM1 | 0 | 0.638332 | 0.565 | 0.051 | 0 | 7 |
| ACAT2 | 0 | 0.553429 | 0.6 | 0.116 | 0 | 7 |
| ALDH1A11 | 0 | 0.446429 | 0.774 | 0.203 | 0 | 7 |
| ACADS | 0 | 0.437323 | 0.479 | 0.065 | 0 | 7 |
| ACADSB | 0 | 0.409052 | 0.446 | 0.051 | 0 | 7 |
| CDH2 | 0 | 0.360973 | 0.353 | 0.02 | 0 | 7 |
| ALDH1L1 | 0 | 0.337208 | 0.42 | 0.055 | 0 | 7 |
| ANPEP | 0 | 0.255237 | 0.366 | 0.071 | 0 | 7 |
| IL7R1 | 0 | 1.403577 | 0.938 | 0.28 | 0 | 8 |
| CCR6 | 0 | 0.986529 | 0.507 | 0.084 | 0 | 8 |
| CCR7 | 3.73E-286 | 0.888496 | 0.383 | 0.091 | 8.41E-282 | 8 |
| ANXA14 | 3.14E-280 | 0.92965 | 0.884 | 0.597 | 7.08E-276 | 8 |
| KLF62 | 3.51E-253 | 0.807247 | 0.939 | 0.659 | 7.91E-249 | 8 |
| CD3D3 | 3.48E-194 | 0.477446 | 0.848 | 0.405 | 7.86E-190 | 8 |
| CXCR42 | 3.47E-192 | 0.533821 | 0.996 | 0.8 | 7.82E-188 | 8 |
| CD3G3 | 6.43E-192 | 0.541221 | 0.565 | 0.209 | 1.45E-187 | 8 |
| CD443 | 4.86E-183 | 0.572362 | 0.829 | 0.484 | 1.10E-178 | 8 |
| PBXIP12 | 2.26E-174 | 0.573696 | 0.479 | 0.182 | 5.11E-170 | 8 |
| PTPRC3 | 7.90E-150 | 0.478177 | 0.89 | 0.593 | 1.78E-145 | 8 |
| CD6 | 8.72E-102 | 0.371063 | 0.285 | 0.1 | 1.97E-97 | 8 |
| AAK12 | 1.02E-94 | 0.454245 | 0.488 | 0.257 | 2.30E-90 | 8 |
| CD271 | 2.43E-85 | 0.413885 | 0.266 | 0.101 | 5.49E-81 | 8 |
| EZR1 | 4.18E-82 | 0.398456 | 0.702 | 0.485 | 9.43E-78 | 8 |
| ABRACL1 | 5.96E-45 | 0.324539 | 0.553 | 0.4 | 1.34E-40 | 8 |
| ITGB12 | 2.40E-39 | 0.277128 | 0.563 | 0.416 | 5.40E-35 | 8 |
| ALDH1A12 | 0 | 1.835139 | 0.933 | 0.207 | 0 | 9 |
| GLUL2 | 0 | 1.834991 | 0.946 | 0.509 | 0 | 9 |
| ALDH1L11 | 0 | 1.549591 | 0.857 | 0.049 | 0 | 9 |
| SLC2A21 | 0 | 1.384879 | 0.856 | 0.079 | 0 | 9 |
| KRT181 | 0 | 1.251012 | 0.932 | 0.201 | 0 | 9 |
| TF1 | 0 | 1.208627 | 0.94 | 0.239 | 0 | 9 |
| ADI11 | 0 | 1.079448 | 0.923 | 0.3 | 0 | 9 |
| APOE1 | 0 | 1.045461 | 0.965 | 0.579 | 0 | 9 |
| SDC11 | 0 | 0.715578 | 0.603 | 0.068 | 0 | 9 |
| CYP17A11 | 0 | 0.67313 | 0.537 | 0.069 | 0 | 9 |
| CYP2C9 | 0 | 0.637173 | 0.527 | 0.053 | 0 | 9 |
| ACAT21 | 0 | 0.530087 | 0.569 | 0.124 | 0 | 9 |
| ABCG2 | 0 | 0.469481 | 0.404 | 0.028 | 0 | 9 |
| TM4SF11 | 0 | 0.384861 | 0.784 | 0.181 | 0 | 9 |
| AOX1 | 0 | 0.352569 | 0.326 | 0.032 | 0 | 9 |
| CD241 | 2.87E-274 | 0.310309 | 0.455 | 0.094 | 6.47E-270 | 9 |
| AADAC1 | 1.80E-229 | 0.278424 | 0.396 | 0.083 | 4.05E-225 | 9 |
| ACTL6A | 3.04E-155 | 0.257549 | 0.347 | 0.091 | 6.85E-151 | 9 |
| CCL33 | 0 | 1.65885 | 0.958 | 0.488 | 0 | 10 |
| CD743 | 0 | 1.581131 | 1 | 0.897 | 0 | 10 |
| APOE2 | 0 | 1.496174 | 1 | 0.579 | 0 | 10 |
| CD683 | 0 | 1.450808 | 0.964 | 0.264 | 0 | 10 |
| AIF13 | 0 | 1.446626 | 0.992 | 0.326 | 0 | 10 |
| CD144 | 0 | 1.111228 | 0.802 | 0.221 | 0 | 10 |
| LGALS32 | 0 | 1.052588 | 0.86 | 0.297 | 0 | 10 |
| CD833 | 0 | 1.003541 | 0.859 | 0.291 | 0 | 10 |
| CD92 | 0 | 0.994963 | 0.787 | 0.23 | 0 | 10 |
| A2M1 | 0 | 0.858725 | 0.784 | 0.233 | 0 | 10 |
| CD401 | 0 | 0.733913 | 0.472 | 0.093 | 0 | 10 |
| TF2 | 0 | 0.409736 | 0.843 | 0.241 | 0 | 10 |
| ADORA3 | 9.92E-299 | 0.408375 | 0.259 | 0.033 | 2.24E-294 | 10 |
| ICAM13 | 4.26E-297 | 0.813573 | 0.705 | 0.223 | 9.61E-293 | 10 |
| GLUL3 | 7.54E-266 | 0.792495 | 0.949 | 0.509 | 1.70E-261 | 10 |
| AXL2 | 1.06E-264 | 0.58913 | 0.503 | 0.121 | 2.40E-260 | 10 |
| FCGR3A3 | 5.95E-236 | 0.525003 | 0.769 | 0.257 | 1.34E-231 | 10 |
| ABCA12 | 2.78E-234 | 0.562054 | 0.427 | 0.105 | 6.28E-230 | 10 |
| A1BG1 | 2.52E-219 | 0.382969 | 0.686 | 0.228 | 5.69E-215 | 10 |
| LYZ2 | 5.10E-191 | 0.265633 | 0.814 | 0.35 | 1.15E-186 | 10 |
| CD42 | 1.76E-153 | 0.425079 | 0.492 | 0.165 | 3.98E-149 | 10 |
| BCL2A12 | 2.44E-147 | 0.550634 | 0.638 | 0.26 | 5.51E-143 | 10 |
| CPM2 | 5.21E-133 | 0.380048 | 0.449 | 0.154 | 1.17E-128 | 10 |
| RTN44 | 9.36E-117 | 0.469062 | 0.801 | 0.438 | 2.11E-112 | 10 |
| ANXA53 | 2.32E-107 | 0.432507 | 0.792 | 0.428 | 5.24E-103 | 10 |
| ITGAX2 | 2.37E-106 | 0.295398 | 0.254 | 0.07 | 5.34E-102 | 10 |
| NRP12 | 2.04E-101 | 0.352536 | 0.328 | 0.112 | 4.60E-97 | 10 |
| ACTB2 | 7.06E-97 | 0.357972 | 1 | 0.991 | 1.59E-92 | 10 |
| TFRC1 | 6.64E-90 | 0.365976 | 0.329 | 0.118 | 1.50E-85 | 10 |
| KLF63 | 3.14E-59 | 0.278384 | 0.917 | 0.663 | 7.08E-55 | 10 |
| MS4A1 | 0 | 2.014288 | 0.757 | 0.023 | 0 | 11 |
| CD372 | 0 | 1.332313 | 0.903 | 0.426 | 0 | 11 |
| CCR71 | 0 | 1.230417 | 0.554 | 0.092 | 0 | 11 |
| ADAM281 | 0 | 0.971798 | 0.377 | 0.043 | 0 | 11 |
| CD744 | 1.91E-252 | 0.924054 | 1 | 0.898 | 4.30E-248 | 11 |
| EZR2 | 2.06E-237 | 1.146917 | 0.887 | 0.485 | 4.65E-233 | 11 |
| CD834 | 6.69E-157 | 0.986595 | 0.69 | 0.298 | 1.51E-152 | 11 |
| CXCR43 | 4.41E-65 | 0.42365 | 0.98 | 0.804 | 9.95E-61 | 11 |
| PARP1 | 5.51E-17 | 0.329172 | 0.325 | 0.224 | 1.24E-12 | 11 |
| LYZ3 | 0 | 2.972081 | 0.897 | 0.353 | 0 | 12 |
| CD242 | 0 | 1.821331 | 0.73 | 0.092 | 0 | 12 |
| KRT182 | 0 | 1.770923 | 0.889 | 0.208 | 0 | 12 |
| ALDH1A13 | 0 | 1.635478 | 0.889 | 0.214 | 0 | 12 |
| TM4SF12 | 0 | 1.328542 | 0.764 | 0.186 | 0 | 12 |
| DKK1 | 0 | 1.125119 | 0.381 | 0.002 | 0 | 12 |
| AFP | 0 | 0.55944 | 0.364 | 0.018 | 0 | 12 |
| AOX11 | 2.61E-286 | 0.332009 | 0.303 | 0.034 | 5.89E-282 | 12 |
| CDH21 | 6.22E-272 | 0.278314 | 0.271 | 0.029 | 1.40E-267 | 12 |
| ANPEP1 | 4.03E-250 | 0.497849 | 0.434 | 0.076 | 9.09E-246 | 12 |
| ACTL6A1 | 5.24E-211 | 0.48316 | 0.446 | 0.091 | 1.18E-206 | 12 |
| AADAC2 | 1.17E-182 | 0.551682 | 0.407 | 0.086 | 2.63E-178 | 12 |
| CD461 | 4.36E-172 | 0.69486 | 0.62 | 0.201 | 9.83E-168 | 12 |
| VCAM11 | 2.59E-138 | 0.57493 | 0.531 | 0.159 | 5.84E-134 | 12 |
| LGALS33 | 3.18E-134 | 0.738126 | 0.714 | 0.304 | 7.17E-130 | 12 |
| HPRT1 | 5.71E-130 | 0.476428 | 0.593 | 0.201 | 1.29E-125 | 12 |
| SDC12 | 4.64E-117 | 0.310294 | 0.326 | 0.078 | 1.05E-112 | 12 |
| TF3 | 1.20E-110 | 0.661453 | 0.629 | 0.25 | 2.71E-106 | 12 |
| ACAT22 | 5.58E-108 | 0.483094 | 0.424 | 0.13 | 1.26E-103 | 12 |
| ACTB3 | 8.24E-104 | 0.463057 | 0.993 | 0.991 | 1.86E-99 | 12 |
| ADI12 | 3.99E-78 | 0.35692 | 0.694 | 0.309 | 9.01E-74 | 12 |
| PARP11 | 5.01E-77 | 0.346262 | 0.546 | 0.221 | 1.13E-72 | 12 |
| SLC2A22 | 8.19E-49 | 0.344077 | 0.266 | 0.095 | 1.85E-44 | 12 |
| FXYD2 | 0 | 1.539401 | 0.755 | 0.037 | 0 | 13 |
| EPCAM | 0 | 1.432718 | 0.671 | 0.018 | 0 | 13 |
| CD243 | 0 | 1.210355 | 0.725 | 0.092 | 0 | 13 |
| KRT19 | 0 | 1.202632 | 0.564 | 0.017 | 0 | 13 |
| KRT183 | 0 | 1.197091 | 0.874 | 0.208 | 0 | 13 |
| CLDN6 | 0 | 1.029619 | 0.509 | 0.007 | 0 | 13 |
| UCHL1 | 0 | 0.599489 | 0.333 | 0.019 | 0 | 13 |
| TF4 | 1.09E-278 | 0.998933 | 0.801 | 0.247 | 2.46E-274 | 13 |
| CYP17A12 | 9.67E-273 | 1.043064 | 0.433 | 0.074 | 2.18E-268 | 13 |
| ENO2 | 2.93E-240 | 0.383528 | 0.259 | 0.03 | 6.60E-236 | 13 |
| CD94 | 9.81E-141 | 0.555071 | 0.652 | 0.237 | 2.21E-136 | 13 |
| ADI13 | 9.91E-111 | 0.590194 | 0.69 | 0.309 | 2.24E-106 | 13 |
| ACTB4 | 7.52E-93 | 0.388615 | 0.999 | 0.991 | 1.70E-88 | 13 |
| ACTL6A2 | 1.18E-78 | 0.33322 | 0.308 | 0.094 | 2.66E-74 | 13 |
| ANP32E1 | 7.27E-39 | 0.289434 | 0.436 | 0.214 | 1.64E-34 | 13 |
| ACAT23 | 8.10E-37 | 0.254746 | 0.303 | 0.132 | 1.83E-32 | 13 |
| PARP12 | 8.55E-33 | 0.294866 | 0.419 | 0.223 | 1.93E-28 | 13 |
| ITGB13 | 1.49E-32 | 0.304265 | 0.635 | 0.417 | 3.36E-28 | 13 |
| CPM3 | 0 | 1.434276 | 0.784 | 0.151 | 0 | 14 |
| CD684 | 0 | 1.320402 | 0.916 | 0.271 | 0 | 14 |
| AIF14 | 0 | 1.286746 | 0.994 | 0.333 | 0 | 14 |
| CD1633 | 0 | 1.097799 | 0.851 | 0.178 | 0 | 14 |
| ADORA31 | 0 | 0.508684 | 0.37 | 0.033 | 0 | 14 |
| CD745 | 1.22E-292 | 1.428943 | 1 | 0.898 | 2.75E-288 | 14 |
| VCAM12 | 1.29E-253 | 1.208307 | 0.649 | 0.158 | 2.90E-249 | 14 |
| A2M2 | 1.30E-253 | 0.890861 | 0.795 | 0.238 | 2.94E-249 | 14 |
| AXL3 | 4.36E-252 | 0.68969 | 0.595 | 0.123 | 9.83E-248 | 14 |
| APOE3 | 1.85E-220 | 1.091982 | 0.978 | 0.583 | 4.17E-216 | 14 |
| CD145 | 5.42E-220 | 0.77348 | 0.765 | 0.227 | 1.22E-215 | 14 |
| CYP2C91 | 2.06E-198 | 0.374991 | 0.364 | 0.06 | 4.65E-194 | 14 |
| CCL34 | 5.16E-163 | 1.194525 | 0.901 | 0.493 | 1.16E-158 | 14 |
| MITF | 3.03E-157 | 0.319903 | 0.288 | 0.048 | 6.83E-153 | 14 |
| GLUL4 | 4.01E-142 | 0.667503 | 0.917 | 0.514 | 9.04E-138 | 14 |
| FCGR3A4 | 5.95E-129 | 0.463801 | 0.744 | 0.262 | 1.34E-124 | 14 |
| ABCA13 | 1.30E-127 | 0.449464 | 0.417 | 0.108 | 2.93E-123 | 14 |
| NRP13 | 3.73E-126 | 0.432588 | 0.424 | 0.113 | 8.41E-122 | 14 |
| CD43 | 2.16E-113 | 0.41356 | 0.528 | 0.167 | 4.87E-109 | 14 |
| FABP52 | 5.27E-113 | 0.695424 | 0.76 | 0.342 | 1.19E-108 | 14 |
| RTN45 | 5.08E-104 | 0.58535 | 0.827 | 0.441 | 1.15E-99 | 14 |
| LGALS34 | 1.45E-80 | 0.459372 | 0.679 | 0.305 | 3.27E-76 | 14 |
| CD835 | 1.85E-62 | 0.514269 | 0.617 | 0.3 | 4.16E-58 | 14 |
| ADI14 | 7.28E-53 | 0.257362 | 0.636 | 0.311 | 1.64E-48 | 14 |
| KLF64 | 1.53E-36 | 0.377659 | 0.9 | 0.665 | 3.45E-32 | 14 |
| CD272 | 6.63E-234 | 0.532439 | 0.552 | 0.1 | 1.50E-229 | 15 |
| CD381 | 1.53E-213 | 0.46829 | 0.523 | 0.1 | 3.45E-209 | 15 |
| ACTA2 | 0 | 4.084989 | 0.973 | 0.104 | 0 | 16 |
| ADAMTS11 | 0 | 1.596229 | 0.764 | 0.046 | 0 | 16 |
| CNN1 | 0 | 1.571549 | 0.534 | 0.008 | 0 | 16 |
| ACTN12 | 0 | 1.384309 | 0.734 | 0.139 | 0 | 16 |
| ADAMTS41 | 0 | 1.364387 | 0.66 | 0.039 | 0 | 16 |
| COL1A2 | 0 | 1.32582 | 0.699 | 0.033 | 0 | 16 |
| MCAM | 0 | 1.276167 | 0.658 | 0.032 | 0 | 16 |
| THY1 | 0 | 1.26976 | 0.376 | 0.036 | 0 | 16 |
| ITGA1 | 0 | 1.084878 | 0.628 | 0.051 | 0 | 16 |
| COL1A1 | 0 | 0.73663 | 0.472 | 0.035 | 0 | 16 |
| NES | 0 | 0.551955 | 0.333 | 0.022 | 0 | 16 |
| CSPG4 | 0 | 0.495624 | 0.287 | 0.004 | 0 | 16 |
| DMD | 0 | 0.441323 | 0.287 | 0.023 | 0 | 16 |
| AXL4 | 1.92E-304 | 0.985772 | 0.647 | 0.123 | 4.33E-300 | 16 |
| CD95 | 5.91E-299 | 1.143828 | 0.863 | 0.235 | 1.33E-294 | 16 |
| AKAP121 | 1.91E-279 | 0.520343 | 0.5 | 0.077 | 4.31E-275 | 16 |
| ITGB14 | 1.81E-248 | 1.300759 | 0.911 | 0.414 | 4.07E-244 | 16 |
| LRRC321 | 5.84E-248 | 0.483394 | 0.309 | 0.034 | 1.32E-243 | 16 |
| A2M3 | 2.71E-204 | 0.839464 | 0.775 | 0.239 | 6.11E-200 | 16 |
| UBA2 | 1.12E-202 | 0.793844 | 0.608 | 0.154 | 2.54E-198 | 16 |
| MYC1 | 8.71E-135 | 1.107965 | 0.52 | 0.157 | 1.97E-130 | 16 |
| ABI2 | 9.29E-84 | 0.299239 | 0.298 | 0.075 | 2.10E-79 | 16 |
| ADI15 | 3.79E-74 | 0.394761 | 0.691 | 0.311 | 8.55E-70 | 16 |
| ANXA54 | 2.18E-69 | 0.464915 | 0.814 | 0.432 | 4.91E-65 | 16 |
| KRT184 | 5.12E-59 | 0.284229 | 0.528 | 0.215 | 1.16E-54 | 16 |
| NRP14 | 1.60E-58 | 0.332788 | 0.335 | 0.115 | 3.60E-54 | 16 |
| ACTB5 | 4.57E-55 | 0.369205 | 0.998 | 0.991 | 1.03E-50 | 16 |
| RTN46 | 1.30E-52 | 0.426499 | 0.766 | 0.442 | 2.94E-48 | 16 |
| CPM4 | 8.25E-40 | 0.323841 | 0.365 | 0.158 | 1.86E-35 | 16 |
| CD462 | 4.30E-36 | 0.285754 | 0.427 | 0.206 | 9.69E-32 | 16 |
| B2M3 | 3.63E-129 | 0.488711 | 1 | 0.999 | 8.18E-125 | 17 |
| ACTB6 | 1.54E-126 | 0.967043 | 0.993 | 0.991 | 3.47E-122 | 17 |
| CD273 | 9.34E-101 | 1.01122 | 0.363 | 0.103 | 2.11E-96 | 17 |
| EZR3 | 2.25E-90 | 1.089998 | 0.74 | 0.489 | 5.07E-86 | 17 |
| CD3D4 | 2.95E-73 | 0.894688 | 0.68 | 0.417 | 6.65E-69 | 17 |
| CXCR44 | 2.73E-70 | 0.666624 | 0.936 | 0.805 | 6.17E-66 | 17 |
| PTPRC4 | 2.55E-53 | 0.632054 | 0.762 | 0.601 | 5.75E-49 | 17 |
| CD444 | 6.93E-46 | 0.748006 | 0.651 | 0.494 | 1.56E-41 | 17 |
| IL7R2 | 1.12E-16 | 0.390939 | 0.454 | 0.301 | 2.53E-12 | 17 |
| PBXIP13 | 2.06E-13 | 0.556362 | 0.285 | 0.191 | 4.66E-09 | 17 |
| ABRACL2 | 2.25E-10 | 0.488974 | 0.434 | 0.405 | 5.08E-06 | 17 |
| CD3G4 | 4.88E-10 | 0.338288 | 0.313 | 0.221 | 1.10E-05 | 17 |
| KRT185 | 0 | 1.78101 | 0.859 | 0.211 | 0 | 18 |
| UCHL11 | 0 | 1.564404 | 0.562 | 0.017 | 0 | 18 |
| MAGEA4 | 0 | 1.326659 | 0.558 | 0.005 | 0 | 18 |
| AFP1 | 2.04E-305 | 1.059164 | 0.263 | 0.02 | 4.60E-301 | 18 |
| ALDH1A14 | 2.03E-210 | 1.285638 | 0.723 | 0.219 | 4.58E-206 | 18 |
| CD244 | 8.31E-193 | 1.065312 | 0.473 | 0.098 | 1.87E-188 | 18 |
| TF5 | 1.44E-162 | 1.35081 | 0.695 | 0.25 | 3.24E-158 | 18 |
| TM4SF13 | 1.54E-154 | 0.90344 | 0.619 | 0.19 | 3.47E-150 | 18 |
| ANPEP2 | 1.13E-97 | 0.50003 | 0.323 | 0.079 | 2.54E-93 | 18 |
| ANXA55 | 3.37E-44 | 0.628498 | 0.681 | 0.434 | 7.61E-40 | 18 |
| ACTL6A3 | 1.51E-42 | 0.316169 | 0.27 | 0.095 | 3.41E-38 | 18 |
| MYC2 | 2.21E-36 | 0.394052 | 0.354 | 0.159 | 4.99E-32 | 18 |
| NRP15 | 5.51E-32 | 0.255735 | 0.283 | 0.115 | 1.24E-27 | 18 |
| ACAT24 | 1.37E-20 | 0.29197 | 0.272 | 0.133 | 3.09E-16 | 18 |
| COL1A11 | 0 | 3.220302 | 0.885 | 0.03 | 0 | 19 |
| COL1A21 | 0 | 3.079271 | 0.93 | 0.031 | 0 | 19 |
| THY11 | 0 | 2.17489 | 0.79 | 0.031 | 0 | 19 |
| ACTA21 | 0 | 1.881639 | 0.784 | 0.107 | 0 | 19 |
| ECM1 | 0 | 1.439319 | 0.566 | 0.025 | 0 | 19 |
| COL5A1 | 0 | 1.047607 | 0.508 | 0.007 | 0 | 19 |
| ITGA11 | 0 | 0.958363 | 0.589 | 0.052 | 0 | 19 |
| FAP | 0 | 0.682122 | 0.344 | 0.005 | 0 | 19 |
| PDGFRA | 0 | 0.663869 | 0.311 | 0.004 | 0 | 19 |
| CDH11 | 0 | 0.59995 | 0.35 | 0.006 | 0 | 19 |
| ABCC9 | 0 | 0.58384 | 0.265 | 0.009 | 0 | 19 |
| ADAMTS2 | 0 | 0.478936 | 0.3 | 0.009 | 0 | 19 |
| LRRC322 | 4.44E-305 | 0.572725 | 0.352 | 0.034 | 1.00E-300 | 19 |
| ACTN13 | 9.65E-274 | 0.994278 | 0.66 | 0.14 | 2.18E-269 | 19 |
| ITGB15 | 1.58E-242 | 1.359766 | 0.92 | 0.415 | 3.56E-238 | 19 |
| ENG1 | 6.27E-150 | 0.511294 | 0.459 | 0.1 | 1.41E-145 | 19 |
| ADAMTS12 | 2.43E-149 | 0.611893 | 0.321 | 0.052 | 5.49E-145 | 19 |
| ANXA56 | 1.70E-136 | 0.784762 | 0.901 | 0.431 | 3.85E-132 | 19 |
| NRP16 | 9.96E-123 | 0.520361 | 0.447 | 0.113 | 2.25E-118 | 19 |
| CD97 | 1.79E-100 | 0.731262 | 0.638 | 0.239 | 4.03E-96 | 19 |
| KRT186 | 2.12E-96 | 0.495622 | 0.623 | 0.214 | 4.78E-92 | 19 |
| A2M4 | 2.46E-93 | 0.544684 | 0.636 | 0.242 | 5.54E-89 | 19 |
| ABI21 | 7.84E-76 | 0.315668 | 0.296 | 0.075 | 1.77E-71 | 19 |
| AXL5 | 8.92E-74 | 0.449552 | 0.407 | 0.127 | 2.01E-69 | 19 |
| IL6ST1 | 1.63E-72 | 0.477723 | 0.523 | 0.199 | 3.68E-68 | 19 |
| ADI16 | 6.56E-68 | 0.404092 | 0.693 | 0.311 | 1.48E-63 | 19 |
| RTN47 | 1.37E-54 | 0.468599 | 0.772 | 0.442 | 3.08E-50 | 19 |
| CD992 | 1.67E-49 | 0.489627 | 0.815 | 0.545 | 3.76E-45 | 19 |
| MYC3 | 9.10E-44 | 0.554991 | 0.383 | 0.159 | 2.05E-39 | 19 |
| ALDH1A15 | 3.81E-12 | 0.535148 | 0.342 | 0.224 | 8.60E-08 | 19 |
| CD685 | 0 | 1.225618 | 0.992 | 0.272 | 0 | 20 |
| ALDH1L12 | 0 | 0.71944 | 0.641 | 0.063 | 0 | 20 |
| CD209 | 0 | 0.484735 | 0.345 | 0.024 | 0 | 20 |
| AIF15 | 5.78E-285 | 1.258146 | 0.998 | 0.335 | 1.30E-280 | 20 |
| FABP53 | 1.16E-284 | 1.540956 | 0.961 | 0.341 | 2.62E-280 | 20 |
| GLUL5 | 1.10E-279 | 1.455682 | 0.996 | 0.514 | 2.49E-275 | 20 |
| ABCA14 | 1.07E-241 | 0.591485 | 0.58 | 0.107 | 2.41E-237 | 20 |
| CD1634 | 1.17E-238 | 0.593023 | 0.801 | 0.181 | 2.63E-234 | 20 |
| ALDH1A16 | 2.38E-234 | 0.697079 | 0.836 | 0.218 | 5.38E-230 | 20 |
| CD45 | 5.03E-221 | 0.628207 | 0.722 | 0.166 | 1.14E-216 | 20 |
| A2M5 | 2.12E-200 | 0.650855 | 0.842 | 0.239 | 4.78E-196 | 20 |
| LGALS35 | 2.09E-198 | 0.889269 | 0.892 | 0.303 | 4.70E-194 | 20 |
| APOE4 | 3.53E-195 | 0.955676 | 0.984 | 0.584 | 7.96E-191 | 20 |
| CPM5 | 5.37E-186 | 0.579519 | 0.651 | 0.155 | 1.21E-181 | 20 |
| CD146 | 7.96E-185 | 0.781163 | 0.787 | 0.229 | 1.80E-180 | 20 |
| TF6 | 3.23E-182 | 0.304532 | 0.878 | 0.248 | 7.29E-178 | 20 |
| CD746 | 9.49E-172 | 0.985219 | 1 | 0.898 | 2.14E-167 | 20 |
| FCGR3A5 | 1.61E-145 | 0.501821 | 0.838 | 0.263 | 3.64E-141 | 20 |
| NRP17 | 1.15E-144 | 0.393065 | 0.493 | 0.113 | 2.58E-140 | 20 |
| SLC2A23 | 5.85E-142 | 0.282252 | 0.456 | 0.094 | 1.32E-137 | 20 |
| MITF1 | 3.81E-140 | 0.283045 | 0.302 | 0.049 | 8.60E-136 | 20 |
| VCAM13 | 5.89E-137 | 0.719957 | 0.592 | 0.16 | 1.33E-132 | 20 |
| ALCAM | 9.54E-135 | 0.3441 | 0.365 | 0.071 | 2.15E-130 | 20 |
| ITGAX3 | 5.90E-125 | 0.285043 | 0.359 | 0.071 | 1.33E-120 | 20 |
| TFRC2 | 2.59E-121 | 0.517738 | 0.469 | 0.118 | 5.85E-117 | 20 |
| SDC13 | 2.21E-116 | 0.308982 | 0.365 | 0.078 | 4.99E-112 | 20 |
| CD402 | 2.40E-96 | 0.334327 | 0.387 | 0.099 | 5.42E-92 | 20 |
| CCL35 | 2.75E-93 | 0.439919 | 0.878 | 0.494 | 6.21E-89 | 20 |
| RTN48 | 7.18E-90 | 0.522002 | 0.895 | 0.441 | 1.62E-85 | 20 |
| ADI17 | 1.84E-83 | 0.336404 | 0.777 | 0.31 | 4.16E-79 | 20 |
| CD836 | 1.12E-68 | 0.280777 | 0.736 | 0.3 | 2.52E-64 | 20 |
| ICAM14 | 9.04E-67 | 0.306581 | 0.6 | 0.231 | 2.04E-62 | 20 |
| ACTB7 | 4.53E-39 | 0.257548 | 1 | 0.991 | 1.02E-34 | 20 |
| CYP2C92 | 0 | 1.766941 | 0.67 | 0.058 | 0 | 21 |
| AOX12 | 0 | 0.966084 | 0.446 | 0.034 | 0 | 21 |
| ADI18 | 1.39E-292 | 1.802464 | 0.898 | 0.309 | 3.13E-288 | 21 |
| AADAC3 | 6.75E-268 | 1.137635 | 0.541 | 0.086 | 1.52E-263 | 21 |
| ALDH1A17 | 6.33E-213 | 1.297683 | 0.757 | 0.22 | 1.43E-208 | 21 |
| ACADSB1 | 5.87E-196 | 0.829838 | 0.402 | 0.063 | 1.32E-191 | 21 |
| TF7 | 1.73E-172 | 1.45845 | 0.752 | 0.251 | 3.91E-168 | 21 |
| ALDH1L13 | 6.65E-170 | 0.693008 | 0.393 | 0.066 | 1.50E-165 | 21 |
| SLC2A24 | 6.86E-168 | 0.820963 | 0.474 | 0.094 | 1.55E-163 | 21 |
| APOE5 | 7.86E-155 | 0.885851 | 0.993 | 0.584 | 1.77E-150 | 21 |
| KRT187 | 6.55E-140 | 0.776289 | 0.696 | 0.214 | 1.48E-135 | 21 |
| A1BG2 | 1.90E-136 | 1.298145 | 0.659 | 0.235 | 4.28E-132 | 21 |
| ADK | 3.72E-79 | 0.546722 | 0.37 | 0.106 | 8.40E-75 | 21 |
| ACADS1 | 2.03E-78 | 0.556343 | 0.311 | 0.079 | 4.59E-74 | 21 |
| SDC14 | 1.37E-51 | 0.431876 | 0.267 | 0.08 | 3.08E-47 | 21 |
| CD147 | 7.42E-38 | 0.278096 | 0.496 | 0.233 | 1.67E-33 | 21 |
| RTN49 | 2.92E-30 | 0.51106 | 0.652 | 0.444 | 6.59E-26 | 21 |
| ACAT25 | 7.97E-20 | 0.274419 | 0.276 | 0.133 | 1.80E-15 | 21 |
| ENG2 | 0 | 1.833348 | 0.896 | 0.097 | 0 | 22 |
| FLT11 | 0 | 1.587727 | 0.867 | 0.056 | 0 | 22 |
| VWF1 | 0 | 1.50815 | 0.8 | 0.025 | 0 | 22 |
| CD341 | 0 | 1.498095 | 0.878 | 0.025 | 0 | 22 |
| PECAM11 | 0 | 1.479765 | 0.91 | 0.131 | 0 | 22 |
| TM4SF14 | 0 | 1.446648 | 0.945 | 0.19 | 0 | 22 |
| ITGA61 | 0 | 1.207929 | 0.774 | 0.041 | 0 | 22 |
| THY12 | 0 | 1.133957 | 0.681 | 0.035 | 0 | 22 |
| CDH52 | 0 | 1.0265 | 0.748 | 0.045 | 0 | 22 |
| AKAP122 | 0 | 1.007785 | 0.646 | 0.078 | 0 | 22 |
| ABCG21 | 0 | 0.962146 | 0.612 | 0.033 | 0 | 22 |
| ADAM151 | 0 | 0.784164 | 0.6 | 0.067 | 0 | 22 |
| IL3RA1 | 0 | 0.748795 | 0.557 | 0.03 | 0 | 22 |
| TSPAN71 | 0 | 0.70041 | 0.516 | 0.049 | 0 | 22 |
| ACVRL11 | 0 | 0.635335 | 0.522 | 0.041 | 0 | 22 |
| NES1 | 0 | 0.564924 | 0.42 | 0.023 | 0 | 22 |
| CD200 | 0 | 0.349732 | 0.272 | 0.014 | 0 | 22 |
| A4GALT | 0 | 0.347767 | 0.304 | 0.012 | 0 | 22 |
| CD98 | 2.37E-269 | 1.518596 | 0.954 | 0.238 | 5.36E-265 | 22 |
| A2M6 | 6.68E-260 | 1.506123 | 0.936 | 0.241 | 1.51E-255 | 22 |
| MCAM1 | 7.60E-221 | 0.419518 | 0.383 | 0.038 | 1.72E-216 | 22 |
| ITGA51 | 7.99E-216 | 0.544805 | 0.539 | 0.074 | 1.80E-211 | 22 |
| ITGA12 | 6.55E-205 | 0.467107 | 0.452 | 0.055 | 1.48E-200 | 22 |
| NRP18 | 3.93E-189 | 0.680947 | 0.623 | 0.113 | 8.86E-185 | 22 |
| LRRC323 | 5.05E-187 | 0.456514 | 0.342 | 0.036 | 1.14E-182 | 22 |
| CYP2C93 | 4.66E-163 | 0.398549 | 0.435 | 0.062 | 1.05E-158 | 22 |
| IL6ST2 | 9.03E-159 | 0.837502 | 0.762 | 0.198 | 2.04E-154 | 22 |
| ITGB16 | 2.70E-146 | 0.999688 | 0.948 | 0.416 | 6.08E-142 | 22 |
| CTNNB12 | 3.38E-142 | 1.132063 | 0.907 | 0.335 | 7.63E-138 | 22 |
| CD403 | 8.23E-128 | 0.45136 | 0.499 | 0.099 | 1.86E-123 | 22 |
| ABCG1 | 5.65E-122 | 0.424611 | 0.42 | 0.075 | 1.27E-117 | 22 |
| CPM6 | 8.27E-79 | 0.456732 | 0.548 | 0.158 | 1.87E-74 | 22 |
| ADAMTS13 | 1.20E-70 | 0.304578 | 0.281 | 0.054 | 2.71E-66 | 22 |
| ABI22 | 1.06E-68 | 0.274315 | 0.336 | 0.076 | 2.39E-64 | 22 |
| CD993 | 2.04E-64 | 0.556359 | 0.928 | 0.545 | 4.59E-60 | 22 |
| RTN410 | 1.41E-50 | 0.506078 | 0.841 | 0.443 | 3.18E-46 | 22 |
| CD463 | 2.52E-39 | 0.263312 | 0.525 | 0.206 | 5.69E-35 | 22 |
| KLF65 | 4.26E-24 | 0.349799 | 0.901 | 0.667 | 9.60E-20 | 22 |
| ID22 | 1.48E-19 | 0.385065 | 0.846 | 0.689 | 3.34E-15 | 22 |
| CCNA2 | 6.87E-308 | 0.526513 | 0.291 | 0.015 | 1.55E-303 | 23 |
| CDC20 | 1.80E-203 | 0.695104 | 0.337 | 0.031 | 4.05E-199 | 23 |
| ANP32E2 | 1.60E-145 | 0.993783 | 0.768 | 0.214 | 3.60E-141 | 23 |
| ACTB8 | 8.46E-114 | 0.885369 | 1 | 0.991 | 1.91E-109 | 23 |
| CD382 | 5.95E-95 | 0.746786 | 0.449 | 0.103 | 1.34E-90 | 23 |
| KLRD12 | 3.27E-56 | 0.672857 | 0.672 | 0.271 | 7.38E-52 | 23 |
| SPN1 | 3.50E-45 | 0.38495 | 0.452 | 0.149 | 7.89E-41 | 23 |
| HPRT11 | 4.78E-44 | 0.483037 | 0.511 | 0.205 | 1.08E-39 | 23 |
| CD994 | 1.72E-35 | 0.53308 | 0.82 | 0.546 | 3.87E-31 | 23 |
| PARP13 | 5.43E-30 | 0.393464 | 0.486 | 0.224 | 1.22E-25 | 23 |
| ACAT26 | 1.89E-25 | 0.281022 | 0.334 | 0.133 | 4.26E-21 | 23 |
| KLRB12 | 4.97E-25 | 0.266854 | 0.715 | 0.384 | 1.12E-20 | 23 |
| PTPRC5 | 1.72E-24 | 0.293157 | 0.885 | 0.601 | 3.87E-20 | 23 |
| ABRACL3 | 1.57E-22 | 0.328553 | 0.687 | 0.403 | 3.53E-18 | 23 |
| CXCR45 | 7.89E-21 | 0.287607 | 0.988 | 0.806 | 1.78E-16 | 23 |
| ID23 | 6.25E-13 | 0.250416 | 0.879 | 0.689 | 1.41E-08 | 23 |
| IL7R3 | 5.10E-74 | 0.944035 | 0.734 | 0.299 | 1.15E-69 | 24 |
| CYP2C94 | 1.27E-44 | 0.515099 | 0.253 | 0.064 | 2.86E-40 | 24 |
| CD3D5 | 1.60E-34 | 0.484708 | 0.766 | 0.418 | 3.61E-30 | 24 |
| KLRB13 | 3.27E-32 | 0.743563 | 0.696 | 0.384 | 7.37E-28 | 24 |
| CCR61 | 1.67E-29 | 0.428101 | 0.291 | 0.097 | 3.76E-25 | 24 |
| CXCR46 | 4.99E-24 | 0.354757 | 0.956 | 0.806 | 1.13E-19 | 24 |
| CD3G5 | 2.42E-21 | 0.422801 | 0.459 | 0.22 | 5.46E-17 | 24 |
| PTPRC6 | 1.25E-12 | 0.255416 | 0.769 | 0.602 | 2.83E-08 | 24 |
| KLF66 | 9.26E-11 | 0.335138 | 0.759 | 0.668 | 2.09E-06 | 24 |
| ANXA16 | 1.96E-08 | 0.256115 | 0.687 | 0.606 | 0.000442991 | 24 |
| AAK13 | 4.44E-08 | 0.377348 | 0.383 | 0.264 | 0.001002669 | 24 |
| MS4A11 | 0 | 1.980586 | 0.873 | 0.03 | 0 | 25 |
| CD19 | 0 | 0.665802 | 0.286 | 0.011 | 0 | 25 |
| ADAM282 | 6.39E-278 | 1.05579 | 0.47 | 0.046 | 1.44E-273 | 25 |
| CCR72 | 3.04E-201 | 1.101597 | 0.613 | 0.097 | 6.86E-197 | 25 |
| CD373 | 1.16E-124 | 1.118929 | 0.921 | 0.431 | 2.61E-120 | 25 |
| CD837 | 8.48E-105 | 1.063821 | 0.819 | 0.301 | 1.91E-100 | 25 |
| CD747 | 2.24E-89 | 0.691473 | 1 | 0.899 | 5.05E-85 | 25 |
| EZR4 | 5.66E-89 | 1.025481 | 0.898 | 0.489 | 1.28E-84 | 25 |
| CD245 | 1.47E-57 | 0.541378 | 0.371 | 0.101 | 3.31E-53 | 25 |
| CD274 | 1.66E-49 | 0.448543 | 0.371 | 0.104 | 3.75E-45 | 25 |
| KLF67 | 2.67E-30 | 0.46258 | 0.898 | 0.667 | 6.03E-26 | 25 |
| PARP15 | 2.59E-19 | 0.410652 | 0.413 | 0.225 | 5.84E-15 | 25 |
| ALCAM1 | 5.87E-192 | 0.914435 | 0.509 | 0.072 | 1.32E-187 | 26 |
| CD1635 | 3.79E-185 | 1.287719 | 0.805 | 0.184 | 8.55E-181 | 26 |
| CD686 | 9.55E-165 | 1.314647 | 0.918 | 0.276 | 2.16E-160 | 26 |
| CD148 | 4.84E-157 | 1.458278 | 0.823 | 0.231 | 1.09E-152 | 26 |
| AIF16 | 7.26E-141 | 1.176334 | 0.966 | 0.338 | 1.64E-136 | 26 |
| CD748 | 1.15E-130 | 1.430203 | 1 | 0.899 | 2.60E-126 | 26 |
| LYZ4 | 3.88E-114 | 1.099686 | 0.908 | 0.358 | 8.75E-110 | 26 |
| ANXA57 | 1.10E-105 | 1.02987 | 0.904 | 0.434 | 2.49E-101 | 26 |
| CPM7 | 3.63E-98 | 0.869495 | 0.59 | 0.158 | 8.20E-94 | 26 |
| CD910 | 1.69E-89 | 0.984861 | 0.717 | 0.24 | 3.80E-85 | 26 |
| GLUL6 | 5.47E-84 | 1.005556 | 0.939 | 0.517 | 1.23E-79 | 26 |
| ACTB9 | 1.49E-78 | 0.824301 | 1 | 0.991 | 3.35E-74 | 26 |
| A2M7 | 2.04E-75 | 0.915343 | 0.662 | 0.244 | 4.61E-71 | 26 |
| CD838 | 1.74E-74 | 0.986948 | 0.754 | 0.302 | 3.92E-70 | 26 |
| LGALS36 | 1.11E-62 | 0.764186 | 0.72 | 0.308 | 2.51E-58 | 26 |
| AXL6 | 6.75E-58 | 0.626824 | 0.444 | 0.128 | 1.52E-53 | 26 |
| FPR13 | 2.14E-49 | 0.542142 | 0.341 | 0.092 | 4.84E-45 | 26 |
| FABP54 | 3.24E-48 | 0.695158 | 0.727 | 0.346 | 7.30E-44 | 26 |
| CD49 | 3.41E-32 | 0.439845 | 0.43 | 0.171 | 7.68E-28 | 26 |
| ITGAX4 | 2.16E-31 | 0.326567 | 0.253 | 0.073 | 4.87E-27 | 26 |
| ICAM15 | 3.05E-27 | 0.713785 | 0.478 | 0.233 | 6.88E-23 | 26 |
| FCGR3A6 | 2.41E-26 | 0.335751 | 0.577 | 0.268 | 5.44E-22 | 26 |
| ABCA15 | 3.51E-25 | 0.398631 | 0.297 | 0.112 | 7.91E-21 | 26 |
| CCL36 | 1.33E-24 | 1.517803 | 0.669 | 0.498 | 3.01E-20 | 26 |
| NRP19 | 5.14E-19 | 0.379965 | 0.273 | 0.117 | 1.16E-14 | 26 |
| BCL2A13 | 5.11E-18 | 0.606168 | 0.474 | 0.268 | 1.15E-13 | 26 |
| TFRC3 | 2.77E-13 | 0.348438 | 0.259 | 0.122 | 6.24E-09 | 26 |
| ACTA22 | 0 | 3.018546 | 0.907 | 0.112 | 0 | 27 |
| COL1A22 | 0 | 2.315276 | 0.868 | 0.038 | 0 | 27 |
| COL1A12 | 0 | 2.292595 | 0.834 | 0.037 | 0 | 27 |
| THY13 | 0 | 2.157698 | 0.639 | 0.038 | 0 | 27 |
| ADAMTS4 | 0 | 1.346264 | 0.649 | 0.045 | 0 | 27 |
| CNN11 | 2.92E-195 | 0.907778 | 0.273 | 0.014 | 6.59E-191 | 27 |
| ITGA13 | 1.59E-155 | 0.74068 | 0.493 | 0.057 | 3.58E-151 | 27 |
| ECM11 | 2.56E-143 | 0.616984 | 0.346 | 0.031 | 5.78E-139 | 27 |
| MCAM2 | 2.83E-141 | 0.594337 | 0.39 | 0.039 | 6.38E-137 | 27 |
| LRRC324 | 3.61E-126 | 0.663784 | 0.356 | 0.037 | 8.15E-122 | 27 |
| ADAMTS1 | 7.30E-100 | 0.634448 | 0.4 | 0.054 | 1.65E-95 | 27 |
| AXL7 | 1.08E-73 | 0.922536 | 0.551 | 0.129 | 2.44E-69 | 27 |
| ITGB17 | 6.74E-66 | 1.047948 | 0.854 | 0.419 | 1.52E-61 | 27 |
| MYC4 | 5.31E-64 | 1.178285 | 0.571 | 0.16 | 1.20E-59 | 27 |
| A2M8 | 3.70E-62 | 0.716754 | 0.732 | 0.244 | 8.34E-58 | 27 |
| CD911 | 2.71E-61 | 0.993168 | 0.707 | 0.242 | 6.11E-57 | 27 |
| KRT188 | 1.08E-55 | 0.559513 | 0.693 | 0.217 | 2.43E-51 | 27 |
| ACTN14 | 2.35E-36 | 0.60707 | 0.444 | 0.146 | 5.30E-32 | 27 |
| ANXA58 | 5.32E-35 | 0.618733 | 0.82 | 0.435 | 1.20E-30 | 27 |
| UBA21 | 7.68E-35 | 0.553747 | 0.473 | 0.159 | 1.73E-30 | 27 |
| ACTB10 | 7.61E-32 | 0.533638 | 1 | 0.991 | 1.72E-27 | 27 |
| CD995 | 4.94E-28 | 0.549219 | 0.859 | 0.547 | 1.11E-23 | 27 |
| NRP110 | 1.61E-24 | 0.34971 | 0.346 | 0.117 | 3.63E-20 | 27 |
| ABI23 | 5.87E-23 | 0.276867 | 0.263 | 0.077 | 1.32E-18 | 27 |
| CPM8 | 9.85E-21 | 0.409084 | 0.4 | 0.16 | 2.22E-16 | 27 |
| ALDH1L14 | 1.79E-101 | 0.647951 | 0.48 | 0.068 | 4.04E-97 | 28 |
| CD275 | 1.42E-95 | 0.768142 | 0.606 | 0.104 | 3.20E-91 | 28 |
| TF8 | 1.91E-64 | 0.350186 | 0.869 | 0.254 | 4.31E-60 | 28 |
| CDC201 | 7.16E-64 | 0.485932 | 0.263 | 0.033 | 1.61E-59 | 28 |
| ACTB11 | 1.16E-58 | 0.858964 | 1 | 0.991 | 2.61E-54 | 28 |
| TNFRSF18 | 8.31E-55 | 0.579255 | 0.343 | 0.058 | 1.87E-50 | 28 |
| SLC2A25 | 2.72E-39 | 0.283772 | 0.411 | 0.097 | 6.13E-35 | 28 |
| CD61 | 1.44E-35 | 0.305967 | 0.423 | 0.105 | 3.25E-31 | 28 |
| PARP16 | 3.31E-33 | 0.523028 | 0.6 | 0.225 | 7.47E-29 | 28 |
| CD3D6 | 1.88E-32 | 0.478296 | 0.92 | 0.418 | 4.24E-28 | 28 |
| ALDH1A18 | 1.73E-31 | 0.32606 | 0.623 | 0.224 | 3.91E-27 | 28 |
| CD5 | 1.33E-27 | 0.256212 | 0.28 | 0.065 | 3.00E-23 | 28 |
| HPRT12 | 3.16E-27 | 0.381353 | 0.554 | 0.206 | 7.14E-23 | 28 |
| ACAT27 | 1.18E-25 | 0.326437 | 0.411 | 0.134 | 2.66E-21 | 28 |
| ACTL6A4 | 1.84E-23 | 0.295041 | 0.32 | 0.096 | 4.14E-19 | 28 |
| ANP32E3 | 6.78E-21 | 0.450089 | 0.52 | 0.217 | 1.53E-16 | 28 |
| GLUL7 | 1.97E-19 | 0.348621 | 0.817 | 0.519 | 4.45E-15 | 28 |
| ABRACL4 | 7.19E-19 | 0.361241 | 0.766 | 0.404 | 1.62E-14 | 28 |
| CD996 | 7.23E-19 | 0.413075 | 0.863 | 0.547 | 1.63E-14 | 28 |
| IL3RA2 | 0 | 1.48515 | 0.691 | 0.032 | 0 | 29 |
| CLEC4C | 0 | 0.742302 | 0.338 | 0.001 | 0 | 29 |
| EZR5 | 5.38E-57 | 1.180346 | 0.957 | 0.491 | 1.21E-52 | 29 |
| BLNK | 1.13E-56 | 0.474829 | 0.259 | 0.03 | 2.55E-52 | 29 |
| CD749 | 2.51E-53 | 1.040077 | 1 | 0.899 | 5.67E-49 | 29 |
| ADAM19 | 1.06E-43 | 0.456293 | 0.288 | 0.044 | 2.38E-39 | 29 |
| CD839 | 1.27E-31 | 0.899482 | 0.727 | 0.304 | 2.87E-27 | 29 |
| SPN2 | 4.47E-30 | 0.552338 | 0.511 | 0.15 | 1.01E-25 | 29 |
| CXCR47 | 9.33E-24 | 0.617662 | 0.993 | 0.806 | 2.10E-19 | 29 |
| CCR73 | 3.14E-17 | 0.795189 | 0.317 | 0.1 | 7.09E-13 | 29 |
| CD374 | 3.65E-13 | 0.400931 | 0.755 | 0.434 | 8.24E-09 | 29 |
| CD410 | 1.64E-10 | 0.328486 | 0.381 | 0.172 | 3.69E-06 | 29 |
| KRT7 | 0 | 2.416234 | 0.816 | 0.013 | 0 | 30 |
| FXYD21 | 4.86E-302 | 3.192851 | 0.934 | 0.048 | 1.10E-297 | 30 |
| KRT191 | 5.88E-273 | 2.104628 | 0.671 | 0.026 | 1.33E-268 | 30 |
| EPCAM1 | 2.61E-263 | 1.432901 | 0.697 | 0.028 | 5.88E-259 | 30 |
| ARL141 | 2.06E-252 | 0.835963 | 0.329 | 0.006 | 4.65E-248 | 30 |
| SOX9 | 4.85E-247 | 1.007029 | 0.526 | 0.017 | 1.09E-242 | 30 |
| CDH1 | 2.91E-210 | 0.638085 | 0.408 | 0.012 | 6.57E-206 | 30 |
| CD246 | 1.12E-122 | 1.990254 | 0.868 | 0.102 | 2.52E-118 | 30 |
| KRT189 | 2.31E-86 | 2.66122 | 0.974 | 0.218 | 5.20E-82 | 30 |
| TM4SF15 | 3.47E-79 | 1.953982 | 0.947 | 0.195 | 7.82E-75 | 30 |
| ABCC3 | 3.41E-50 | 0.58398 | 0.395 | 0.045 | 7.70E-46 | 30 |
| CDH22 | 3.58E-48 | 0.533342 | 0.329 | 0.033 | 8.09E-44 | 30 |
| CPM9 | 2.93E-45 | 1.025799 | 0.724 | 0.16 | 6.61E-41 | 30 |
| ANPEP3 | 9.71E-43 | 0.918346 | 0.5 | 0.082 | 2.19E-38 | 30 |
| KLF69 | 1.34E-36 | 1.706575 | 0.947 | 0.669 | 3.02E-32 | 30 |
| AQP3 | 6.44E-35 | 0.787903 | 0.434 | 0.072 | 1.45E-30 | 30 |
| SDC15 | 7.17E-31 | 0.695798 | 0.434 | 0.081 | 1.62E-26 | 30 |
| SLC2A26 | 8.84E-23 | 0.725672 | 0.434 | 0.098 | 1.99E-18 | 30 |
| ITGB18 | 4.73E-14 | 0.738511 | 0.763 | 0.42 | 1.07E-09 | 30 |
| ALCAM2 | 4.58E-13 | 0.45313 | 0.289 | 0.075 | 1.03E-08 | 30 |
| ALDH1A19 | 3.79E-12 | 0.352075 | 0.579 | 0.225 | 8.56E-08 | 30 |
| ANXA59 | 4.95E-12 | 0.669437 | 0.763 | 0.437 | 1.12E-07 | 30 |
| LGALS37 | 1.53E-10 | 0.423941 | 0.645 | 0.31 | 3.45E-06 | 30 |
| CD464 | 2.09E-07 | 0.38289 | 0.447 | 0.208 | 0.00472490 | 30 |
| Notes: HCC, Hepatocellular Carcinoma; Pct.1, Marker gene expressed in single cluster cells; Pct.2, Marker gene expressed in total cells | | | | | | |
