# Supplementary material for: Heterogeneity induced GZMA-F2R communication inefficient impairs antitumor immunotherapy of PD-1 mAb through JAK2/STAT1 signal suppression in hepatocellular carcinoma
Source: Cell Death Dis. 2022 Mar 7;13(3):213. doi: 10.1038/s41419-022-04654-7 (PMC8901912; doi:10.1038/s41419-022-04654-7)
Supplement: Supplementary file 2 — Table S2 [file 41419_2022_4654_MOESM2_ESM.docx]

| Table S2： Tumor-specific marker genes of cytotoxic cells | | |
| --- | --- | --- |
| Pct.1 | Pct.2 | Genes |
| 0.778 | 0.143 | BATF |
| 0.478 | 0.074 | TNFRSF4 |
| 0.562 | 0.039 | CTLA4 |
| 0.468 | 0.061 | TNFRSF18 |
| 0.827 | 0.306 | CARD16 |
| 0.408 | 0.027 | IL2RA |
| 0.721 | 0.31 | PKM |
| 0.773 | 0.344 | CTSC |
| 0.435 | 0.03 | AC133644.2 |
| 0.71 | 0.196 | TIGIT |
| 0.932 | 0.571 | TRAC |
| 0.618 | 0.129 | CD27 |
| 0.446 | 0.043 | LAIR2 |
| 0.406 | 0.043 | NGFRAP1 |
| 0.587 | 0.152 | DUSP4 |
| 0.441 | 0.063 | SPP1 |
| 0.703 | 0.235 | ARID5B |
| 0.993 | 0.866 | IL32 |
| 0.973 | 0.759 | S100A4 |
| 0.958 | 0.764 | SAT1 |
| 0.541 | 0.139 | ICOS |
| 0.371 | 0.029 | TBC1D4 |
| 0.274 | 0.028 | GEM |
| 0.394 | 0.071 | DNPH1 |
| 0.275 | 0.013 | ICA1 |
| 0.362 | 0.062 | ACP5 |
| 0.981 | 0.89 | GAPDH |
| 0.251 | 0.011 | IL1R2 |
| 0.279 | 0.004 | FOXP3 |
| 0.256 | 0.024 | AC002331.1 |
| 1 | 1 | B2M |
| 0.507 | 0.138 | CORO1B |
| 0.416 | 0.09 | MIR4435-2HG |
| 0.4 | 0.083 | TNFRSF9 |
| 0.764 | 0.375 | LGALS1 |
| 0.261 | 0.04 | STAM |
| 0.739 | 0.342 | SERPINA1 |
| 0.836 | 0.543 | ENO1 |
| 0.725 | 0.343 | CACYBP |
| 0.57 | 0.212 | GBP2 |
| 0.67 | 0.3 | PMAIP1 |
| 0.316 | 0.065 | HTATIP2 |
| 0.321 | 0.066 | SELL |
| 0.69 | 0.34 | UGP2 |
| 0.256 | 0.046 | SPATS2L |
| 0.81 | 0.519 | TPI1 |
| 0.874 | 0.599 | CD2 |
| 0.828 | 0.556 | SOD1 |
| 0.969 | 0.911 | MYL6 |
| 0.602 | 0.277 | FKBP1A |
| 0.481 | 0.181 | RBPJ |
| 0.468 | 0.17 | PTTG1 |
| 0.349 | 0.103 | LGALS3 |
| 0.414 | 0.141 | RHBDD2 |
| 0.59 | 0.273 | LINC00152 |
| 0.998 | 0.996 | HLA-A |
| 0.672 | 0.369 | TXN |
| 0.382 | 0.133 | CD59 |
| 0.719 | 0.455 | ARPC1B |
| 0.557 | 0.275 | PGAM1 |
| 0.969 | 0.919 | ATP5E |
| 0.818 | 0.62 | SAMSN1 |
| 0.902 | 0.69 | CD3D |
| 0.696 | 0.427 | RPS27L |
| 0.507 | 0.235 | PRDX2 |
| 0.984 | 0.99 | FTL |
| 0.794 | 0.59 | IL2RG |
| 0.995 | 0.99 | ACTB |
| 1 | 1 | TMSB4X |
| 0.61 | 0.336 | PRDX1 |
| 0.339 | 0.124 | DYNC1I2 |
| 0.806 | 0.62 | ALDOA |
| 0.965 | 0.921 | SERF2 |
| 0.64 | 0.384 | UBE2B |
| 0.529 | 0.262 | NAMPT |
| 0.339 | 0.126 | ATOX1 |
| 0.862 | 0.689 | S100A6 |
| 0.977 | 0.935 | UBC |
| 0.887 | 0.765 | SRP14 |
| 0.521 | 0.271 | COX17 |
| 0.56 | 0.301 | LTB |
| 0.967 | 0.912 | OAZ1 |
| 0.651 | 0.402 | ATP5J2 |
| 0.747 | 0.478 | HSPA1A |
| 0.68 | 0.467 | PGK1 |
| 0.593 | 0.356 | HSPB1 |
| 0.578 | 0.331 | PSMB3 |
| 0.74 | 0.507 | DYNLL1 |
| 0.415 | 0.192 | CASP1 |
| 0.683 | 0.452 | PARK7 |
| 0.48 | 0.246 | NOP58 |
| 0.763 | 0.585 | HERPUD1 |
| 0.744 | 0.551 | NDUFA4 |
| 0.408 | 0.186 | PHLDA1 |
| 0.397 | 0.17 | AMBP |
| 0.41 | 0.193 | BTG3 |
| 0.592 | 0.359 | TANK |
| 0.287 | 0.105 | CD58 |
| 0.493 | 0.271 | TIMP1 |
| 0.385 | 0.174 | GLRX |
| 0.562 | 0.331 | CKLF |
| 0.407 | 0.193 | PHPT1 |
| 0.452 | 0.226 | NDUFC2 |
| 0.891 | 0.782 | HSP90AB1 |
| 0.676 | 0.396 | APOA2 |
| 0.931 | 0.772 | RGS1 |
| 0.324 | 0.137 | IFI6 |
| 0.334 | 0.144 | TYMP |
| 0.602 | 0.38 | GPX1 |
| 0.909 | 0.797 | SH3BGRL3 |
| 0.406 | 0.194 | IFI27L2 |
| 0.552 | 0.325 | NOP10 |
| 0.266 | 0.101 | CFAP20 |
| 0.68 | 0.48 | COX5B |
| 0.623 | 0.395 | UQCR10 |
| 0.81 | 0.643 | SUMO2 |
| 0.534 | 0.315 | NDUFV2 |
| 0.588 | 0.376 | UCP2 |
| 0.33 | 0.146 | HSBP1 |
| 0.722 | 0.526 | TCEB2 |
| 0.995 | 0.996 | TMSB10 |
| 0.294 | 0.123 | FAS |
| 0.844 | 0.627 | CD52 |
| 0.651 | 0.442 | COX8A |
| 0.531 | 0.321 | ERH |
| 0.285 | 0.119 | PRDX3 |
| 0.301 | 0.127 | CCL20 |
| 0.653 | 0.468 | NDUFA13 |
| 0.913 | 0.835 | UQCRB |
| 0.707 | 0.488 | S100A11 |
| 0.517 | 0.308 | SHFM1 |
| 0.615 | 0.41 | TMEM258 |
| 0.485 | 0.287 | PRDX5 |
| 0.329 | 0.156 | CNIH1 |
| 0.755 | 0.581 | UQCR11 |
| 0.769 | 0.619 | COX7A2 |
| 0.258 | 0.103 | CXCR3 |
| 0.43 | 0.233 | PFDN2 |
| 0.426 | 0.235 | USP15 |
| 0.279 | 0.119 | GALM |
| 0.392 | 0.203 | GSTO1 |
| 0.445 | 0.241 | CXCR6 |
| 0.324 | 0.152 | TMEM173 |
| 0.277 | 0.119 | PSMB5 |
| 0.688 | 0.507 | COX6A1 |
| 0.547 | 0.372 | APOE |
| 0.52 | 0.321 | CALM3 |
| 0.436 | 0.243 | CAPZA2 |
| 0.463 | 0.268 | LAPTM4A |
| 0.757 | 0.593 | UBL5 |
| 0.602 | 0.412 | CLEC2D |
| 0.57 | 0.378 | PSMB1 |
| 0.7 | 0.514 | TMEM59 |
| 0.316 | 0.151 | CISD2 |
| 0.467 | 0.285 | EIF3J |
| 0.284 | 0.129 | GBP5 |
| 0.483 | 0.293 | WDR83OS |
| 0.367 | 0.189 | CHMP2A |
| 0.716 | 0.526 | FXYD5 |
| 0.301 | 0.139 | VTN |
| 0.724 | 0.572 | MORF4L1 |
| 0.329 | 0.161 | ZFAND2A |
| 0.821 | 0.706 | SKP1 |
| 0.511 | 0.308 | ANXA2 |
| 0.341 | 0.171 | GTF3C6 |
| 0.566 | 0.383 | SPCS1 |
| 0.276 | 0.129 | NT5C3A |
| 0.272 | 0.125 | C4orf48 |
| 0.5 | 0.312 | CSTB |
| 0.264 | 0.117 | SIT1 |
| 0.282 | 0.13 | NDUFC1 |
| 0.746 | 0.615 | TRBC2 |
| 0.382 | 0.209 | DYNLRB1 |
| 0.445 | 0.262 | TMED9 |
| 0.456 | 0.269 | LAMTOR5 |
| 0.405 | 0.228 | ARPP19 |
| 0.308 | 0.154 | MRPL18 |
| 0.733 | 0.588 | COX6C |
| 0.425 | 0.251 | PBXIP1 |
| 0.428 | 0.252 | MRPL33 |
| 0.579 | 0.401 | VAMP8 |
| 0.758 | 0.639 | CLIC1 |
| 0.388 | 0.217 | SLIRP |
| 0.388 | 0.22 | OCIAD2 |
| 0.344 | 0.183 | MRPL51 |
| 0.277 | 0.131 | SAR1B |
| 0.459 | 0.285 | BST2 |
| 0.372 | 0.211 | AHSA1 |
| 0.306 | 0.153 | ATP5G1 |
| 0.321 | 0.166 | COX20 |
| 0.287 | 0.142 | ETFB |
| 0.476 | 0.301 | ATP5J |
| 0.291 | 0.144 | SEC11C |
| 0.685 | 0.526 | COX6B1 |
| 0.537 | 0.367 | NDUFB8 |
| 0.588 | 0.423 | SEC61G |
| 0.308 | 0.158 | NDUFB3 |
| 0.272 | 0.133 | HPRT1 |
| 0.362 | 0.201 | MPC2 |
| 0.324 | 0.175 | STIP1 |
| 0.271 | 0.132 | PFDN4 |
| 0.425 | 0.259 | NPC2 |
| 0.367 | 0.208 | RGS10 |
| 0.644 | 0.491 | ATP5I |
| 0.747 | 0.61 | CYTIP |
| 0.377 | 0.217 | ANXA5 |
| 0.548 | 0.381 | COPE |
| 0.492 | 0.321 | NDUFA11 |
| 0.485 | 0.312 | HSPA1B |
| 0.253 | 0.123 | PSMD14 |
| 0.63 | 0.474 | POMP |
| 0.376 | 0.218 | ANAPC11 |
| 0.284 | 0.145 | CLU |
| 0.446 | 0.284 | ROMO1 |
| 0.651 | 0.507 | ATP6V0E1 |
| 0.387 | 0.233 | PLP2 |
| 0.789 | 0.684 | HINT1 |
| 0.51 | 0.345 | NDUFB1 |
| 0.517 | 0.356 | SPOCK2 |
| 0.355 | 0.2 | RNF181 |
| 0.653 | 0.527 | HSPD1 |
| 0.543 | 0.387 | CFLAR |
| 0.304 | 0.162 | TXNDC17 |
| 0.637 | 0.482 | ARL6IP5 |
| 0.282 | 0.148 | SMS |
| 0.443 | 0.284 | RBX1 |
| 0.929 | 0.866 | RPS26 |
| 0.327 | 0.184 | PSMD4 |
| 0.271 | 0.14 | COPZ1 |
| 0.522 | 0.378 | RAP1A |
| 0.576 | 0.421 | EMP3 |
| 0.857 | 0.787 | ATP5L |
| 0.324 | 0.181 | DUSP10 |
| 0.585 | 0.432 | UQCRQ |
| 0.264 | 0.136 | FAM129A |
| 0.305 | 0.168 | PDCD10 |
| 0.45 | 0.297 | ECH1 |
| 0.345 | 0.197 | MRPS6 |
| 0.558 | 0.401 | NDUFB2 |
| 0.566 | 0.436 | PLIN2 |
| 0.507 | 0.353 | ATP5G3 |
| 0.789 | 0.693 | DNAJA1 |
| 0.547 | 0.376 | HSPH1 |
| 0.417 | 0.274 | RAB9A |
| 0.639 | 0.503 | USMG5 |
| 0.646 | 0.512 | ANAPC16 |
| 0.295 | 0.163 | MRPL52 |
| 0.32 | 0.183 | ZNHIT1 |
| 0.342 | 0.202 | HBP1 |
| 0.766 | 0.667 | ATP5G2 |
| 0.671 | 0.513 | BIRC3 |
| 0.484 | 0.329 | BSG |
| 0.323 | 0.191 | EPSTI1 |
| 0.381 | 0.236 | ATP6V0B |
| 0.411 | 0.263 | TMCO1 |
| 0.496 | 0.349 | ST13 |
| 0.536 | 0.392 | NEDD8 |
| 0.33 | 0.196 | AP2S1 |
| 0.279 | 0.154 | CCR6 |
| 0.373 | 0.23 | PSMB2 |
| 0.404 | 0.264 | PDIA6 |
| 0.508 | 0.372 | PSMA2 |
| 0.371 | 0.234 | NR3C1 |
| 0.327 | 0.196 | APOBEC3C |
| 0.53 | 0.386 | BRK1 |
| 0.304 | 0.179 | PSMD7 |
| 0.423 | 0.277 | ATP6V1F |
| 0.779 | 0.681 | UBE2D3 |
| 0.595 | 0.461 | PSME2 |
| 0.421 | 0.281 | ATP5C1 |
| 0.566 | 0.424 | CUTA |
| 0.421 | 0.278 | MINOS1 |
| 0.789 | 0.709 | CHCHD2 |
| 0.602 | 0.467 | NDUFA1 |
| 0.563 | 0.43 | SUMO1 |
| 0.264 | 0.146 | SNRPC |
| 0.503 | 0.348 | APOC1 |
| 0.477 | 0.345 | C9orf16 |
| 0.43 | 0.291 | PSMD8 |
| 0.287 | 0.164 | RPA3 |
| 0.423 | 0.28 | YWHAE |
| 0.43 | 0.291 | FAM96B |
| 0.285 | 0.165 | GTF2A2 |
| 0.282 | 0.162 | ZBTB38 |
| 0.381 | 0.247 | CHURC1 |
| 0.337 | 0.209 | SFT2D1 |
| 0.662 | 0.541 | PSMA7 |
| 0.275 | 0.158 | CHMP5 |
| 0.415 | 0.279 | RHOC |
| 0.308 | 0.185 | IL10RA |
| 0.271 | 0.156 | TMEM167A |
| 0.387 | 0.253 | BLOC1S1 |
| 0.498 | 0.366 | PSMB8 |
| 0.297 | 0.178 | NDUFB5 |
| 0.268 | 0.152 | HLA-DMA |
| 0.311 | 0.191 | MDH1 |
| 0.36 | 0.235 | SNRPE |
| 0.362 | 0.235 | C11orf31 |
| 0.334 | 0.207 | PDCL3 |
| 0.549 | 0.423 | C4orf3 |
| 0.384 | 0.255 | RAC1 |
| 0.355 | 0.224 | NDUFS6 |
| 0.688 | 0.586 | TMBIM6 |
| 0.512 | 0.381 | PRR13 |
| 0.452 | 0.321 | LAMTOR4 |
| 0.29 | 0.174 | EIF4E |
| 0.308 | 0.191 | ANXA7 |
| 0.43 | 0.298 | DOK2 |
| 0.47 | 0.341 | DAD1 |
| 0.452 | 0.329 | TRAPPC1 |
| 0.294 | 0.18 | BANF1 |
| 0.577 | 0.456 | C14orf2 |
| 0.555 | 0.433 | PAIP2 |
| 0.751 | 0.676 | HSPE1 |
| 0.312 | 0.195 | RAB11A |
| 0.44 | 0.317 | NDUFB4 |
| 0.307 | 0.198 | RANBP1 |
| 0.266 | 0.158 | PSMC1 |
| 0.396 | 0.269 | NUDC |
| 0.367 | 0.242 | MRPS21 |
| 0.356 | 0.235 | CCT3 |
| 0.898 | 0.865 | HSPA8 |
| 0.593 | 0.472 | TRMT112 |
| 0.424 | 0.299 | SF3B6 |
| 0.268 | 0.16 | TALDO1 |
| 0.3 | 0.191 | RNF145 |
| 0.399 | 0.28 | UBE2N |
| 0.259 | 0.156 | MRPL47 |
| 0.388 | 0.264 | AP2M1 |
| 0.42 | 0.292 | LMAN2 |
| 0.715 | 0.618 | ARPC3 |
| 0.321 | 0.206 | CCT6A |
| 0.302 | 0.192 | ANP32A |
| 0.353 | 0.238 | MDH2 |
| 0.3 | 0.19 | DGUOK |
| 0.284 | 0.178 | EIF6 |
| 0.591 | 0.477 | PSMB9 |
| 0.279 | 0.173 | C7orf73 |
| 0.441 | 0.319 | MYEOV2 |
| 0.419 | 0.3 | C12orf57 |
| 0.306 | 0.2 | CTNNB1 |
| 0.345 | 0.234 | CAST |
| 0.263 | 0.162 | NDUFAB1 |
| 0.564 | 0.463 | EIF3H |
| 0.379 | 0.269 | TSPO |
| 0.432 | 0.314 | EID1 |
| 0.414 | 0.301 | COX7A2L |
| 0.389 | 0.274 | PRDM1 |
| 0.536 | 0.423 | SELT |
| 0.493 | 0.38 | NDUFB11 |
| 0.327 | 0.219 | ADRM1 |
| 0.338 | 0.229 | LAT |
| 0.251 | 0.155 | LINC01420 |
| 0.508 | 0.397 | TMEM50A |
| 0.269 | 0.17 | POLR2J |
| 0.297 | 0.191 | MTHFD2 |
| 0.272 | 0.173 | NDUFAF3 |
| 0.315 | 0.21 | RAB1A |
| 0.363 | 0.251 | GNG5 |
| 0.467 | 0.347 | PET100 |
| 0.323 | 0.224 | NDUFB7 |
| 0.446 | 0.337 | PSMA1 |
| 0.357 | 0.249 | NDUFA2 |
| 0.313 | 0.213 | NDUFS7 |
| 0.6 | 0.509 | YWHAB |
| 0.355 | 0.249 | TMBIM4 |
| 0.899 | 0.877 | CD74 |
| 0.434 | 0.323 | EIF2S2 |
| 0.389 | 0.28 | NDUFA6 |
| 0.29 | 0.192 | MAT2B |
| 0.349 | 0.247 | SERPINB9 |
| 0.454 | 0.35 | ATP5A1 |
| 0.407 | 0.309 | CCT4 |
| 0.468 | 0.36 | BUB3 |
| 0.538 | 0.448 | GPX4 |
| 0.268 | 0.177 | MMADHC |
| 0.396 | 0.296 | STAT3 |
| 0.542 | 0.446 | LY6E |
| 0.327 | 0.232 | PPA1 |
| 0.367 | 0.269 | SRI |
| 0.3 | 0.207 | COX5A |
| 0.53 | 0.422 | ISG15 |
| 0.738 | 0.668 | DNAJB1 |
| 0.281 | 0.193 | MGST3 |
| 0.336 | 0.239 | ATP5H |
| 0.343 | 0.249 | CMTM6 |
| 0.285 | 0.2 | PIM2 |
| 0.548 | 0.454 | ITM2A |
| 0.333 | 0.246 | VAMP5 |
| 0.29 | 0.206 | KRT10 |
| 0.293 | 0.208 | FABP5 |
| 0.906 | 0.897 | HSP90AA1 |
| 0.779 | 0.803 | PFN1 |
| 0.308 | 0.234 | CCND2 |
| 0.507 | 0.485 | MIF |
| 0.335 | 0.273 | SLC3A2 |
| 0.268 | 0.21 | GNLY |
| 0.448 | 0.463 | TRBC1 |
| **Notes:** Pct.1, Marker gene expressed in single cluster cells; Pct.2, Marker gene expressed in total cells | | |
