# Supplementary material for: Heterogeneity induced GZMA-F2R communication inefficient impairs antitumor immunotherapy of PD-1 mAb through JAK2/STAT1 signal suppression in hepatocellular carcinoma
Source: Cell Death Dis. 2022 Mar 7;13(3):213. doi: 10.1038/s41419-022-04654-7 (PMC8901912; doi:10.1038/s41419-022-04654-7)
Supplement: Supplementary file 3 — Table S3 [file 41419_2022_4654_MOESM3_ESM.docx]

| Table S3: Marker genes in tumor and cytotoxic cells | | | | | | |
| --- | --- | --- | --- | --- | --- | --- |
| Genes | P_val | avg_logFC | Pct.1 | Pct.2 | P_val_adj | Cluster |
| KLRB1 | 0 | 0.863955 | 0.636 | 0.397 | 0 | 0 |
| CXCR4 | 0 | 0.561552 | 0.928 | 0.749 | 0 | 0 |
| CD3D | 2.91E-237 | 0.382997 | 0.677 | 0.469 | 5.40E-233 | 0 |
| PTPRC | 9.77E-233 | 0.345964 | 0.757 | 0.546 | 1.81E-228 | 0 |
| CD8A | 3.67E-199 | 0.571426 | 0.287 | 0.126 | 6.82E-195 | 0 |
| KLRD1 | 8.39E-153 | 0.345337 | 0.476 | 0.296 | 1.56E-148 | 0 |
| CCL3 | 2.16E-101 | 0.382719 | 0.452 | 0.347 | 4.01E-97 | 0 |
| CD44 | 6.75E-77 | 0.254742 | 0.514 | 0.406 | 1.25E-72 | 0 |
| EZR | 4.18E-62 | 0.259669 | 0.533 | 0.463 | 7.77E-58 | 0 |
| FCGR3A | 0 | 1.460371 | 0.579 | 0.126 | 0 | 1 |
| KLRD11 | 0 | 0.861865 | 0.797 | 0.367 | 0 | 1 |
| ANXA1 | 2.08E-92 | 0.35702 | 0.797 | 0.61 | 3.87E-88 | 1 |
| CD99 | 1.14E-55 | 0.336863 | 0.648 | 0.561 | 2.12E-51 | 1 |
| SPN | 6.30E-47 | 0.353177 | 0.291 | 0.176 | 1.17E-42 | 1 |
| CD37 | 7.35E-45 | 0.272734 | 0.564 | 0.432 | 1.37E-40 | 1 |
| ITGB1 | 1.36E-26 | 0.321985 | 0.441 | 0.37 | 2.52E-22 | 1 |
| CCL31 | 8.63E-08 | 0.463634 | 0.427 | 0.411 | 0.001603264 | 1 |
| IL7R | 0 | 0.983647 | 0.891 | 0.382 | 0 | 2 |
| CCR7 | 0 | 0.862573 | 0.352 | 0.078 | 0 | 2 |
| CCR6 | 0 | 0.755353 | 0.428 | 0.111 | 0 | 2 |
| ACTA2 | 6.73E-209 | 0.441078 | 0.262 | 0.064 | 1.25E-204 | 2 |
| KLF6 | 1.16E-182 | 0.628659 | 0.843 | 0.608 | 2.16E-178 | 2 |
| ANXA11 | 4.26E-169 | 0.606601 | 0.815 | 0.611 | 7.92E-165 | 2 |
| CD441 | 7.74E-159 | 0.484114 | 0.761 | 0.448 | 1.44E-154 | 2 |
| LYZ | 5.05E-75 | 0.305078 | 0.281 | 0.131 | 9.37E-71 | 2 |
| PBXIP1 | 7.94E-74 | 0.348322 | 0.412 | 0.224 | 1.48E-69 | 2 |
| EZR1 | 2.16E-59 | 0.317285 | 0.669 | 0.492 | 4.01E-55 | 2 |
| ABRACL | 1.18E-33 | 0.256882 | 0.476 | 0.34 | 2.19E-29 | 2 |
| TM4SF1 | 0 | 2.743133 | 0.946 | 0.114 | 0 | 3 |
| CD9 | 0 | 2.194803 | 0.913 | 0.088 | 0 | 3 |
| A2M | 0 | 1.957065 | 0.877 | 0.101 | 0 | 3 |
| PECAM1 | 0 | 1.707019 | 0.843 | 0.062 | 0 | 3 |
| ADAMTS1 | 0 | 1.603945 | 0.541 | 0.019 | 0 | 3 |
| VWF | 0 | 1.391248 | 0.552 | 0.009 | 0 | 3 |
| FLT1 | 0 | 1.387511 | 0.69 | 0.028 | 0 | 3 |
| ENG | 0 | 1.363561 | 0.672 | 0.047 | 0 | 3 |
| AKAP12 | 0 | 1.361571 | 0.472 | 0.048 | 0 | 3 |
| CD34 | 0 | 1.220245 | 0.632 | 0.006 | 0 | 3 |
| ADAM15 | 0 | 1.052059 | 0.559 | 0.027 | 0 | 3 |
| MYC | 0 | 1.049989 | 0.474 | 0.09 | 0 | 3 |
| CDH5 | 0 | 1.042873 | 0.611 | 0.018 | 0 | 3 |
| CTNNB1 | 0 | 0.98613 | 0.738 | 0.241 | 0 | 3 |
| IL6ST | 0 | 0.943034 | 0.712 | 0.145 | 0 | 3 |
| TSPAN7 | 0 | 0.87959 | 0.514 | 0.03 | 0 | 3 |
| ITGB11 | 0 | 0.830497 | 0.834 | 0.346 | 0 | 3 |
| ITGA6 | 0 | 0.823468 | 0.458 | 0.03 | 0 | 3 |
| ADAMTS4 | 0 | 0.816679 | 0.329 | 0.023 | 0 | 3 |
| LRRC32 | 0 | 0.76462 | 0.375 | 0.012 | 0 | 3 |
| IL3RA | 0 | 0.712503 | 0.413 | 0.005 | 0 | 3 |
| THY1 | 0 | 0.686283 | 0.282 | 0.006 | 0 | 3 |
| ITGA5 | 0 | 0.646677 | 0.466 | 0.055 | 0 | 3 |
| NRP1 | 0 | 0.641597 | 0.416 | 0.032 | 0 | 3 |
| ANXA5 | 0 | 0.619238 | 0.759 | 0.25 | 0 | 3 |
| NES | 0 | 0.610761 | 0.347 | 0.003 | 0 | 3 |
| ABCG2 | 0 | 0.605673 | 0.303 | 0.009 | 0 | 3 |
| ACVRL1 | 0 | 0.562885 | 0.359 | 0.005 | 0 | 3 |
| MCAM | 0 | 0.443481 | 0.288 | 0.024 | 0 | 3 |
| CPM | 0 | 0.437714 | 0.267 | 0.036 | 0 | 3 |
| CD40 | 0 | 0.430114 | 0.331 | 0.038 | 0 | 3 |
| ITGA1 | 0 | 0.368697 | 0.28 | 0.036 | 0 | 3 |
| LGALS3 | 2.31E-261 | 0.489423 | 0.498 | 0.14 | 4.28E-257 | 3 |
| ACTN1 | 3.17E-258 | 0.389452 | 0.279 | 0.052 | 5.89E-254 | 3 |
| ABCG1 | 5.52E-241 | 0.295174 | 0.258 | 0.046 | 1.03E-236 | 3 |
| ABI2 | 5.99E-234 | 0.290062 | 0.272 | 0.053 | 1.11E-229 | 3 |
| RTN4 | 1.60E-221 | 0.563707 | 0.712 | 0.295 | 2.98E-217 | 3 |
| CD46 | 4.47E-186 | 0.349146 | 0.465 | 0.15 | 8.29E-182 | 3 |
| ICAM1 | 5.62E-168 | 0.503243 | 0.413 | 0.135 | 1.04E-163 | 3 |
| FABP5 | 4.75E-147 | 0.758635 | 0.508 | 0.212 | 8.83E-143 | 3 |
| ACTA21 | 4.65E-140 | 0.639322 | 0.256 | 0.068 | 8.64E-136 | 3 |
| CD74 | 4.06E-102 | 0.494005 | 0.96 | 0.871 | 7.55E-98 | 3 |
| IL2RA | 2.84E-192 | 1.0819 | 0.262 | 0.036 | 5.28E-188 | 4 |
| B2M | 9.98E-131 | 0.369686 | 1 | 0.999 | 1.85E-126 | 4 |
| CD27 | 5.07E-117 | 1.01145 | 0.405 | 0.127 | 9.41E-113 | 4 |
| CD3D1 | 4.11E-51 | 0.571614 | 0.762 | 0.592 | 7.64E-47 | 4 |
| FABP51 | 3.74E-19 | 0.699378 | 0.339 | 0.227 | 6.94E-15 | 4 |
| LGALS31 | 4.58E-18 | 0.484242 | 0.263 | 0.16 | 8.50E-14 | 4 |
| PTPRC1 | 7.79E-17 | 0.302556 | 0.757 | 0.675 | 1.45E-12 | 4 |
| ANXA12 | 1.04E-14 | 0.25317 | 0.393 | 0.634 | 1.94E-10 | 4 |
| CD741 | 4.31E-13 | 0.317462 | 0.825 | 0.878 | 8.00E-09 | 4 |
| CD83 | 5.37E-13 | 0.562138 | 0.266 | 0.179 | 9.98E-09 | 4 |
| CD3G | 5.38E-13 | 0.372388 | 0.424 | 0.327 | 9.99E-09 | 4 |
| ADI1 | 0 | 1.404797 | 0.743 | 0.183 | 0 | 5 |
| TF | 0 | 1.116479 | 0.744 | 0.142 | 0 | 5 |
| A1BG | 0 | 1.106507 | 0.741 | 0.17 | 0 | 5 |
| SLC2A2 | 0 | 1.046496 | 0.56 | 0.056 | 0 | 5 |
| AADAC | 0 | 1.042042 | 0.593 | 0.062 | 0 | 5 |
| CYP17A1 | 0 | 0.928773 | 0.504 | 0.048 | 0 | 5 |
| KRT18 | 0 | 0.918948 | 0.679 | 0.119 | 0 | 5 |
| APOE | 2.53E-246 | 2.274105 | 0.857 | 0.441 | 4.70E-242 | 5 |
| CD24 | 2.44E-211 | 0.597822 | 0.363 | 0.059 | 4.53E-207 | 5 |
| ALDH1A1 | 2.77E-203 | 0.658892 | 0.501 | 0.113 | 5.14E-199 | 5 |
| CADM1 | 2.81E-132 | 0.368204 | 0.269 | 0.05 | 5.22E-128 | 5 |
| SDC1 | 3.20E-128 | 0.343664 | 0.262 | 0.049 | 5.95E-124 | 5 |
| ACAT2 | 5.43E-126 | 0.473458 | 0.391 | 0.099 | 1.01E-121 | 5 |
| ACADS | 7.98E-125 | 0.391255 | 0.281 | 0.057 | 1.48E-120 | 5 |
| CDC20 | 0 | 0.716936 | 0.352 | 0.011 | 0 | 6 |
| CCNA2 | 0 | 0.474141 | 0.255 | 0.005 | 0 | 6 |
| ANP32E | 3.29E-149 | 0.692404 | 0.656 | 0.207 | 6.11E-145 | 6 |
| ACAT21 | 1.56E-130 | 0.425914 | 0.412 | 0.099 | 2.91E-126 | 6 |
| HPRT1 | 1.07E-101 | 0.454089 | 0.489 | 0.153 | 1.99E-97 | 6 |
| ACTL6A | 2.78E-83 | 0.302493 | 0.261 | 0.063 | 5.17E-79 | 6 |
| ACTB | 2.92E-78 | 0.878653 | 0.998 | 0.989 | 5.42E-74 | 6 |
| SLC2A21 | 2.84E-74 | 0.446973 | 0.253 | 0.065 | 5.28E-70 | 6 |
| ADI11 | 4.60E-74 | 0.557253 | 0.489 | 0.191 | 8.55E-70 | 6 |
| PARP1 | 2.61E-67 | 0.34993 | 0.48 | 0.181 | 4.86E-63 | 6 |
| TF1 | 8.31E-65 | 0.689166 | 0.399 | 0.152 | 1.54E-60 | 6 |
| CD38 | 4.97E-60 | 0.385768 | 0.256 | 0.074 | 9.23E-56 | 6 |
| KRT181 | 1.60E-54 | 0.492034 | 0.344 | 0.129 | 2.97E-50 | 6 |
| FABP52 | 5.68E-42 | 0.386428 | 0.484 | 0.224 | 1.05E-37 | 6 |
| APOE1 | 5.39E-40 | 1.192941 | 0.662 | 0.447 | 1.00E-35 | 6 |
| A1BG1 | 4.79E-34 | 0.591099 | 0.363 | 0.181 | 8.90E-30 | 6 |
| ANXA52 | 7.81E-33 | 0.313009 | 0.527 | 0.275 | 1.45E-28 | 6 |
| AKAP121 | 0 | 2.539614 | 0.872 | 0.055 | 0 | 7 |
| CD14 | 0 | 2.519213 | 0.791 | 0.079 | 0 | 7 |
| TSPAN71 | 0 | 1.872714 | 0.8 | 0.042 | 0 | 7 |
| ENG1 | 0 | 1.842426 | 0.843 | 0.068 | 0 | 7 |
| ADAMTS41 | 0 | 1.645419 | 0.538 | 0.03 | 0 | 7 |
| CD4 | 0 | 1.62611 | 0.737 | 0.069 | 0 | 7 |
| IL6ST1 | 0 | 1.609591 | 0.827 | 0.165 | 0 | 7 |
| TM4SF11 | 0 | 1.393294 | 0.92 | 0.148 | 0 | 7 |
| FLT11 | 0 | 1.341958 | 0.665 | 0.055 | 0 | 7 |
| A2M1 | 0 | 1.213461 | 0.735 | 0.136 | 0 | 7 |
| NRP11 | 0 | 1.164928 | 0.569 | 0.044 | 0 | 7 |
| ECM1 | 0 | 1.020034 | 0.415 | 0.008 | 0 | 7 |
| CDH51 | 0 | 0.93252 | 0.501 | 0.044 | 0 | 7 |
| ANPEP | 1.76E-261 | 0.775141 | 0.357 | 0.044 | 3.27E-257 | 7 |
| ICAM11 | 4.27E-208 | 1.216149 | 0.581 | 0.142 | 7.93E-204 | 7 |
| LRRC321 | 3.49E-189 | 0.5521 | 0.255 | 0.03 | 6.48E-185 | 7 |
| MYC1 | 1.20E-161 | 1.092444 | 0.453 | 0.106 | 2.23E-157 | 7 |
| ITGA11 | 1.45E-139 | 0.511103 | 0.277 | 0.046 | 2.69E-135 | 7 |
| ACTN11 | 4.75E-135 | 0.581189 | 0.313 | 0.061 | 8.82E-131 | 7 |
| B4GALT5 | 1.53E-117 | 0.51307 | 0.315 | 0.065 | 2.85E-113 | 7 |
| PECAM11 | 9.51E-114 | 0.431055 | 0.422 | 0.104 | 1.77E-109 | 7 |
| ADAMTS11 | 1.23E-111 | 0.543539 | 0.258 | 0.047 | 2.28E-107 | 7 |
| ALDH1A11 | 1.44E-66 | 0.396656 | 0.357 | 0.118 | 2.67E-62 | 7 |
| ITGB12 | 4.78E-65 | 0.533728 | 0.713 | 0.368 | 8.88E-61 | 7 |
| CD91 | 1.16E-64 | 0.705002 | 0.376 | 0.134 | 2.16E-60 | 7 |
| ANXA53 | 3.72E-48 | 0.371594 | 0.578 | 0.274 | 6.91E-44 | 7 |
| RTN41 | 6.17E-46 | 0.450451 | 0.598 | 0.314 | 1.15E-41 | 7 |
| CD461 | 2.11E-42 | 0.354546 | 0.388 | 0.164 | 3.92E-38 | 7 |
| KLF61 | 7.27E-28 | 0.454538 | 0.819 | 0.622 | 1.35E-23 | 7 |
| CTNNB11 | 3.47E-18 | 0.301706 | 0.441 | 0.268 | 6.44E-14 | 7 |
| KRT182 | 0 | 1.995114 | 0.852 | 0.118 | 0 | 8 |
| ALDH1A12 | 0 | 1.708154 | 0.716 | 0.11 | 0 | 8 |
| UCHL1 | 0 | 1.556563 | 0.541 | 0.006 | 0 | 8 |
| MAGEA4 | 0 | 1.292723 | 0.535 | 0.002 | 0 | 8 |
| CD241 | 0 | 1.229032 | 0.477 | 0.058 | 0 | 8 |
| AFP | 0 | 1.09181 | 0.268 | 0.008 | 0 | 8 |
| TF2 | 1.09E-280 | 1.415755 | 0.693 | 0.146 | 2.03E-276 | 8 |
| ANPEP1 | 6.30E-185 | 0.551933 | 0.32 | 0.046 | 1.17E-180 | 8 |
| TM4SF12 | 1.07E-170 | 0.735904 | 0.608 | 0.156 | 1.99E-166 | 8 |
| ANXA54 | 1.21E-110 | 0.939164 | 0.665 | 0.273 | 2.24E-106 | 8 |
| NRP12 | 1.67E-104 | 0.327026 | 0.271 | 0.051 | 3.10E-100 | 8 |
| ACTL6A1 | 7.66E-79 | 0.35638 | 0.269 | 0.063 | 1.42E-74 | 8 |
| MYC2 | 4.90E-60 | 0.40123 | 0.338 | 0.109 | 9.11E-56 | 8 |
| FABP53 | 1.10E-44 | 0.54085 | 0.488 | 0.225 | 2.05E-40 | 8 |
| ACAT22 | 4.41E-37 | 0.351283 | 0.278 | 0.104 | 8.19E-33 | 8 |
| LGALS32 | 4.05E-26 | 0.331809 | 0.336 | 0.159 | 7.52E-22 | 8 |
| CYP2C9 | 3.64E-178 | 0.662047 | 0.328 | 0.024 | 6.76E-174 | 9 |
| IL7R1 | 9.60E-32 | 0.612897 | 0.787 | 0.419 | 1.78E-27 | 9 |
| ADI12 | 4.41E-27 | 0.296899 | 0.519 | 0.195 | 8.19E-23 | 9 |
| ACTB1 | 5.89E-27 | 0.483251 | 1 | 0.989 | 1.09E-22 | 9 |
| KLF62 | 3.76E-23 | 0.456606 | 0.932 | 0.624 | 6.98E-19 | 9 |
| CCR61 | 2.52E-17 | 0.265793 | 0.345 | 0.134 | 4.68E-13 | 9 |
| ABRACL1 | 2.93E-17 | 0.30788 | 0.685 | 0.348 | 5.45E-13 | 9 |
| FXYD2 | 0 | 3.263507 | 0.934 | 0.023 | 0 | 10 |
| KRT7 | 0 | 2.42329 | 0.829 | 0.01 | 0 | 10 |
| KRT19 | 0 | 2.146796 | 0.671 | 0.011 | 0 | 10 |
| EPCAM | 0 | 1.49365 | 0.697 | 0.01 | 0 | 10 |
| SOX9 | 0 | 1.020167 | 0.526 | 0.009 | 0 | 10 |
| ARL14 | 0 | 0.847769 | 0.329 | 0.003 | 0 | 10 |
| CDH1 | 6.06E-287 | 0.643953 | 0.408 | 0.008 | 1.13E-282 | 10 |
| CD242 | 6.25E-187 | 2.126938 | 0.868 | 0.065 | 1.16E-182 | 10 |
| CPM1 | 1.10E-171 | 1.353421 | 0.737 | 0.048 | 2.05E-167 | 10 |
| KRT183 | 1.21E-129 | 2.848187 | 0.974 | 0.132 | 2.25E-125 | 10 |
| ABCC3 | 1.81E-97 | 0.625508 | 0.408 | 0.025 | 3.36E-93 | 10 |
| TM4SF13 | 1.38E-86 | 1.79537 | 0.947 | 0.164 | 2.57E-82 | 10 |
| ANPEP2 | 2.78E-72 | 0.971623 | 0.5 | 0.05 | 5.17E-68 | 10 |
| CDH2 | 5.02E-51 | 0.537297 | 0.329 | 0.031 | 9.33E-47 | 10 |
| SDC11 | 3.25E-49 | 0.738762 | 0.434 | 0.054 | 6.03E-45 | 10 |
| ALCAM | 3.41E-47 | 0.554507 | 0.303 | 0.028 | 6.33E-43 | 10 |
| KLF63 | 8.86E-39 | 1.695049 | 0.961 | 0.626 | 1.65E-34 | 10 |
| SLC2A22 | 5.48E-35 | 0.782193 | 0.434 | 0.069 | 1.02E-30 | 10 |
| ALDH1A13 | 3.15E-33 | 0.724493 | 0.579 | 0.122 | 5.85E-29 | 10 |
| LGALS33 | 6.80E-32 | 0.895922 | 0.645 | 0.161 | 1.26E-27 | 10 |
| AQP3 | 6.24E-27 | 0.713978 | 0.434 | 0.086 | 1.16E-22 | 10 |
| ANXA55 | 7.14E-25 | 0.969234 | 0.763 | 0.28 | 1.33E-20 | 10 |
| LYZ1 | 2.76E-19 | 0.743958 | 0.5 | 0.142 | 5.13E-15 | 10 |
| CD92 | 1.35E-16 | 0.41178 | 0.487 | 0.139 | 2.51E-12 | 10 |
| ITGB13 | 2.87E-14 | 0.730251 | 0.763 | 0.375 | 5.34E-10 | 10 |
| ACTN12 | 1.45E-13 | 0.416025 | 0.276 | 0.066 | 2.69E-09 | 10 |
| CD462 | 7.93E-10 | 0.385916 | 0.447 | 0.169 | 1.47E-05 | 10 |
| ADI13 | 1.34E-09 | 0.481552 | 0.5 | 0.197 | 2.50E-05 | 10 |
| GLUL | 1.20E-08 | 0.489875 | 0.711 | 0.36 | 0.000222367 | 10 |
| RTN42 | 1.34E-08 | 0.511024 | 0.632 | 0.32 | 0.000248881 | 10 |
| MYC3 | 4.60E-07 | 0.433163 | 0.303 | 0.114 | 0.00854586 | 10 |
| KIT | 0 | 1.701304 | 0.733 | 0.003 | 0 | 11 |
| CPM2 | 2.29E-79 | 1.622807 | 0.567 | 0.049 | 4.25E-75 | 11 |
| CD94 | 3.55E-57 | 1.719364 | 0.8 | 0.139 | 6.59E-53 | 11 |
| GLUL1 | 3.45E-35 | 1.889252 | 0.917 | 0.36 | 6.40E-31 | 11 |
| LGALS34 | 3.31E-15 | 0.904531 | 0.517 | 0.162 | 6.15E-11 | 11 |
| ANXA14 | 3.52E-15 | 1.097474 | 0.95 | 0.627 | 6.54E-11 | 11 |
| ICAM12 | 9.11E-10 | 0.412967 | 0.45 | 0.152 | 1.69E-05 | 11 |
| CD831 | 2.59E-09 | 0.563742 | 0.467 | 0.18 | 4.82E-05 | 11 |
| ID21 | 3.38E-09 | 0.719709 | 0.9 | 0.716 | 6.27E-05 | 11 |
| CD442 | 1.43E-08 | 0.563491 | 0.833 | 0.472 | 0.000265103 | 11 |
| CD68 | 7.69E-08 | 0.638642 | 0.267 | 0.08 | 0.001428175 | 11 |
| BCL2A1 | 2.59E-07 | 0.781582 | 0.417 | 0.172 | 0.004817856 | 11 |
| LYZ2 | 1.70E-06 | 0.446057 | 0.35 | 0.142 | 0.031612603 | 11 |
| Notes: Pct.1, Marker gene expressed in single cluster cells; Pct.2, Marker gene expressed in total cells | | | | | | |
