# Supplementary material for: Heterogeneity induced GZMA-F2R communication inefficient impairs antitumor immunotherapy of PD-1 mAb through JAK2/STAT1 signal suppression in hepatocellular carcinoma
Source: Cell Death Dis. 2022 Mar 7;13(3):213. doi: 10.1038/s41419-022-04654-7 (PMC8901912; doi:10.1038/s41419-022-04654-7)
Supplement: Supplementary file 4 — Table S4 [file 41419_2022_4654_MOESM4_ESM.docx]

| Table S4. Clinicopathologic Characteristics of PDL-1 positive HCC patients | |
| --- | --- |
| Characteristics | No. of patients |
| Patients | 18 |
| Age, y |  |
| ≤50 | 8 |
| >50 | 10 |
| Sex |  |
| Male | 15 |
| Female | 3 |
| AFP, ng/mL |  |
| ≤20 | 5 |
| >20 | 13 |
| TNM staging |  |
| I | 9 |
| II | 7 |
| III | 2 |
| Tumor size, cm |  |
| ≤5 | 7 |
| >5 | 11 |
| Tumor number |  |
| Single | 14 |
| Multiple | 4 |
| Microvascular invasion |  |
| Yes | 10 |
| No | 8 |
| Macrovascular invasion |  |
| Yes | 3 |
| No | 15 |
| Encapsulation |  |
| Complete | 9 |
| None | 9 |
| Hepatitis B virus DNA |  |
| ≤1000 IU/mL | 12 |
| >1000 IU/mL | 6 |
| Cirrhosis |  |
| Yes | 14 |
| No | 4 |
| Differentiation |  |
| II | 3 |
| III | 14 |
| Ⅳ | 1 |
| BCLC |  |
| A | 10 |
| B | 3 |
| C | 5 |
| AFP, α-fetoprotein; BCLC, Barcelona Clinic Liver Cancer staging. | |
