# Supplementary material for: Heterogeneity induced GZMA-F2R communication inefficient impairs antitumor immunotherapy of PD-1 mAb through JAK2/STAT1 signal suppression in hepatocellular carcinoma
Source: Cell Death Dis. 2022 Mar 7;13(3):213. doi: 10.1038/s41419-022-04654-7 (PMC8901912; doi:10.1038/s41419-022-04654-7)
Supplement: Supplementary file 5 — Table S5 [file 41419_2022_4654_MOESM5_ESM.docx]

| Table S5. Primers used and shRNA for F2R and GZMA knockdown | | |
| --- | --- | --- |
| Primers |  |  |
| Gene Symbol | Forward Primer | Reverse Primer |
| Human F2R | Gaaaccttcctgctgagcct | Cagcgcataatgggtgcttc |
| Human GZMA | Agtgcatcttggtccgatactc | Gcttccagaatctccattgcac |
| Human GAPDH | Gagcctcaagatcatcagca | Tgtggtcatgagtccttcca |
| shRNA sequences |  |  |
| Target Gene | Sense | Antisense |
| Human F2R-shRNA | Aaggctactatgcctactact | Agtagtaggcatagtagcctt |
| Human GZMA-shRNA | Guguugacugcagcucacu | Agugagcugcagucaacac |
