# Supplementary material for: Heterogeneity induced GZMA-F2R communication inefficient impairs antitumor immunotherapy of PD-1 mAb through JAK2/STAT1 signal suppression in hepatocellular carcinoma
Source: Cell Death Dis. 2022 Mar 7;13(3):213. doi: 10.1038/s41419-022-04654-7 (PMC8901912; doi:10.1038/s41419-022-04654-7)
Supplement: Supplementary file 6 — Table S6 [file 41419_2022_4654_MOESM6_ESM.docx]

| Table S6. Clinicopathologic Characteristics of HCC patients | |
| --- | --- |
| Characteristics | No. of patients |
| Patients | 158 |
| Age, y |  |
| ≤50 | 79 |
| >50 | 77 |
| Sex |  |
| Male | 156 |
| Female | 2 |
| AFP, ng/mL |  |
| ≤20 | 62 |
| >20 | 96 |
| TNM staging |  |
| I | 84 |
| II | 66 |
| III | 8 |
| Tumor size, cm |  |
| ≤5 | 53 |
| >5 | 105 |
| Tumor number |  |
| Single | 129 |
| Multiple | 29 |
| Microvascular invasion |  |
| Yes | 93 |
| No | 65 |
| Macrovascular invasion |  |
| Yes | 20 |
| No | 138 |
| Encapsulation |  |
| Complete | 85 |
| None | 73 |
| Hepatitis B virus DNA |  |
| ≤1000 IU/mL | 115 |
| >1000 IU/mL | 43 |
| Cirrhosis |  |
| Yes | 130 |
| No | 28 |
| Differentiation |  |
| II | 20 |
| III | 127 |
| Ⅳ | 11 |
| BCLC |  |
| A | 93 |
| B | 29 |
| C | 36 |
| AFP, α-fetoprotein; BCLC, Barcelona Clinic Liver Cancer staging. | |
