# Supplementary material for: Heterogeneity induced GZMA-F2R communication inefficient impairs antitumor immunotherapy of PD-1 mAb through JAK2/STAT1 signal suppression in hepatocellular carcinoma
Source: Cell Death Dis. 2022 Mar 7;13(3):213. doi: 10.1038/s41419-022-04654-7 (PMC8901912; doi:10.1038/s41419-022-04654-7)
Supplement: Supplementary file 7 — Table S7 [file 41419_2022_4654_MOESM7_ESM.docx]

| Table S7. Regents and antibody colonies used in this article | | |
| --- | --- | --- |
| Regents | Source | Identifier |
| Granzyme A Polyclonal Antibody | Proteintech | 11288-1-AP |
| PAR1 Monoclonal Antibody (ATAP2) | Thermo fisher | 35-2200 |
| JAK2 Antibody - AF6022 | Affinity | AF6022 |
| Phospho-JAK2 (Tyr931) Antibody | Affinity | AF3024 |
| STAT1 Polyclonal Antibody | Proteintech | 10144-2-AP |
| Active Caspase-3 Rabbit mAb | Abclonal | A11021 |
| FITC-conjugated Goat Anti-Rabbit IgG | Elabscience | E-AB-1014 |
| FITC-conjugated Goat Anti-Mouse IgG | Elabscience | E-AB-1015 |
| TRITC-conjugated Goat Anti- Rabbit IgG | Elabscience | E-AB-1053 |
| HRP-conjugated Goat Anti-Mouse IgG | Elabscience | E-AB-1008 |
| HRP-conjugated Goat Anti-Rabbit IgG | Elabscience | E-AB-1003 |
| Alpha Tubulin Polyclonal Antibody | Proteintech | 11224-1-AP |
| GAPDH Monoclonal Antibody | Proteintech | 60004-1-Ig |
| AMMS®Anti-human CD3/CD28 beads | T&L Biological | GMP-TL601 |
| Sintilimab | Innovent | DP2104008 |
| Recombinant Human IL-2 | Abcam | ab179500 |
| One Step TUNEL Apoptosis Assay Kit | Beyotime | C1088 |
| Cell Counting Kit-8 | Beyotime | C0038 |
| Crystal Violet Staining Solution | Beyotime | C0121 |
| Nuclear Extraction Kit | Abcam | ab113474 |
| Lipofectamine™ 3000 Transfection Reagent | Invitrogen | L3000001 |
| Fetal Bovine Serum | Gibco | 10100147 |
| Dulbecco's Modified Eagle Medium | Gibco | 12100046 |
| RPMI 1640 Medium | Gibco | 31800022 |
| Lymphocyte Separation Medium (Human) | Solarbio | P8610 |
| Red Blood Cell Lysis Buffer | Solarbio | R1010 |
| DMSO (Dimethyl sulfoxide) | Solarbio | D8371 |
| RIPA buffer(high) | Solarbio | R0010 |
| Penicillin-Streptomycin Liquid | Solarbio | P1400 |
| Trypsin-EDTA solution,0.25% | Solarbio | T1320 |
