# Supplementary material for: Heterogeneity induced GZMA-F2R communication inefficient impairs antitumor immunotherapy of PD-1 mAb through JAK2/STAT1 signal suppression in hepatocellular carcinoma
Source: Cell Death Dis. 2022 Mar 7;13(3):213. doi: 10.1038/s41419-022-04654-7 (PMC8901912; doi:10.1038/s41419-022-04654-7)
Supplement: Supplementary file 8 — Revised manuscript-highlights [file 41419_2022_4654_MOESM8_ESM.docx]

Heterogeneity induced GZMA-F2R communication inefficient impairs antitumor immunotherapy of PD-1 mAb through JAK2/STAT1 signal suppression in hepatocellular carcinoma

Yuxue Gao^1^, Qingguo Xu^2^, Xinqiang Li^2^, Yuan Guo^2^, Bowen Zhang^3^, Yan Jin^2^, Cunle zhu^2^, Yuntai Shen^2^, Pengxiang Yang^1^, Ying Shi^1^, Rifeng Jin^4^, Daojie Liu^5^, Yabo Ouyang^1^, Xiaoni Liu^1^, Wenjing Wang^1^, Dexi Chen^1, 2^, Tongwang Yang ^1, 2^

*^1^Beijing Institute of Hepatology, Capital Medical University, Beijing, 100069, China*

*^2^**Organ Transplantation Center,* *The Affiliated Hospital of* *Qingdao University, Qingdao City, 266003 China*

*^3^Department of Pathology, Inner Mongolia Baogang Hospital, Baotou, 014010, China.*

*^4^School of Chemical, Biological and Environmental Engineering, Carnegie Mellon University, Corvallis, 97331 USA*

*^5^Department of Clinical Laboratory, Haidian Maternal&Child Health Hospital, Beijing, 100080, China.*

*Tongwang Yang: No.59, Haier Road,Laoshan District, Qingdao, China, Phone: +8617853293276, Email:* [*1203620677@qq.com*](mailto:1203620677@qq.com)

The authors declare that they have no conflict of interest.

**Abstract**

Tumor heterogeneity has been associated with immunotherapy and targeted drug resistance in hepatocellular carcinoma (HCC). However, communications between tumor and cytotoxic cells are poorly understood to date. In the present study, thirty-one clusters of cells were discovered in the tumor tissues and adjacent tissues through single-cell sequencing. Moreover, the quantity and function exhaustion of cytotoxic cells was observed to be induced in tumors by the TCR and apoptosis signal pathways. Furthermore, granzyme failure of cytotoxic cells was observed in HCC patients. Importantly, the GZMA secreted by cytotoxic cells was demonstrated to interact with the F2R expressed by the tumor cells both *in vivo and in vitro*. This interaction induced tumor suppression and T cell-mediated killing of tumor cells via the activation of the JAK2/STAT1 signaling pathway. Mechanistically, the activation of JAK2/STAT1 signaling promoted apoptosis under the mediating effect of the LDPRSFLL motif at the N-terminus of F2R, which interacted with GZMA. In addition, GZMA and F2R were positively correlated with PD-1 and PD-L1 in tumor tissues, while the expressions of F2R and GZMA promoted PD-1 mAb-induced tumor suppression in both mouse model and HCC patients. Finally, in HCC patients, a low expression of GZMA and F2R in the tumor tissues was correlated with aggressive clinicopathological characteristics and poor prognosis. Collectively, GZMA-F2R communication inefficient induces deficient PD-1 mAb therapy and provide a completely novel immunotherapy strategy for tumor suppression in HCC patients.

## Introduction

T cells are strictly controlled via the presentation of ligands and their co-stimulatory and co-inhibitory receptors for the maintenance of self-tolerance during tumor suppression [1]. Immune checkpoints are widely recognized for their role in tumor suppression based on the outstanding outcomes reported for PD-1 and PD-L1 mAb [2-5]. Unfortunately, only a limited number of patients have demonstrated long-term responses to the therapies based on immune checkpoints [6, 7].

Studies have suggested a heterogeneous character of tumors [8, 9], with several differences existing among different types of primary tumors and also between primary tumors and metastatic tumors [10]. In addition, polyclonal tumors might develop over time [11]. Highly heterogeneous tumors exhibit different genotypes among patients or at different sites within a single patient [12, 13]. Molecular pathology provides directs guidelines for the application of immune checkpoint blockades. However, such examinations have failed to reveal all the molecular information regarding tumor heterogeneity [14, 15]. Therefore, it is of great significance to reveal the characteristics of various immune cells and the communications between the immune cells and tumor cells as this information would assist in reshaping the tumor microenvironment during antitumor immunotherapy.

F2R is the most extensively studied member of the PAR family [16, 17]. The 425 amino acids present at the N-terminal domain of F2R are recognized and proteolytically cleaved by specific ligands [18]. The activation of F2R, which depends on the type and the concentration of the acting ligands, then promotes platelet activation, cell proliferation, apoptosis, and angiogenesis [19-21]. GZMA is reported to regulate immune defense by inducing apoptosis, pyrosis, and maintaining homeostasis through the killing of the bacteria and parasites invading the host cells [22, 23]. Interestingly, GZMA has been reported to competitively interact with F2R against thrombin, although the GZMA-F2R binding has not been demonstrated to induce the coagulation process that is usually induced by the thrombin-F2R interaction [24]. Thus, it is necessary to understand the molecular mechanism underlying the GZMA/F2R communication in cancer patients.

Here, we revealed the following: (i) the quantity and function exhaustion of cytotoxic cells in the tumor tissues of HCC patients; (ii) the failure of the GZMA-F2R communication in tumor tissues; (iii) the induction of caspase3-dependent apoptosis by the LDPRSFLL motif-activated GZMA–F2R communication via the promotion of the JAK2/STAT1 signaling pathway; (iv) synergistic effect of GZMA-F2R communication and PD-1 mAb therapy in both mouse model and HCC patients; (v) the correlation of the low expression of GZMA/F2R with aggressive clinicopathological characteristics and poor prognosis in HCC patients. The above findings suggested that failure of the GZMA-F2R communication in the tumor tissues of HCC patients limited the antitumor immunotherapy based on immune checkpoint blockades. Therefore, the present study would contribute to and highlighting a completely different direction for scientific research on therapeutic strategy designing in HCC.

**Results**

### Cytotoxic cell exhaustion in the tumor tissues of HCC patients

To well understand HCC, 40195 high-quality, thirty-one subpopulation cells were screened out from a total of 55632 cells using the following conditions: nFeature_RNA > 500 & nCount_RNA > 1 000 & nCount_RNA < 20 000 & percent.mt < 15 (Fig. S1A). The top 5 marker genes in subpopulations were visualized on a heatmap (Fig. S1B). The annotate of subpopulations was performed using Human Cell Atlas Data (Table S1). Although a little batch effect was observed (Fig. S1C), the cells from different sources and samples were generally not distributed evenly in subpopulations (Fig. S1D). These results suggested that the high heterogeneity of HCC patients may be the appropriate reason that generic anti-hepatocellular carcinoma drugs were challenging to be developed [25].

Cytotoxic cells are essential for HCC suppression [26-28]. Unexpectedly, abundant of cytotoxic cells were observed in the adjacent tissues (Fig. 1Aa-c and Fig. 1B), while the T-cells exhaustion markers LAYN and CTLA4 were overexpressed in the tumor tissues (Fig. 1C-D). Subsequently, Kyoto Encyclopedia of Genes analysis and Genomes and Gene Ontology analysis were performed for the marker genes of the cytotoxic cells (Table S2). The analyses revealed the enrichment of the apoptotic and T-cell receptor signaling pathways (Fig. S2A). Importantly, in terms of number and function depletion, the gene profile of the cellular components had been reprogrammed, while the molecular function, responses to extracellular factors, and the biological process had been significantly altered (Fig. S2B-E). These results suggested cytotoxic cells exhaustion in the tumor tissues in terms of T-cell receptor and apoptosis activation.

### Granzymes failure of cytotoxic cells in the tumor tissue from HCC patients

Although cytotoxic cell exhaustion in tumor tissues was revealed in the present study, the underlying molecular mechanism was little understood. Here, the cytotoxic cells were isolated (Fig. 2A), and the top 5 marker genes in subpopulation were visualized in a heatmap (Fig. 2B). Interestingly, wide expression of granzymes (granzyme A, B, M, K, and H) was detected in all cytotoxic cells (Fig. 2C, Fig. S3Aa, S3Ba, S3Ca, and S3Da). Unfortunately, a massive number of granzyme-negative cytotoxic cells were observed in the tumor tissues (Fig. 2E, Fig. S3Ac, S3Bc, S3Cc, and S3Dc). In addition, the cytotoxic cells and granzyme-positive cytotoxic cells were significantly decreased in the tumor tissues (Fig. 2F, Fig. S3Ad, S3Bd, S3Cd, and S3Dd). According to these results, it was inferred that the granzyme-negative cytotoxic cells in the tumor tissues could be the appropriate reason why the cytotoxic cells failed to lyse the tumor cells.

### GZMA-F2R communication failure in the tumor tissues from HCC patients

In order to further elucidate the mechanism underlying the failure of cytotoxic cells to induce tumor suppression, the cytotoxic cells and tumor cells were separated (Fig. 3A), and subsequently the cell-cell communication was performed using CellChat R package. Interestingly, fifty-five outgoing communications in five patterns were revealed (Fig. S4A-B). Importantly, the PAR signal secreted by cytotoxic cells was mainly received by the tumor cells (Fig. 3B-D and Table S3), and the GZMA-F2R contributed to the primary PAR communication signaling pathway network (Fig. 3E). Furthermore, GZMA was co-localized with F2R in HepG2 co-cultured with CD3^+^ T-cells and tumor tissues (Fig. 3F). Unfortunately, the GZMA-positive cytotoxic cells were detected mainly in the adjacent tissues (Fig. S4C), while the tumor cells from the tumor tissues (cluster 5 and 8) were negative for F2R (Fig. 3G). Together, these results suggested the failure of GZMA-F2R communication in the tumor tissues.

### GZMA-F2R communication-mediated tumor cell killing

The above results demonstrated that there was a failure of GZMA-F2R communication in HCC patients. However, the functional role of GZMA-F2R communication remained poorly understood so far in the study. Interestingly, high levels of natural killer cells, activated CD4 T cells, natural killer T cells, and activated CD8 T cells, were observed in high GZMA/F2R expression HCC patients (Fig. 4A). Accordingly, T-cell-mediated tumor cell killing and CCK8 assays were performed in the HepG2 and Huh7 cells infected with F2R-sh-Lv, EGFP-sh-Lv, F2R-Lv, and EGFP-Lv in a co-culture with the CD3^+^ T cells infected with GZMA-rAd, RFP-rAd, GZMA-sh-rAd, and RFP-sh-rAd, respectively. As depicted in Fig. 4B and 4C, F2R-Lv infection promoted tumor suppression in the CD3^+^ T-cells infected with GZMA-rAd, while the reverse of this was observed in the cells infected with F2R-sh-Lv and GZMA-sh-rAd.

Induction of apoptosis is an essential function of T cells in tumor suppression [29, 30]. In this context, immunoblotting was performed for activated caspase3 was evaluation. Lower level of activated caspase3 were detected in the F2R-sh-Lv-infected Huh7 cells co-cultured with RFP-sh-rAd-infected CD3^+^ T cells (Fig. 4Da), while increased levels of activated caspase3 were observed in the F2R-Lv-infected Huh7 cells cultured with GZMA-rAd-infected CD3^+^ T cells (Fig. 4Db). Importantly, Calcein AM/PI double staining further confirmed the apoptosis-inducing property of F2R and GZMA expression (Fig. 4E). Together, these results suggested that the GZMA-F2R communication induced T cell-mediated tumor cell killing that relied on caspase3 activation.

### GZMA-F2R communication promoted JAK2/STAT2 signaling pathway

In order to decipher the molecular mechanism underlying the GZMA-F2R communication in tumor suppression, the differentially expressed genes in the low F2R/GZMA expression HCC patients were identified (Fig. S5). Moreover, suppression of the IL6/JAK2/STAT and IL2/STAT signals was revealed in the GSEA analysis (Fig. 5A). Therefore, p-JAK2 was quantified through western blotting in the above cell co-culture system. Interestingly, the levels of p-JAK2 were increased in the F2R-Lv-infected Huh7 cells co-cultured with the GZMA-rAd-infected CD3^+^ T cells (Fig. 5Bb). On the contrary, the reverse of the above phenomenon was observed in the F2R-sh-Lv-infected Huh7 cells co-cultured with the GZMA-sh-rAd-infected CD3^+^ T cells (Fig. 5Ba). Furthermore, western blotting followed by a nuclear extraction revealed the nuclear translocation of STAT1 in the F2R-Lv-infected Huh7 cells co-cultured with the GAMA-rAd-infected CD3^+^ T cells, while the reverse of this was observed for the F2R-sh-Lv-infected Huh7 cells co-cultured with the GZMA-sh-rAd-infected T cells (Fig. 5C). The finding of the nuclear translocation was further confirmed in the results of the immunofluorescence assay (Fig. 5D). Together, these results suggested that the GZMA-F2R communication suppressed the tumor by promoting the activation of JAK2/STAT1 signaling pathway.

### LDPRSFLL motif in the F2R-activated GZMA-F2R communication

In order to completely understand the mechanism underlying the GZMA-F2R communication, LDPRSFLL-deleted and LDPRSFLL-mutated motifs (Fig. S6) were generated using PCR. As depicted in Fig. 6A-6B, increased levels of activated caspase3 and p-JAK2 were detected in the F2R-rAd-infected Huh7 cells co-cultured with CD3^+^ T cells, and these levels were decreased in the F2R-rAd-infected cells containing the LDPRSFLL-mutated or LDPRSFLL-deleted motifs and the cells incubated with the LDPRSFLL motif-specific inhibitors SCH530348 and SCH79797. Importantly, the T cell-mediated tumor cell killing assay and Calcein-AM/PI double staining confirmed the promotion of F2R-induced tumor suppression by the LDPRSFLL motif (Fig. 6C-6D). Consistently, western blotting followed by a nuclear extraction revealed the nuclear translocation of STAT1 in the F2R-rAd-infected Huh7 cells co-cultured with CD3^+^ T cells, while the reverse of this was observed in the F2R-rAd-infected cells containing the LDPRSFLL-mutated or LDPRSFLL-deleted motif and the cells incubated with SCH530348 or SCH79797 (Fig. 6E). The nuclear translocation of STAT1 was further confirmed in the immunofluorescence assay (Fig. 6F). Together, these results demonstrated that the GZMA-F2R communication-induced tumor suppression might be promoted by the LDPRSFLL motif.

### Low expression of GZMA and F2R impair the therapeutic efficacy of PD-1 mAb in both mouse model and HCC patients

The above result demonstrated that the GZMA-F2R communication promotes cytotoxicity in tumor suppression. It is widely accepted that PD-1 and PD-L1 expressions impair antitumor therapy [31, 32]. In this context, the relationship among GZMA, F2R, PD-1, and PD-L1 in TCGA, LIHC, and GTEx was analyzed in the present study. The results revealed that GZMA and F2R were positively correlated with PD-1 and PD-L1 in cancers and tissues (Fig. S7A-S7C). Therefore, to fund out the role of F2R in the tumor suppression of PD-1 mAb therapy, Hepa1-6 cell, 5*10^5^, infected with F2R-Lv or EGFP-Lv, were subcutaneously injected into immune-competent *C57BL/6* mice that had been previously injected with PD-1 mAb or IgG2a (Fig. 7A). Interestingly, decreased tumor growth (Fig. 7B and Fig. S8A) and increased levels of activated caspase3 and apoptotic cells (Fig. S9A) were observed in the F2R-Lv-infected and PD-1 mAb-injected mice.

In order to further understand the role of the GZMA-F2R communication in the F2R-promoting effects of PD-1 mAb in tumor suppression, 5*10^5^ Huh7 cells-infected with F2R-sh-Lv or EGFP-sh-Lv were subcutaneously injected into immune-deficient *BALB/c* nude mice. Subsequently, 1*10^6^ CD3^+^ T cells-infected with GZMA-sh-rAd or RFP-sh-rAd and PD-1 mAb were injected into the mice to rebuild the immune system (Fig. 7C). Increased tumor growth (Fig. 7C and Fig. S8B) and decreased levels of activated caspase3 and apoptotic cells (Fig. S9B) were observed in the F2R-sh-Lv and GZMA-sh-rAd-infected and IgG mAb treated mice. Unfortunately, no significant change in tumor growth was observed in the F2R-sh-Lv-infected mice. Interestingly, increased tumor growth and decreased levels of caspase3 activation and apoptosis cells were observed in GZMA-sh-rAd infected mice (Fig. 7C-7D and Fig. S8B and S9B). These results demonstrated that low expression of F2R and GZMA resulted in poor ability of PD-1 mAb for tumor suppression.

In order to confirm the above findings in cancer patients, 18 PD-L1-positive HCC patients injected with PD-1 mAb, comprising ten responders, and eight non-responders (Table S4), were recruited for the analysis of GZMA and F2R expressions. Two representative cases of tumor diameter change (indicated with a red line) in response to PD-1 mAb therapy were are depicted in Fig. 7E, while two representative cases of GZMA and F2R are depicted in Fig. 7F. Importantly, low expressions of GZMA and F2R were observed in the PD-1 mAb non-responder patients (Fig. 7G). In addition, the expressions of GZMA and F2R were positively correlated with the changed diameter (Fig. 7H and 7I). Together, these results suggested that the tumor suppression property of PD-1 mAb was impaired in the case of GZMA-F2R communication failure.

### Down regulation of GZMA and F2R was associated with aggressive clinicopathological characteristics and a poor prognosis in HCC patients

In order to better understand the role of GZMA-F2R communication in HCC, the GZMA and F2R RNA-seq data from 33 cancer types and 9 HCC datasets were analyzed. The analysis revealed a down regulation of F2R in the tumor tissues of CESC, KICH, KIRP, LUAD, LUSC, and UCEC, while a low expression of GZMA was observed in COAD, LUAD, LUSC, UCEC, PAAD, and READ (Fig. S10A). In the HCC RNA-seq data from GEO, F2R expression was observed to be significantly downregulated in 5 datasets, while the GZMA expression was significantly downregulated in all datasets (Fig. S10B). Consistent with this result, the downregulation F2R and GZMA in the tumor tissue of HCC patients was also observed in the western blotting, quantitative real-time polymerase chain reaction (Fig. 8A and 8B; Table S5 and S6), and tissue microarray (Fig. 8C) analyses. These results confirmed that F2R and GZMA were downregulated in the tumor tissues.

In order to completely understand the contribution of F2R and GZMA, the correlation of the down-regulation of F2R and GZMA with sex, vascular infiltration, and encapsulation was evaluated (Fig. S11A). The low expression of F2R/GZMA was observed to be correlated with poor overall survival (median OS times:526 *vs.* 673 days; *P=0.034*), shorter time to disease-free survival (median DSS times: 526 *vs.* 673 days; *P=0.016*), and worse progression-free survival (median PFS times:363 *vs.* 524 days; *P=0.022*) (Fig. S10C). Similarly, low expression of the F2R/GZMA protein was correlated with poor overall survival (median OS times:27.35 *vs.* 49.25 months; *P=0.027*) and shorter time to recurrence (median TTR times: 31.15 *vs.* 43.00 months; *P=0.015*) in HCC patients (Fig. 8D). Furthermore, the multivariate analysis revealed that F2R and GZMA, together with microvascular invasion, and AFP, are independent risk factors for both OS and TTR (Fig. S11B). The low F2R-expression patients exhibited a higher risk for tumor recurrence (TTR: HR=0.974; 95% confidence interval: 0.951–0.997; *P=0.029*), while the low GZMA expression patients exhibited a shorter OS rate and a higher risk for tumor recurrence (OS: HR=0.973; 95% confidential interval: 0.955–0.992; *P=0.007*; TTR: HR=0.973; 95% confidence interval: 0.955–0.991; *P=0.004*). According to these, it was inferred that the frequently downregulated F2R and GZMA correlated with aggressive clinicopathological characteristics and a poor prognosis in HCC

**Discussion**

Tumor progression is closely associated with the antitumor activity of the immune system [33]. In tumors, T cells exhaustion in terms of highly expressed immunosuppressive receptors and reduced secretion of functional cytokines is reported [34], which induces T cell inactivation and inefficiency during the antitumor process, thereby resulting in immune escape [35, 36]. In the present study, it was revealed that the cytotoxic cells in tumor tissues are simultaneously exhausted in both quantity and function in terms of activation of TCR and apoptotic signals (Fig. 1B-1C and Fig. S2A-2B). Meanwhile, a decrease in the secreted granzyme and the proportion of granzyme positive cells was observed in the tumor tissues (Fig. 2 and Fig. S3). Therefore, a widely accepted essential strategy for tumor immunotherapy is to reverse the immune response of exhaustive T cells via the blocking of immune checkpoints [37].

In practice, antitumor therapy uses PD-1 mAb to neutralize the immunosuppressive receptor PD-1 [38], while PD-L1 mAb is used for neutralizing PD-L1 [39, 40]. Unfortunately, an increased number of patients failed to exhibit a long-term response to PD-1 mAb or PD-L1 mAb immunotherapy for heterogeneous immune and tumor cells [41]. In the present study, the GZMA-F2R communication promoted JAK2/STAT1 signal-induced tumor suppression both *in vitro and in vivo* (Fig. 4, 5, and 7). Unfortunately, two tumor cells exhibited negative expression of F2R in tumor tissues (Fig. 3D). Moreover, the low expression of GZMA and F2R was positively correlated with PD-1 and PD-L1 (Fig. S7), impaired tumor suppression by PD-1 mAb (Fig. 7, Fig. S8, Fig. 9), and also predicted aggressive clinicopathological characteristics and a poor prognosis (Fig. 8, Fig. S10, and Fig. 11). Therefore, the therapy strategies based on GZMA-F2R communication would be extremely potent in reversing the inefficient antitumor immunotherapy based on PD-1 mAb and PD-L1 mAb.

However, the present study revealed that the expressions of GZMA and F2R were positively correlated with PD-1 and PD-L1 (Fig. S7) and regulated the tumor suppression in PD-1 mAb therapy in both mouse models and HCC patients (Fig. 7). Moreover, the molecular mechanism underlying the GZMA-F2R communication in antitumor immunotherapy and its role in PD-1-suppressed tumor progression were elucidated (Fig. 3-8). However, to elucidate the specific molecular mechanisms underlying the expressions of PD-1 and PD-L1 in the GZMA-F2R communication failure, further investigation is warranted.

Thrombin is the most extensively elucidated ligand that interacts with F2R and promotes tumor progression [42-44]. However, multiple principal protease cleavage sites, thrombin, cathepsin, proteinase3, and human leukocyte elastase have been characterized at the N-terminal of F2R [24, 45]. In addition, F2R is reported to perform its biological functions in a ligand and concentration-dependent manner [46]. However, GZMA and thrombin recognize the same domain, named LDPRSFLL, although the interaction of thrombin with F2R induces platelet aggregation while the interaction of GZMA with F2R fails to induce platelet aggregation [47]. In the present study, the GZMA-F2R communication at the LDPRSFLL motif of F2R suppressed tumor progression by promoting the JAK2/STAT1 signal activation-induced apoptosis (Fig. 4-6). Interestingly, tumor volume was promoted in low GZMA expression mice while such promotion did not occur in the low F2R expression mice (Fig. 7 and Fig. S8). These results implicated that GMZA might be suppressing tumors via F2R and another factor. Assuredly, perforin delivers GZMA to the cytosol of target cell and suppresses tumor progression in caspase-independent apoptosis [48, 49].

The failure of the GZMA–F2R communication was identified in the tumor tissues of HCC patients. It was revealed that the binding of GZMA to the LDPRSFLL motif at the N-terminus of F2R promotes apoptosis via JAK2/STAT1 signaling, which in synergy with the PD-1 mAb therapy led to tumor suppression in both mouse model and HCC patients (Fig. S12). Therefore, a combination therapy comprising the modulation of GZMA-F2R communication and the use of an anti-PD-1 antibody would exhibit much better antitumor efficacy in the treatment of HCC patients.

Materials and Methods

### Data Collection

Single-cell sequencing matrices (GSE149614) and nine RNA-Seq datasets (GSE14520, GSE36376, GSE46444, GSE54236, GSE57957, GSE64041, GSE76297, GSE10207, and GSE121248) were download directly from the GEO. The RNA-seq data for 33 types of tumors were downloaded from the Genomic Data Commons Data Portal.

### Patients and Animals

A total of 158 pairs of tumor and adjacent tissue from HCC patients, for use in tissue microarray staining and total RNA or protein isolation, were collected from the Affiliated Hospital of Qingdao University, between March 2014 and August 2017 (Table S6). In addition, paraffin sections of 18 HCC patients who underwent PD-1 mAb treatment at the Affiliated Hospital of Qingdao University, between May 2017 and November 2020 (Table S4) were collected. Peripheral blood for PBMCs and T cell isolation was collected from healthy donors. The patients and their families were thoroughly informed regarding the study, and their approval was obtained in a written informed consent form that was signed by each participant. The present study was conducted under the guidelines and principles of the Declaration of Helsinki.

Six-week-old male *C57BL/6* or *BALB/c* nude mice were procured from SPF (Beijing) Biotechnology Co. Ltd. The *BALB/c* nude mice were then randomly distributed in individually ventilated cage (IVC) systems, while the *C57BL/6* mice were housed in specific pathogen-free (SPF) grade animal rooms. All animal experiments were conducted under guidelines of the Animal Care Facility of Qingdao University and those of the National Institute of Health. The study protocols were approved by the ethics committee of the Affiliated Hospital of Qingdao University (QYFYWZLL26539).

### Cell culture

HepG2, Huh7, Hepa1-6, and HEK-293A cell lines were purchased and authorized (STR profiling) from the China Center for Type Culture Collection. The cell lines were cultured in DMEM or MEM supplemented with 10% fetal bovine, 100 μg/mL streptomycin, and 100 U/mL penicillin. T cells were cultured in the RPMI-1640 supplemented with Recombinant Human IL-2, 10% fetal bovine, 100 μg/mL streptomycin, and 100 U/mL penicillin.

### Separation of PBMCs

Peripheral blood mononuclear cells (PBMCs) were separated as described in a previous report [50]. Briefly, peripheral blood was collected in a 15-ml tube and centrifuged at 2 000 rpm. The cell pellet was collected diluted, and mixed gently with 1x PBS. Subsequently, the lymphocyte separation medium was added to the PBMCs separation tube, followed by the addition of blood cells on the top of the lymphocyte separation medium. After 10 min of centrifugation at 2000 rpm, the separated cells were collected and washed twice with 1x PBS. Subsequently, the red blood cell lysis buffer was added to lyse the red blood cells, followed by two washes with 1x PBS.

### Separation of CD3^+^ T cell

The CD3^+^ T cells were separated as described in a previous report [51]. The CD3/CD28 conjugated magnetic beads were vortexed, and then, 100μl of the bead mixture was transferred to an EP tube and resuspended in PBS, while the supernatant was discarded. The resuspended beads were then mixed with the separated cells and incubated inside a shaker incubator for 30 min. Afterward, the mixture was equilibrated on a magnetic stand for 2 min. Finally, the mixture of cells and beads was resuspended and cultured for 9-14 days.

### Construction of vectors

Adenovirus was produced via the double transfection of HEK-293A cells with the adenoviral backbone vector (pDC316-GZMA-shRNA for the construction of shRNA-GZMA and pDC316-mCMV-EGFP for the construction of F2R-rAd, F2R_30-426_-rAd, F2R_44-426_-rAd, and F2R_S42A_-rAd) and the packaging plasmid pBHGlox-E1,3Cre in a ratio of 1:1 using Lipofectamine 3 000 (Invitrogen, CA, USA). A lentiviral transfer vector (pLV-CMV-shRNA for the construction of shRNA-F2R and pLV-F2R vector for construction of Lv-F2R) and two packaging plasmids pH1 and pH2 were triple transfected in a ratio of 0.5:0.35:0.15 into the HEK-293T cells for Lentivirus construction. The target sequences used for F2R and GZMA gene interference are provided in Table S5.

### *In vivo* tumor model

In order to establish the immune-competent mouse model, 4×10^6^ Hepa1-6 cells were mixed with Matrigel and then inoculated into *C57BL/6* mice for the establishment of an allograft HCC mouse model. In order to establish the immunodeficient mouse model, 2×10^6^ Huh7 cells were inoculated into BABL/c nude mice for developing the xenograft HCC mouse model. CD3^+^ T cells were injected into the mice via the tail vein to reconstitute the human immune system. PD1 mAb (Sintilimab, 10 μg/kg) or an IgG was injected into the mice via the tail vein once a week. Tumor diameter was recorded weekly. Later, the animal was sacrificed, a frozen tissue section was constructed, and the TUNEL assay and activated caspase3 staining were performed.

Immunoblotting

Tumor tissues or cell pellets were homogenized using cold lysis buffer procured from Solarbio Life Sciences. The homogenates were centrifuged at 8 000g for 30min. After BCA quantification, the supernatant was separated using SDS-PAGE gels, and the separated proteins were transferred to PVDF membranes. The membranes with the proteins were incubated separately with GZMA, F2R, JAK2, p-JAK2, STAT1, Tubulin, and GAPDH antibodies (1:1 000 dilution). After the incubation, the membranes were washed with 1xTBST and then incubated with the HRP-conjugated secondary antibodies (1:2 000 dilution). Afterward, the membrane was washed again with 1xTBST and then incubated with 1ml of the electrochemiluminescence solution. The protein bands were visualized using the Tanon image system. The regents and antibody clones that were used are listed in Table S7.

Nuclear extraction

The nuclear extraction assay was performed according to the protocol provided by the manufacturer [52]. Briefly, cells were collected in a 15-ml tube and centrifuged at 400g. The obtained cell pellet was collected and resuspended in 100 µL of the pre-extraction buffer. After 10 min, the cytoplasmic extract obtained was transferred to a fresh EP tube and then centrifuged at 8 000g. The resulting nuclear pellet was mixed with an extraction buffer, and the mixture was incubated on ice for 15 min. Subsequently, the mixture was sonicated in an ultrasonic disintegrator for 3x10 seconds. Afterward, the sonicated mixture was centrifuged a 12 000g for 10 min, and the nuclear extracts obtained were transferred to a fresh EP tube. Finally, immunoblotting was performed as described earlier.

Cell counting kit-8 assays

The cck8 assay was performed according to the protocol provided by the manufacturer [53]. Briefly, 2 000 cells were plated in a 96-well tissue culture plate for 8 h and then co-cultured with 8 000 (1:4) CD3^+^ T cells. At the end of the assay, the cells were incubated with 100μl of a medium supplemented with 10μl of the CCK-8 solution for 1 h. The 450nm absorbance was recorded at 450 nm using a microplate reader.

T cell-mediated tumor cell killing assay

The T cell-mediated tumor cell killing assay was performed as described in the report by of Hong L [54]. Briefly, the HepG2 and Huh7 cells were plated in a 96-well tissue culture plate for 8h and then co-cultured with CD3^+^ T cells for 48h. After removing the T cells and other cell debris, the remaining cells were stained with crystal violet and then quantified at 570 nm in a spectrometer.

### TUNEL assay

The terminal deoxynucleotidyl transferase dUTP nick-end labeling assay was performed as described in a previous report [55]. Briefly, the tissue slices were fixed using 70% alcohol and penetrated with 0.5% Triton X-100. The DNA 3'-OH in the apoptotic cells was linked with the FITC-labeled dUTP. The DNA was stained with DAPI, and the FITC-positive cell were visualized under a fluorescence microscope.

Calcein-AM/PI double staining assay

HepG2 and Huh7 cells were plated in a 24-well tissue culture plate and then co-cultured with CD3^+^ T cells for 48h. Subsequently, the staining of apoptotic cells was performed through incubation with propidium iodide at 37℃ for 15 min. Next, the living cells were stained using Calcein AM. The PI and Calcein AM-positive cells were visualized using fluorescence microscopy.

Fluorescence confocal assay

The cultured cells or tissue slices were fixed using 75% alcohol and penetrated with 0.5% Triton X-100. This was followed by incubation with GZMA, F2R, STAT1, and activated-casp3. Afterward, the samples were washed with 1xPBS and then incubated with FITC or TRITC-labeled secondary antibody (1:400 dilution). Subsequently, after another wash with 1x PBS, the samples were stained with DAPI and covered with a cover glass. Finally, the protein was visualized and photographed under a fluorescence microscope.

### Total RNA extraction

Tissues were homogenized using the TRIzol reagent. The lysed tissue homogenate was centrifuged at 3 000g, and the pellet was discarded. The obtained supernatant was incubated with chloroform and followed by thorough mixing and then centrifuged at 12 000g. The supernatant was collected, and isopropanol was added to it for RNA precipitation. The precipitate containing the RNA was again centrifuged at 12 000g. 70% ethanol was used to wash the RNA and collected at 8 000g. The precipitated RNA was finally dissolved in RNase-free water.

### cDNA synthesis

The cDNA was synthesized using the SuperScript® III First-Strand Synthesis kit for RT-qPCR. Total RNA (≤2.5 µg), dNTP mix (1 mM), and random hexamer primers (5 ng/µl) were mixed, followed by the addition of water to attain a final volume of 5 µl. RT buffer, RNaseOUT (2 U), DDT (10 mM), SuperScript^®^ III (10 U), and MgCl_2_ (5 mM) were mixed, forming a total volume of 10 µl. Subsequently, a standard cDNA synthesis program was run, and the synthesized cDNA was stored at -20˚C.

### qRT-PCR analysis

The primers, Sybr Green, and the synthesized cDNA were mixed and briefly centrifuged. Next 3.8 μl of ddH_2_O, 1 μl of cDNA, 5 μl of Sybr Green, and 0.2 μl of primer (10 μM) were added to a 384-well PCR plate. The PCR program conditions used were: denaturation at 95˚C for 15 s, annealing at 56˚C for 30 s, elongation at 72˚C for 50 s; the number of cycles run was 40. The housekeeping gene GAPDH was used as the internal standard. The primer pairs used are provided in Table S5.

Data and statistical analyses

The high-quality cells were separated by applying the following criteria: nFeature_RNA > 500 & nCount_RNA > 1000 & nCount_RNA < 20000 & percent.mt < 15. The FindClusters function was run to identify the cell clusters at a resolution of 0.5. The Human Cell Atlas Data (<http://biocc.hrbmu.edu.cn/CellMarker/>) was employed for the annotation of the identified cell clusters based on marker genes. RNA-Seq of tumor tissues and adjacent tissues data were downloaded from TCGA. The GSEA pathway analysis was performed based on the marker gene.

Statistical analyses were performed using IBM SPSS statistic 22 (IBM, New York, USA). The differences among the variables were determined using the two-tailed student’s t-test. The log-rank test was used for determining the progression-free, disease-free, overall, and disease interval survival. A stepwise Cox multivariate proportional hazard regression model was used for the multivariate analysis. Statistical significance was demonstrated by the *P-values* of *0.05*. The data were presented as mean ± SD.

## References

1. Saleh R, Toor SM, Nair VS, Elkord E. Role of Epigenetic Modifications in Inhibitory Immune Checkpoints in Cancer Development and Progression. Front Immunol 11, 1469 (2020).

2. Sui H, Ma N, Wang Y, Hui L, Liu X, Su Y, et al. Anti-PD-1/PD-L1 Therapy for Non-Small-Cell Lung Cancer: Toward Personalized Medicine and Combination Strategies. J Immunol Res 2018, 1-17 (2018).

3. Hugo W, Zaretsky JM, Sun L, Song CY, Moreno BH, Hu-Lieskovan S, et al. Genomic and Transcriptomic Features of Response to Anti-PD-1 Therapy in Metastatic Melanoma. Cell 168, 542 (2017).

4. Voorwerk L, Slagter M, Horlings HM, Sikorska K, van de Vijver KK, de Maaker M, et al. Immune induction strategies in metastatic triple-negative breast cancer to enhance the sensitivity to PD-1 blockade: the TONIC trial. Nat Med 25, 920-928 (2019).

5. Voutsadakis IA. International PD: PD-1 inhibitors monotherapy in hepatocellular carcinoma: Meta-analysis and systematic review. Hepatobiliary Pancreat Dis Int 18, 505-510 (2019).

6. Skoulidis F, Goldberg ME, Greenawalt DE, Hellmann MD, Awad MM, Gainor JF, et al. STK11/LKB1 mutations and PD-1 inhibitor resistance in KRAS-mutant lung adenocarcinoma. Cancer Discov 8, 822-835 (2018).

7. Galarreta D, Bresnahan E, Molina-Sánchez P, Lindblad KE, Barbara Maier B, Sia D, et al. Barbara: beta-Catenin Activation Promotes Immune Escape and Resistance to Anti-PD-1 Therapy in Hepatocellular Carcinoma. Cancer Discov 9, 1124-1141 (2019).

8. Hao JJ, Lin D, Dinh HQ, Mayakonda A, Jiang YY, Chang C, et al. Spatial intratumoral heterogeneity and temporal clonal evolution in esophageal squamous cell carcinoma. Nat Genet 48, 1500-1507 (2016).

9. Du K, Wei SY, Wei Z, Frederick DT, Miao BC, Moll T, et al. Pathway signatures derived from on-treatment tumor specimens predict response to anti-PD1 blockade in metastatic melanoma. Nat Commun 12, 6023(2021).

10. Kenichi S, Jihye K, Isao M, Leslie R, Masaki S, Shigeki S, et al. Innate Genetic Evolution of Lung Cancers and Spatial Heterogeneity: Analysis of Treatment-Nave Lesions. J Thorac Oncol 13, 1496-1507 (2018).

11. Chamseddine IM, Rejniak KA. Hybrid modeling frameworks of tumor development and treatment. Wiley Interdiscip Rev Syst Biol Med 12, e1461 (2020).

12. Vitale I, Manic G, Coussens LM, Kroemer G, Galluzzi LJ. Macrophages and Metabolism in the Tumor Microenvironment. Cell Metab 30, 36-50 (2019).

13. Liu T, Zhou L, Li D, Andl T, Zhang Y. Cancer-Associated Fibroblasts Build and Secure the Tumor Microenvironment. Front Cell Dev Biol 7, 60 (2019).

14. Eigentler TK, Hassel JC, Berking C, Aberle J, Bachmann O, Grünwald V, et al. Diagnosis, monitoring and management of immune-related adverse drug reactions of anti-PD-1 antibody therapy. Cancer Treat Rev 45, 7-18 (2016).

15. Schneider BJ, Naidoo J, Santomasso BD, Lacchetti C, Adkins S, Anadkat M, et al. Management of Immune-Related Adverse Events in Patients Treated With Immune Checkpoint Inhibitor Therapy: ASCO Guideline Update. J Clin Oncol 39, 4073-4126(2021).

16. Gutierrez-Rodriguez M, Herranz R. From Multiple PAR1 Receptor/Protein Interactions to their Multiple Therapeutic Implications. Curr Top Med Chem 15, 2080-2114 (2015).

17. Pompili E, Franchis VD, Giampietri C, Leone S, Santis ED, Fornai F, et al Protease Activated Receptor 1 and Its Ligands as Main Regulators of the Regeneration of Peripheral Nerves. Biomolecules 11, 1668(2021).

18. Han X, Nieman MT, Kerlin BA. Protease-activated receptors: An illustrated review. Res Pract Thromb Haemost 5, 17-26(2020).

19. Chang YH, Wu JC, Yu HM, Hsu HT, Wu YT, Yu ALT, et al. Design and synthesis of glyco-peptides as anti-cancer agents targeting thrombin-protease activated receptor-1 interaction. Chem Commun (Camb) 56, 5827-5830(2020).

20. Ray T, Pal A. PAR-1 mediated apoptosis of breast cancer cells by V. cholerae hemagglutinin protease. Apoptosis 21, 609-620(2016).

21. Wojtukiewicz MZ, Hempel D, Sierko E, Tucker SC, Honn KV. Endothelial Protein C Receptor (EPCR), Protease Activated Receptor-1 (PAR-1) and Their Interplay in Cancer Growth and Metastatic Dissemination. Cancers (Basel) 11, 51(2019).

22. Zeglinski MR, Granville DJ. Granzymes in cardiovascular injury and disease. Cell Signal 76, 109804 (2020).

23. Trapani JA. Granzymes, cytotoxic granules and cell death: the early work of Dr. Jurg Tschopp. Cell Death Differ 19, 21-7(2012).

24. Déry O, Corvera CU, Steinhoff M, Bunnett NW. Proteinase-activated receptors: novel mechanisms of signaling by serine proteases. Am J Physiol 274, 1429-1452 (1998).

25. Cabral L, Tiribelli C, Sukowati C. Sorafenib Resistance in Hepatocellular Carcinoma: The Relevance of Genetic Heterogeneity. Cancers (Basel) 12, 1576 (2020).

26. Chiu KC, Yuen WH, Cheu WS, Wei LL, Ting V, Fehlings M,et al. Hepatocellular Carcinoma Cells Up-regulate PVRL1, Stabilizing PVR and Inhibiting the Cytotoxic T-Cell Response via TIGIT to Mediate Tumor Resistance to PD1 Inhibitors in Mice. Gastroenterology 159, 609-623 (2020).

27. Zhang S, Zeng C, Wang D, Gao X, Guo S, Zhang Y, et al. Efficient induction of cytotoxic T lymphocytes in hepatocellular carcinoma using the HLA-A2-restricted survivin peptide in vitro. Exp Cell Res 386, 111741 (2020).

28. Meng FZ, Zhen SM, Song B. HBV-specific CD4+ cytotoxic T cells in hepatocellular carcinoma are less cytolytic toward tumor cells and suppress CD8+ T cell-mediated antitumor immunity. APMIS 125, 743-751 (2017).

29. Wesch D, Kabelitz D, Oberg HH. Tumor resistance mechanisms and their consequences on γδ T cell activation. Immunol Rev 298, 84-98 (2020).

30. Mel SD, Hue SS, Jeyasekharan AD, Chng WJ, Ng SB. Molecular pathogenic pathways in extranodal NK/T cell lymphoma. J Hematol Oncol 12, 33 (2019).

31. Dermani FK, Samadi P, Rahmani G, Kohlan AK, Najafi R. PD-1/PD-L1 immune checkpoint: Potential target for cancer therapy. J Cell Physiol 234, 1313-1325 (2019).

32. Ai L, Chen J, Yan H, He QJ, Luo PH, Xu ZF, et al. Research Status and Outlook of PD-1/PD-L1 Inhibitors for Cancer Therapy. Drug Des Devel Ther 14, 3625-3649 (2020).

33. Griggio V, Perutelli F, Salvetti C, Boccellato E, Boccadoro M, Vitale C, et al. Immune Dysfunctions and Immune-Based Therapeutic Interventions in Chronic Lymphocytic Leukemia. Front Immunol 11, 594556 (2020).

34. Melaiu O, Lucarini V, Giovannoni R, Fruci D, Gemignani F. News on immune checkpoint inhibitors as immunotherapy strategies in adult and pediatric solid tumors. Semin Cancer Biol S1044-579X, 30156-5 (2020).

35. Qin S, Xu L, Yi M, Yu S, Wu K, Luo S. Novel immune checkpoint targets: Moving beyond PD-1 and CTLA-4. Mol Cancer 18, 155 (2019).

36. Mehdizadeh S, Bayatipoor H, Pashangzadeh S, Jafarpour R, Shojaei Z, Motallebnezhad M. Immune checkpoints and cancer development: Therapeutic implications and future directions. Pathol Res Pract 223, 153485 (2021).

37. Pérez-Ruiz E, Melero I, Kopecka J, Sarmento-Ribeiro AB, Marilina García-Aranda M, Rivas JDL. Cancer immunotherapy resistance based on immune checkpoints inhibitors: Targets, biomarkers, and remedies. Drug Resist Updat 53, 100718 (2020).

38. Lei Q, Wang D, Sun K, Wang L, Zhang Y. Resistance Mechanisms of Anti-PD1/PDL1 Therapy in Solid Tumors. Front Cell Dev Biol 8, 672 (2020).

39. Sun C, Mezzadra R, Schumacher TN. Regulation and Function of the PD-L1 Checkpoint. Immunity 48, 434-452 (2018).

40. Makuku R, Khalili N, Razi S, Keshavarz-Fathi M, Rezaei N. Current and Future Perspectives of PD-1/PDL-1 Blockade in Cancer Immunotherapy. J Immunol Res 2021, 1-15 (2021).

41. Wang ZY, Wu XY. Study and analysis of antitumor resistance mechanism of PD1/PD-L1 immune checkpoint blocker. Cancer Med 9, 8086-8121 (2020).

42. Schweickert PG, Yang Y, White EE, Cresswell GM, Elzey BD, Ratliff TL. Thrombin‐PAR1 signaling in pancreatic cancer promotes an immunosuppressive microenvironment. J Thromb Haemost 19, 161-172 (2021).

43. Zigler M, Kamiya T, Brantley EC, Villares GJ, Bar-Eli M. PAR-1 and thrombin: the ties that bind the microenvironment to melanoma metastasis. Cancer Res 71, 6561-6566 (2011).

44. Heider I, Schulze B, Oswald E, Henklein P, Scheele J, Kaufmann R. PAR1-type thrombin receptor stimulates migration and matrix adhesion of human colon carcinoma cells by a PKCepsilon-dependent mechanism. Oncol Res 14, 475-482 (2004).

45. Willis Fox O, Preston RJ. Molecular basis of protease-activated receptor 1 signaling diversity. J Thromb Haemost 18, 6-16 (2020).

46. Posma JJ, Grover SP, Hisada Y, Owens AP 3rd, Antoniak S, Spronk HM, et al. Roles of Coagulation Proteases and PARs (Protease-Activated Receptors) in Mouse Models of Inflammatory Diseases. Arterioscler Thromb Vasc Biol 39, 13-24 (2019).

47. Suidan HS, Clemetson KJ, Brown-Luedi M, Niclou SP, Clemetson JM, Tschopp J. The serine protease granzyme A does not induce platelet aggregation but inhibits responses triggered by thrombin. Biochem J 315, 939-945 (1996).

48. Arias M, Martínez-Lostao L, Santiago L, Ferrandez A, Granville DJ, Pardo J. The Untold Story of Granzymes in Oncoimmunology: Novel Opportunities with Old Acquaintances. Trends Cancer 3, 407-422 (2017).

49. Wong PS, Sutejo R, Chen H, Ng SH, Sugrue RJ, Tan BH. A System Based-Approach to Examine Cytokine Response in Poxvirus-Infected Macrophages. Viruses 10, 692(2018).

50. Yang TW, Ouyang YB, Gao YX, Liu DJ, Zang YJ, Chen DX. Enriched highthroughput reverse transcriptionquantitative PCR template preparation without preamplification. Mol Med Rep 22, 3541-3548 (2020).

51. Wang XC, He QF, Shen HY, Xia AL, Tian WF, Yu WW, et al. TOX promotes the exhaustion of antitumor CD8+ T cells by preventing PD1 degradation in hepatocellular carcinoma. J Hepatol 71, 731-741 (2019).

52. Li LC, Zhao S, Liu ZF, Zhang NZ, Pang S, Liu JK, et al. Sunitinib treatment promotes metastasis of drug-resistant renal cell carcinoma via TFE3 signaling pathway. Cell Death Dis 12, 220 (2021).

53. Yu WH, Chen K, Ye GW, Wang S, Wang P, Li JT, et al. SNP-adjacent super enhancer network mediates enhanced osteogenic differentiation of MSCs in ankylosing spondylitis. Hum Mol Genet 30, 277-293 (2021).

54. Liu H, Kuang XW, Zhang YC, Ye YQ, Li JL, Liang L, et al. ADORA1 Inhibition Promotes Tumor Immune Evasion by Regulating the ATF3-PD-L1 Axis. Cancer Cell 37, 324-339 (2020).

55. Yang TW, Gao YX, Liu DJ, Wang Y, Wu J, Liu XN, et al. ASPP2 enhances chemotherapeutic sensitivity through the down-regulation of XIAP expression in a p53 independent manner in hepatocellular carcinoma. Biochem Biophys Res Commun 508, 769-774 (2019).

## Acknowledgments

We are grateful to the organizers and participants of the TCGA and GTEx project, and Lu Y, Beijing Institute of Radiation Medicine, for the single-cell RNA-Seq data available in GEO, which were used in the present study.

## Ethics

Samples were obtained with informed consent.

## Competing Interests

The authors declare that they have no conflict of interest.

## Author Contributions

Yang TW, Gao YX, Xu QG, Li XQ, Guo Y, Zhang BW, Jin Y, Zhu CL, Shen YT, Yang PX, and Jin RF contributed to data research. Yang TW, Xu QG, Shi Y, Liu DJ, Liu XN, and Cheng DX contributed designing the study. Yang TW, Guo Y, Gao YX, Xu QG, Shi Y, Liu XN, Ouyang YB, Wang WJ, Xu QG, and Jin RF assisted in report preparation. Yang TW and Chen DX supervised the study. All authors have thoroughly read and approved the final manuscript.

## Funding

The present research work was supported by the National Natural Science Foundation of China (82073676, 81470098, 81900575), the Beijing Municipal Commission of Science and Technology (Z191100006619064), the Beijing Municipal Natural Science Foundation, and the Beijing Municipal Education Commission (KZ202010025037).

## Data Availability Statement

The datasets used and/or analyzed in the present study are available with the corresponding author and would be provided for study upon reasonable request.

## Figure legends

Figure 1. Single-cell RNA-seq analysis of the cytotoxic cells in the tumor tissue of HCC patients. (A) The t-SNE map depicting the cytotoxic cells (red) in the (a), tumor tissues (b), adjacent tissues, and (c) tissues from eight HCC patients. (B) Bar plot depicting the ratio of cytotoxic cells in the tumor tissues and adjacent tissues for each HCC patient. (C) Expression t-SNE maps for the T cell exhaustion markers LAYN and CTLA4 in the tumor tissues and adjacent tissues.

Figure 2. Single-cell RNA-seq analysis of the granzyme failure in the cytotoxic cells from the tumor tissues of HCC patients. (A) The t-SNE map depicting clusters of cytotoxic cells in the tumor tissues and adjacent tissues of eight HCC patients. (B) Expression heatmap of the marker genes in each cytotoxic cell cluster (top 5, color-coded according to the cluster and expression). (C) Expression t-SNE maps for GZMA in the cytotoxic cells of the tumor tissues and adjacent tissues. (D) Expression t-SNE maps for GZMA in the cytotoxic cells from the tumor tissues. (E) Expression t-SNE maps for GZMA in the cytotoxic cells from the adjacent tissues. (F) Bar plot illustrating the ratio of GZMA positive cytotoxic cells in the tumor tissues and adjacent tissues for each HCC patient.

Figure 3. Cellular communication in cytotoxic cells and tumor cells. (A) The t-SNE map depicting clusters of cytotoxic cells and tumor cells in the tumor tissues and adjacent tissues of eight HCC patients. (B) The t-SNE map depicting the types of cytotoxic cells and tumor cells in the tumor and adjacent tissues, tumor tissues, and adjacent tissues of HCC patients, respectively. (C) Network depicting the PAR signaling pathway in cytotoxic cells and tumor cells. Color indicates the significance of clusters in the PAR signaling pathway. (D) Circle plot illustrating the PAR signaling pathway in cytotoxic cells and tumor cells. The color and line indicate the significance of clusters in the PARs signaling pathway. (E) Bar plot illustrating the contribution of ligands and receptors in the PAR signaling pathway in cytotoxic cells and tumor cells. (F) Fluorescence confocal assay results demonstrating the co-localization of F2R (green) and GZMA (red) in cultured cells (left) and tissues of an HCC patient (right). The nuclear DNA was stained with DAPI (blue). Scale bars: 15 μm. (G) Expression t-SNE maps for F2R in the tumor tissues and adjacent tissues, tumor tissues, and adjacent tissues of eight HCC patients.

Figure 4. Functional role of GZMA and F2R expression in tumor suppression. (A). Correlation of GZMA and F2R expression with the infiltrated immune cells in 372 LIHC from TCGA. (B) The T cell-mediated tumor cell killing assay performed in a 96-well plate and a 24-well plate. a: HepG2 and Huh7 cells, infected with EGFP-Lv or F2R-Lv, and co-cultured with the CD3^+^ T cells infected with GZMA-rAd or RFP-rAd, respectively. b: HepG2 and Huh7 cells, infected with EGFP-sh-Lv or F2R-sh-Lv, and co-cultured with the CD3^+^ T cells infected with GZMA-sh-rAd or RFP-sh-rAd, respectively. The cells were stained with crystal violet and quantified at 570 nm in a spectrometer. (C) The cell counting kit-8 assay. The above-stated cell was co-cultured in a 96-well plate. Cells were incubated with 100μl of a medium supplemented with 10μl of the CCK-8 solution for 1 h. The absorbance was recorded at 450nm using a microplate reader. (D) Western blots indicating the expression of activated caspase 3, F2R, and GZMA in the above-stated co-cultured cells. (E) The Calcein-AM/PI double staining assay. The above-stated cells were co-cultured in a 24-well plate. The apoptotic cells stained positively with propidium iodide (red), while the living cells were stained positively with Calcein AM (green). The PI and Calcein AM-positive cells were visualized using fluorescence microscopy. Scale bars: 80 μm. **p<0.05*, **p<0.01*, ****p<0.001*, *****p<0.0001*.

Figure 5. The molecular mechanism underlying GZMA and F2R expressions in tumor suppression. (A) Differentially Expressed Genes between high GZMA/F2R expression and low GZMA/F2R expression tissues in the LIHC from TCGA, identified using the gene set enrichment analysis (GSEA). (B) Western blots indicating the expressions of the JAK2, p-JAK2, F2R, and GZMA in the above-stated co-cultured cells. (C) Nuclear extraction assay. The nuclear protein and the cytoplasm protein in the above-stated cells were extracted using the nuclear extraction kit. Western blots indicated the level of STAT1 in the nucleus and the cytoplasm. (D) Fluorescence confocal assay results illustrating the subcellular localization of STAT1 (red) in the above-stated co-cultured cells. The nuclear DNA was stained with DAPI (blue). Scale bars: 15 μm.

Figure 6. The molecular mechanism underlying the role of F2R in GZMA expression-based tumor suppression. (A) Western blots indicating the expressions of activated caspase3, JAK2, p-JAK2, and F2R in the CD3^+^ cells co-cultured with the Huh7 cells infected with F2R-rAd, LDPRSFLL mutated motif F2R-rAd, and LDPRSFLL deleted motif F2R-rAd. (B) Western blots indicating the expressions of activated caspase3, JAK2, p-JAK2, and F2R in the CD3^+^ cells co-cultured with the Huh7 cells incubated with the LDPRSFLL motif-specific inhibitor (either SCH530348 or SCH79797). (C) The T cell-mediated tumor cell killing assay. The above-stated cells were stained with crystal violet and quantified at 570 nm in a spectrometer. (D) The Calcein-AM/PI double staining assay. The above-stated cells were co-cultured in 24-well plates. The apoptotic cells stained positively with propidium iodide (red), while the living cells stained positively with Calcein AM (green). The PI and Calcein AM-positive cells were visualized using fluorescence microscopy. Scale bars: 80 μm. (E) The nuclear extraction assay. The nuclear protein and the cytoplasmic protein in the above-stated cells were extracted using the nuclear extraction kit. Western blots indicated the level of STAT1 in the nucleus and the cytoplasm. (F) Fluorescence confocal assay results illustrating the subcellular localization of STAT1 (red) in the above-stated co-cultured cells. The nuclear DNA was stained with DAPI (blue). Scale bars: 15 μm.

Figure 7. Functional role of GZMA and F2R expressions in the antitumor efficacy of PD-1 mAb therapy. (A–B) The *C57BL/6* mice were injected with 5x10^5^ F2R-Lv-infected Hepa1-6 cells and then subjected to PD-1 mAb treatment or receiving IgG2a isotype control. (A) A schematic representation of the treatment plan for immune-competent *C57BL/6* mice. (B) Plots of Hepa1-6 tumor volumes, which were recorded once a week. (C–D) *BALB/c* nude mice, injected with GZMA-rAd or RFP-rAd-infected CD3^+^ T cells, were injected with 2x10^5^ F2R-Lv-infected Huh7 cells and then subjected to PD-1 mAb treatment or receiving the IgG2a isotype control. (C) A schematic representation of the treatment plan for immune-deficient *BALB/c* nude mice. (D) Plots of tumor volumes, which were recorded once a week. (E) Representative images of the immunohistochemistry staining of GZMA and F2R expressions in the tumor samples from HCC patients. Scale bars: 50 mm. (F) The densities of GZMA and F2R in PD-1 mAb responder and non-responder HCC patients. (G) Tumor diameters recorded by a radiologist based on CT imaging, indicated with a red line. (H) The changed tumor diameter (mm) in the HCC patients treated with PD-1 mAb. The tumors were with an increased diameter are indicated in red, while the tumors with a decreased diameter are indicated in green. (I) Spearman’s rank correlation analysis was performed to determine the quantitative correlation between the tumor diameter change and the GZMA and F2R expression levels. The results were expressed as mean ± SEM; **p<0.05*, **p<0.01*, ****p<0.001*, *****p<0.0001*.

Figure 8. Down regulation of the GZMA and F2R expressions predicted aggressive clinicopathological characteristics and poor prognosis in HCC patients. (A) The mRNA levels of GZMA and F2R in 106 pairs of tumor tissues and adjacent tissues from HCC patients, analyzed using qRT-PCR. The relative GZMA and F2R expression were normalized using GAPDH(^-ΔΔCT^). (B) IHC staining was performed for GZMA and F2R in 158 pairs of tumor tissues and adjacent tissues from HCC patients. Scale bars: 2 mm for 50x and 50 μm for 400x. The average intensity of the gray color was used to indicate the relative levels of GZMA and F2R. **p<0.05*, **p<0.01*, ****p<0.001*, *****p<0.0001*. (C) The protein levels of GZMA and F2R in 42 pairs of tumor tissues and adjacent tissues from HCC patients were semi-quantified using the western blot assay. (D) The overall and tumor-free survival assays were performed for 106 low GZMA/F2R expression and high GZMA/F2R expression HCC patients. The patients at risk are listed below.
