# Supplementary material for: Heterogeneity induced GZMA-F2R communication inefficient impairs antitumor immunotherapy of PD-1 mAb through JAK2/STAT1 signal suppression in hepatocellular carcinoma
Source: Cell Death Dis. 2022 Mar 7;13(3):213. doi: 10.1038/s41419-022-04654-7 (PMC8901912; doi:10.1038/s41419-022-04654-7)
Supplement: Supplementary file 9 — CERTIFICATE OF ENGLISH EDITING [file 41419_2022_4654_MOESM9_ESM.pdf]

尚辑 Sagesci

SCI科研服务品牌

CERTIFICATE OF ENGLISH EDITING

This is to certify that the manuscript entitled

Heterogeneity induced GZMA-F2R communication inefficient impairs antitumor immunotherapy of PD-1 mAb through JAK2/STAT1 signal suppression in hepatocellular carcinoma

By: Yuxue Gao, Qingguo Xu, Xinqiang Li, Yuan Guo, Bowen Zhang, Yan Jin, Cunle zhu, Yuntai Shen, Pengxiang Yang, Ying Shi, Rifeng Jin, Daojie Liu, Yabo Ouyang, Xiaoni Liu, Wenjing Wang, Dexi Chen, Tongwang Yang

commissioned to us has been carefully edited by a native English-speaking editor at Sagesci. The grammar, spelling, and punctuation of the text have carefully been checked and corrected wherever required. We believe that the language of this paper has been considerably improved to meet academic standards. You may please contact us for further queries regarding the editing process.

SAGESCI LANGUAGE EDITING

3301080244472

Date of issue
January 21, 2022

Disclaimer: The changes in the document may be accepted or rejected by the authors at their sole discretion after our editing. Therefore, Sagesci would not be responsible for the revisions made to this document after our editing carried on **January 21, 2022**

Sagesci
Email: china@sageeditor.cn
2000+ native English editors: www.sagesci.cn
