# Supplementary figures and images for: Heterogeneity induced GZMA-F2R communication inefficient impairs antitumor immunotherapy of PD-1 mAb through JAK2/STAT1 signal suppression in hepatocellular carcinoma
Source: Cell Death Dis. 2022 Mar 7;13(3):213. doi: 10.1038/s41419-022-04654-7 (PMC8901912; doi:10.1038/s41419-022-04654-7)

1    **Original western blots**

2    **WB in Figure 4**

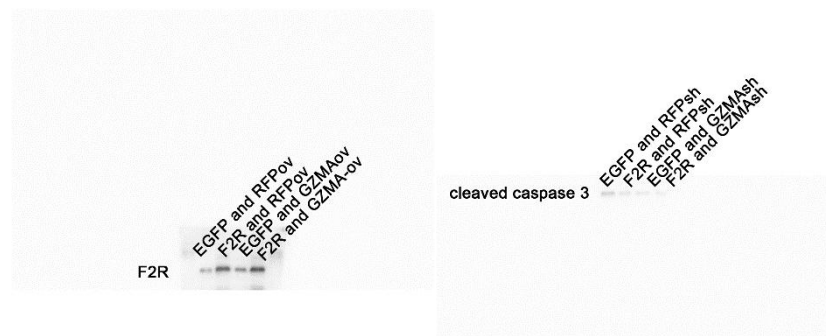

3

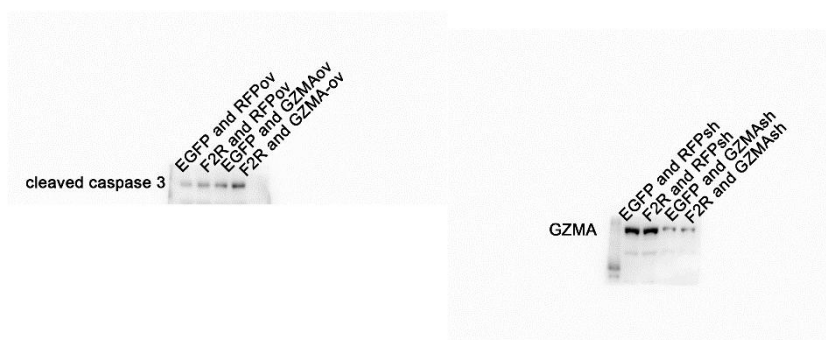

4

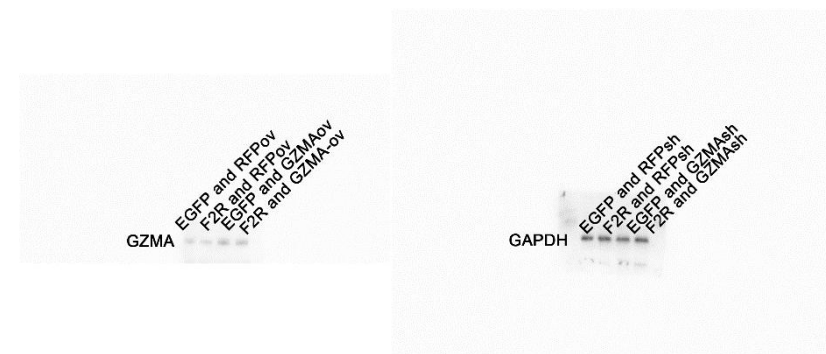

5

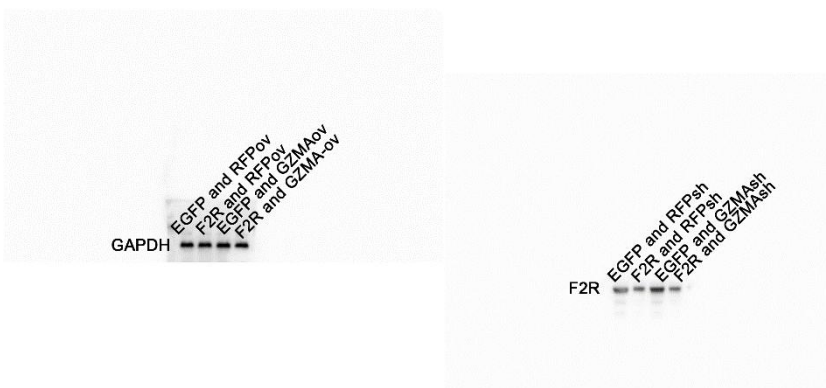

6

7    **WB in Figure 5**

8

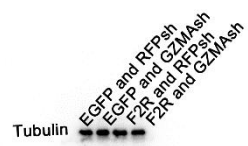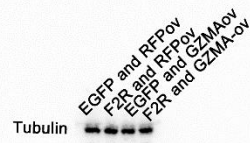

9

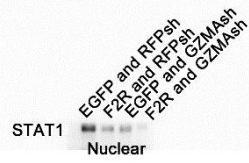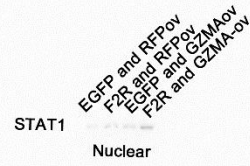

10

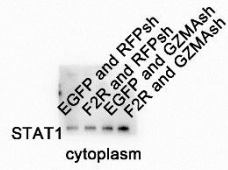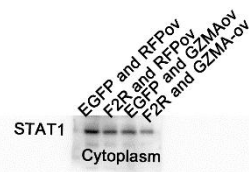

11

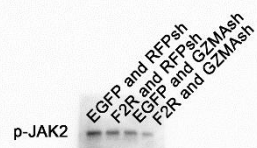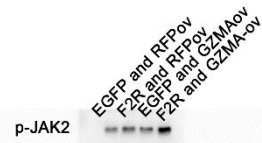

12

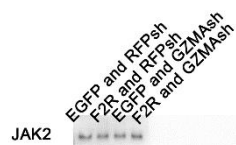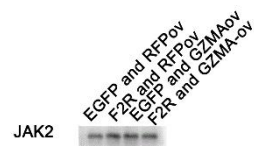

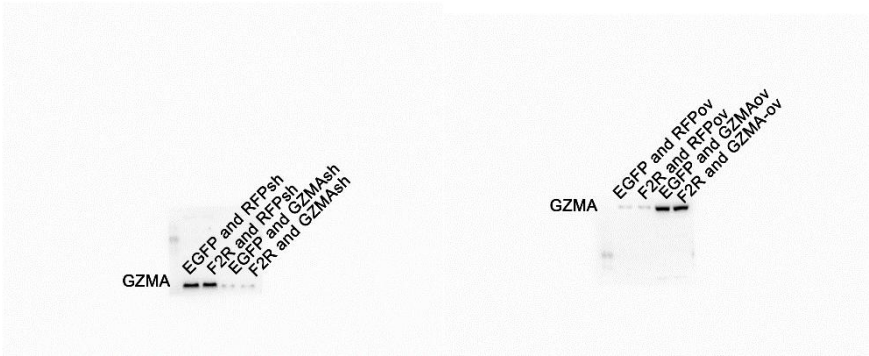

13

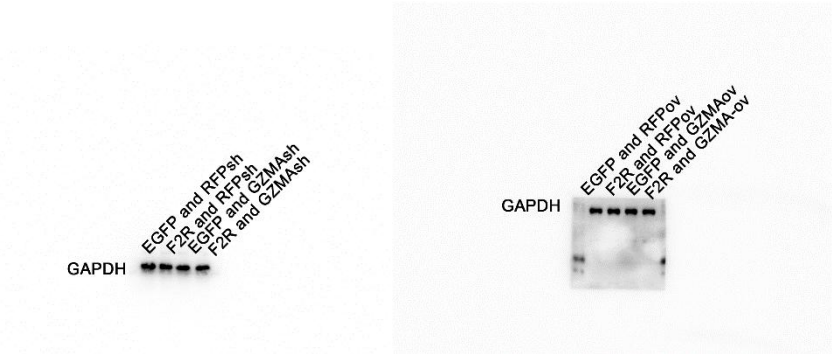

14

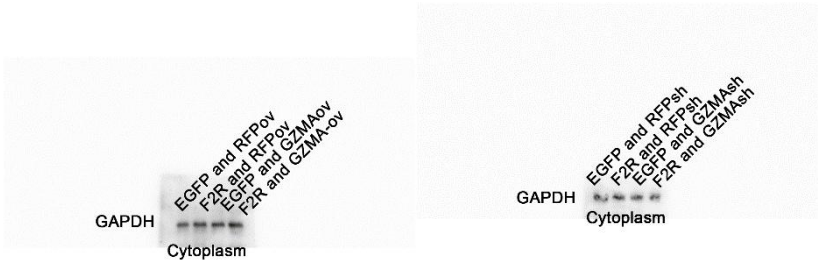

15

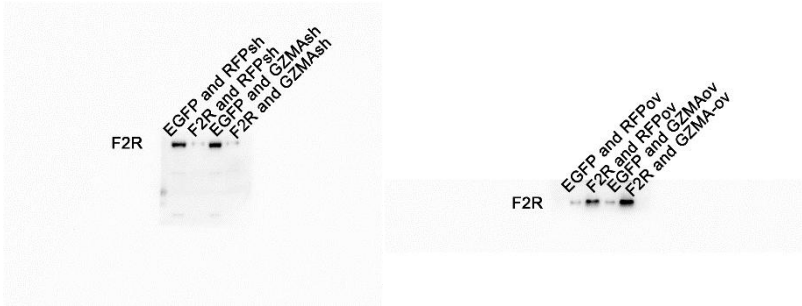

16

17 WB in Figure 6

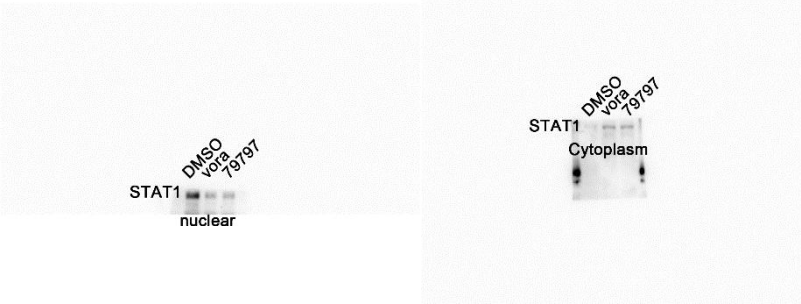

18

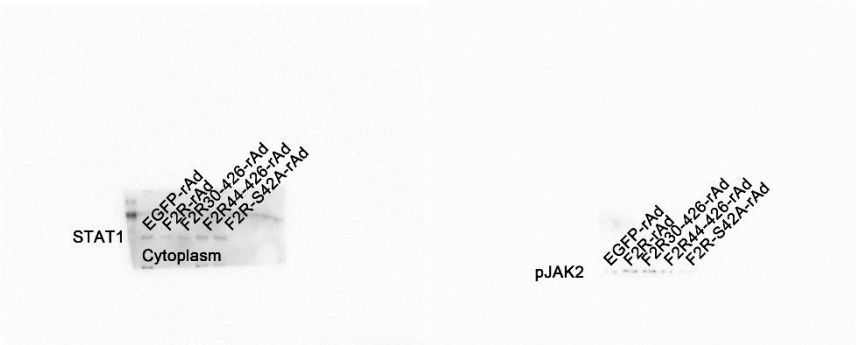

19

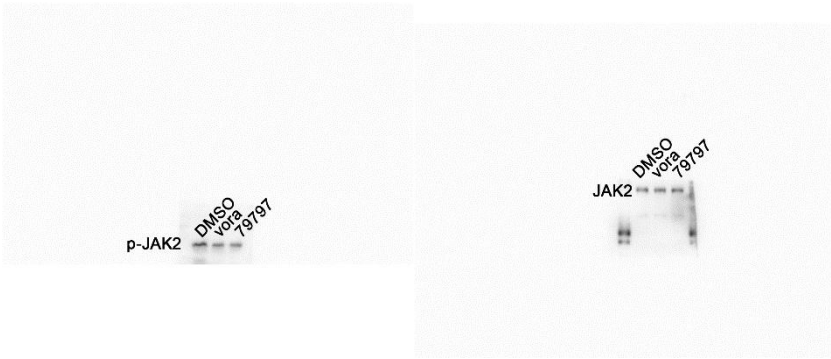

20

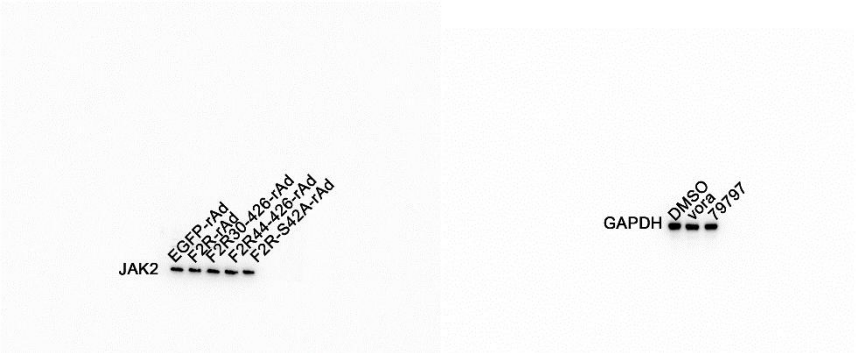

21

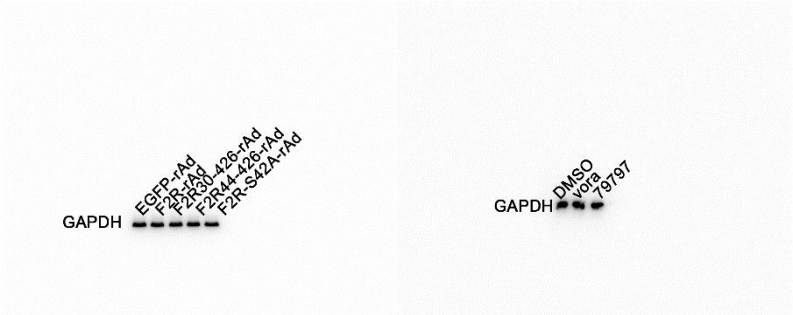

22

23

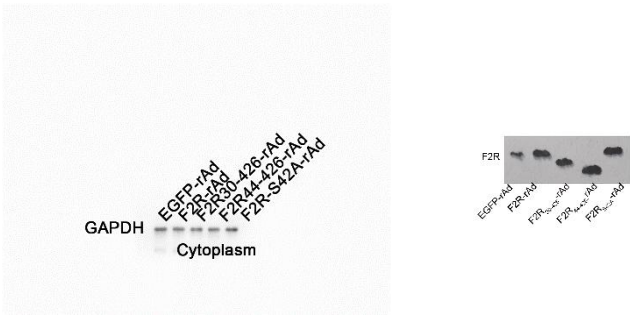

24

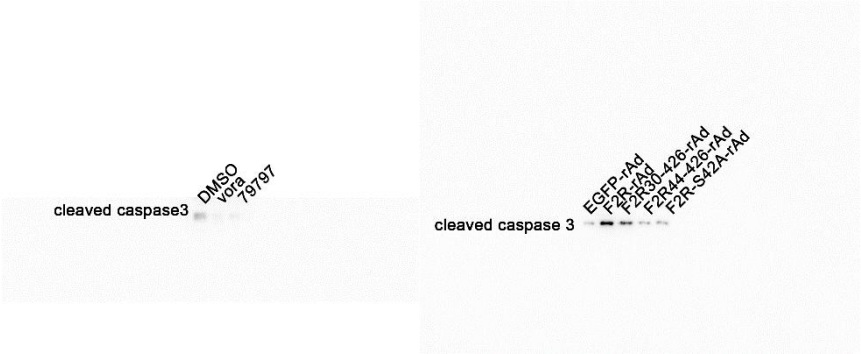

25

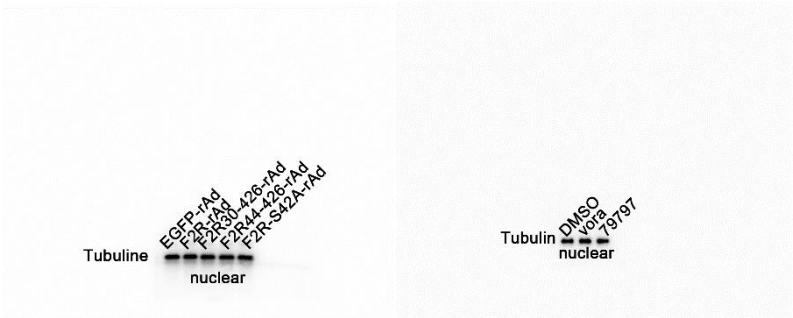

26

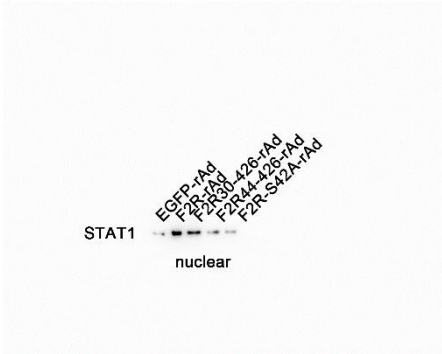

27 WB in Figure 8

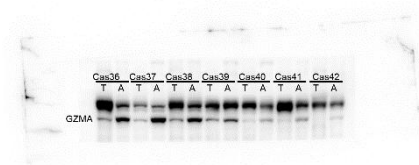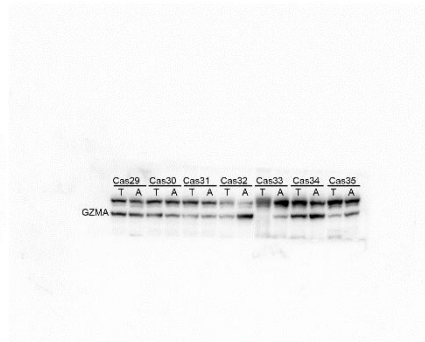

28

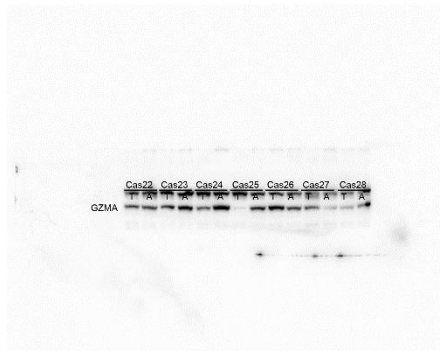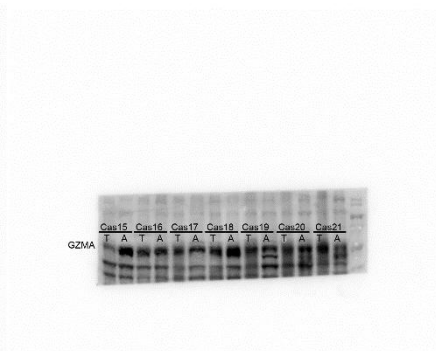

29

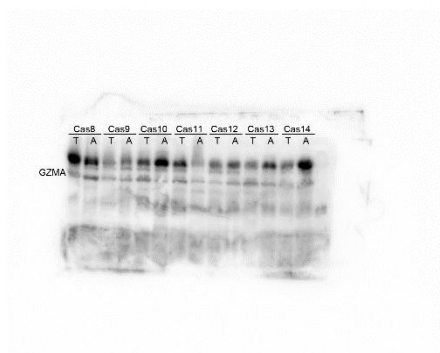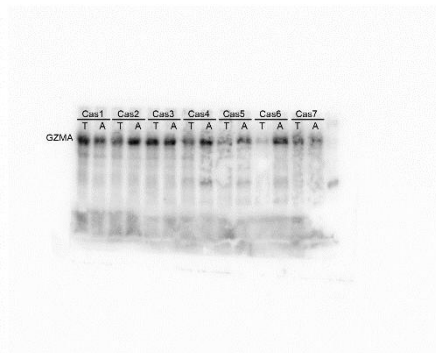

30

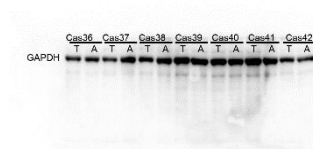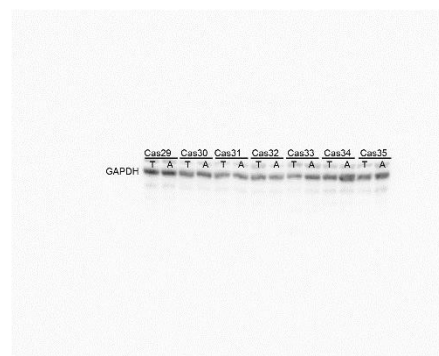

31

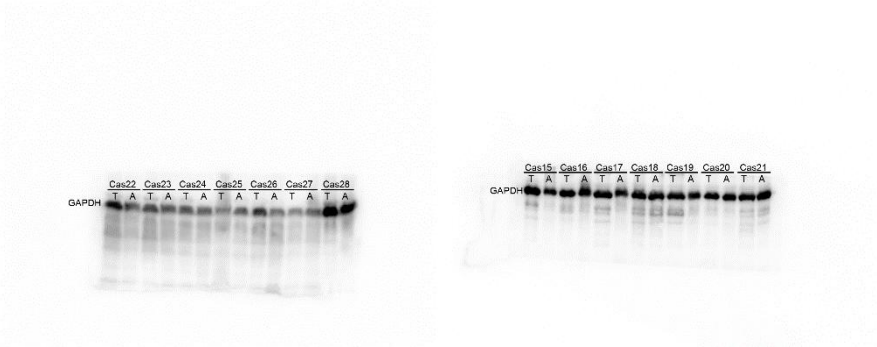

32

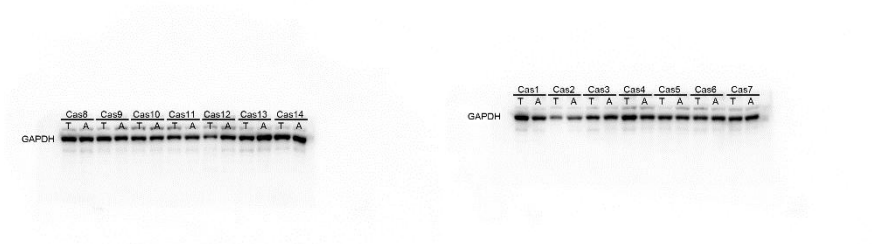

33

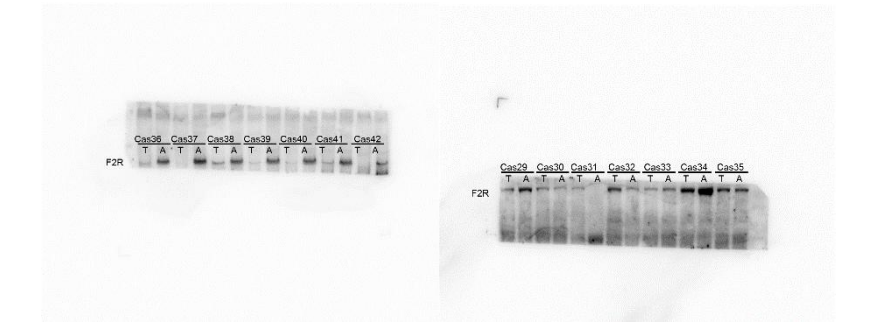

34

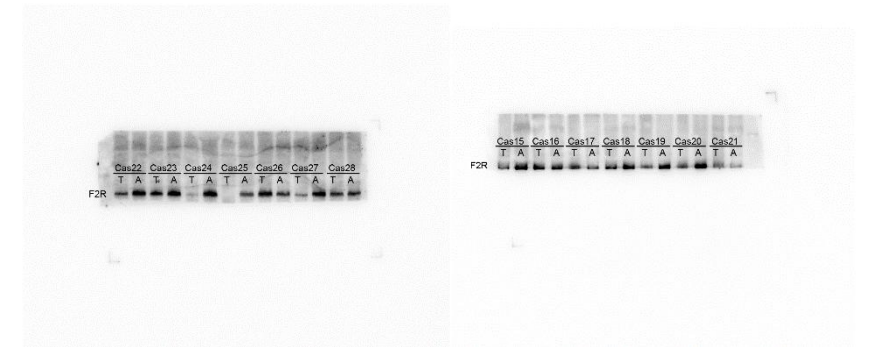

35

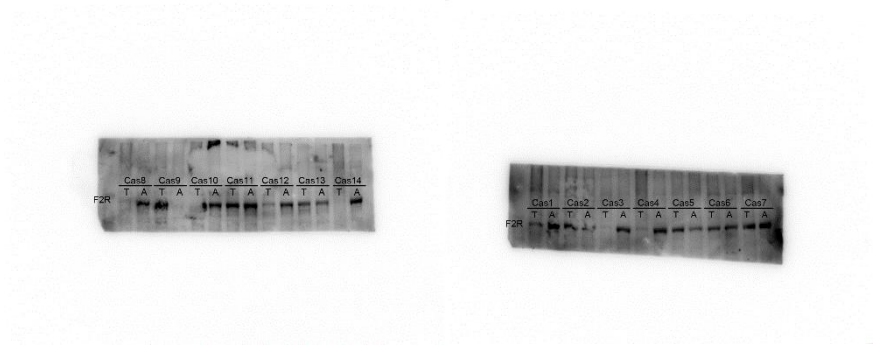

36

Supplement: Supplementary file 10 — Original western blots [file 41419_2022_4654_MOESM10_ESM.pdf]
