# Supplementary material for: Heterogeneity induced GZMA-F2R communication inefficient impairs antitumor immunotherapy of PD-1 mAb through JAK2/STAT1 signal suppression in hepatocellular carcinoma
Source: Cell Death Dis. 2022 Mar 7;13(3):213. doi: 10.1038/s41419-022-04654-7 (PMC8901912; doi:10.1038/s41419-022-04654-7)
Supplement: Supplementary file 11 — Supplementary Figures [file 41419_2022_4654_MOESM11_ESM.pdf]

Supplementary Figure legends

Figure S1. Single-cell RNA-seq analysis of the tumor and adjacent tissues of HCC patients. (A) The t-SNE map shown clusters of heterogeneous cells in the tumor and adjacent tissues from HCC patients. (B) Expression heatmap of marker genes in each cluster of heterogeneous cells (top 5, color-coded by cluster and expression). (C) PCA plot shown the source of cells from patients and tissues (tumor or adjacent). (D) The t-SNE map shown the source of cells from the tumor and adjacent tissues.

Figure S2. Molecular mechanism of cytotoxic cells exhausted in tumor tissues of HCC patients. (A) Kyoto encyclopedia of genes and genomes (KEGG) enrichment analysis of marker genes of cytotoxic cells in HCC patients. (B) Expression t-SNE maps for T cell exhausted maker genes, CD40LG and IL2RA, in tumor and adjacent tissues, respectively. (C) Biological processes gene enrichment analysis of marker genes of cytotoxic cells in HCC patients. (D) Molecular function gene enrichment analysis of marker genes of cytotoxic cells in HCC patients. (E) Cell composition gene enrichment analysis of marker genes of cytotoxic cells in HCC patients.

Figure S3. Granzyme family expression in cytotoxic cells of HCC patients. (A) Expression t-SNE maps for GZMB in cytotoxic cells from the tumor

and adjacent tissues, adjacent tissues, and tumor tissues (a-c), respectively. The bar plot shown the ratio of GZMB positive cytotoxic cells in the tumor and adjacent tissues of HCC patients (d). (B) Expression t-SNE maps for GZMM in cytotoxic cells from the tumor and adjacent tissues, adjacent tissues, and tumor tissues (a-c), respectively. The bar plot shown the ratio of GZMM positive cytotoxic cells in the tumor and adjacent tissues of HCC patients (d). (C) Expression t-SNE maps for GZMK in cytotoxic cells from the tumor and adjacent tissues, adjacent tissues, and tumor tissues (a-c), respectively. The bar plot shown the ratio of GZMK positive cytotoxic cells in the tumor and adjacent tissues of HCC patients (d). (D) Expression t-SNE maps for GZMH in cytotoxic cells from the tumor and adjacent tissues, adjacent tissues, and tumor tissues (a-c), respectively. The bar plot shown the ratio of GZMH positive cytotoxic cells in the tumor and adjacent tissues of HCC patients (d).

Figure S4. Cellular communication in cytotoxic cells and tumor cells. (A) River plot shown cell-cell communication patterns in cytotoxic cells and tumor cells. (B) The dot plot shown cell-cell communication patterns in cytotoxic cells and tumor cells. (C) Expression t-SNE map shown clusters for cytotoxic and tumor cells from the tumor and adjacent tissues of HCC patients.

Figure S5. Differentially expressed genes in GZMA/F2R-low and GZMA/F2R-high expressed LIHC patients. (A) Expression heatmap of differentially expressed genes in GZMA/F2R-low and GZMA/F2R-high expressed LIHC patients (color-coded by expression). (B) The volcano plot presented the distribution of genes expression in GZMA/F2R-low and GZMA/F2R-high expressed LIHC patients (differentially expressed gene coded by red color).

Figure S6. Identification of F2R-WT and F2R-S42A. Sanger sequencing was performed for pDC316-F2R-WT and pDC316-F2R-S42A, respectively. The base marked by the red line of the obtained peak diagram is the difference-site of two genotypes.

Figure S7. Co-expression of GZMA and F2R with PD-1 and PD-L1. (A) Co-expression of GZMA and F2R with PD-1 and PD-L1 in cancer samples from TCGA. (B) Co-expression of GZMA and F2R with PD-1 and PD-L1 in cancer samples from LICH. (C) Co-expression of GZMA and F2R with PD-1 and PD-L1 in normal samples from GTEx. Dot in the plot showing as one cancer type (A), one patient (B), or one tissue type (C).

Figure S8. Tumor suppression of GZMA and F2R in the antitumor property of PD-1 mAb therapy. (A) Subcutaneous tumor volume of Hepa1-6 cell

infected with F2R-Lv and EGFP-Lv in immune-competent *C57BL/6* mice treated with PD-1 or IgG, respectively. (B) Subcutaneous tumor volume of Huh7 cell infected with F2R-sh-Lv and EGFP-sh-Lv in immune-competent *C57BL/6* mice injected with GZMA-sh-rAd and RFP-sh-rAd infected CD3$^+$ T cells. *C57BL/6* mice were subsequently treated with PD-1 or IgG. N=6, *$p<0.05$, *$p<0.01$, ***$p<0.001$, ****$p<0.0001$.

Figure S9. Apoptosis-inducing GZMA and F2R in the antitumor property of PD-1 mAb therapy. (A) Fluorescence staining of activated caspase3 and TUNEL staining in immune-competent *C57BL/6* mice treated with PD-1 or IgG. The Hepa1-6 cells were infected with F2R-Lv or EGFP-Lv, respectively. a, representative images of fluorescence staining. b, representative images of TUNEL staining. c, quantification of activated caspase3. d, quantification of FITC positive cell. (B) Fluorescence staining of activated caspase3 and TUNEL staining in immune-deficient *BALB/c* nude mice treated with PD-1 or IgG. The Huh7 cells were infected with F2R-sh-Lv or EGFP-sh-Lv, respectively. GZMA-rAd and RFP-rAd infected CD3$^+$ T cells were injected to rebuild the immune system. a, representative images of fluorescence staining. b, representative images of TUNEL staining. c, quantification of activated caspase3. d, quantification of FITC positive cell. Scale bars, 80 mm. N=6, *$p<0.05$, *$p<0.01$, ***$p<0.001$, ****$p<0.0001$.

Figure S10. GZMA and F2R expression in cancer patients and role in the progression of HCC patients. (A) The difference of F2R and GZMA expression in tumor (green) and adjacent tissues (red) of 33 kinds of cancer patients in the TCGA database. (B) The difference of F2R and GZMA expression in tumor (orange) and adjacent tissues (green) 9 HCC GSE datasets in the GEO database. (C) The overall, disease-specific, and progression-free survival assay was performed in 187 GZMA/F2R-low and GZMA/F2R-high expressed LIHC patients. The accurate patients at risk are listed below. N=6, *$p<0.05$, *$p<0.01$, ***$p<0.001$, ****$p<0.0001$

Figure S11. Low expressed GZMA and F2R predict aggressive clinicopathological characteristics in HCC patients. (A) Relative expression levels of GZMA and F2R in HCC patients with sex, vascular infiltration, encapsulation, and thrombus. (B) Cox multivariate proportional hazard regression model was performed for multivariate analysis of the hazard ratios (HRs), and low expressed GZMA or F2R may be an independent prognostic factor for the OS and the RFS rates. *$p<0.05$, *$p<0.01$, ***$p<0.001$, ****$p<0.0001$.

Figure S12. Mechanism of GZMA-F2R communication induced JAK2/STAT1 signal activation in tumor suppression. See the discussion section for detailed information.

Figure S1

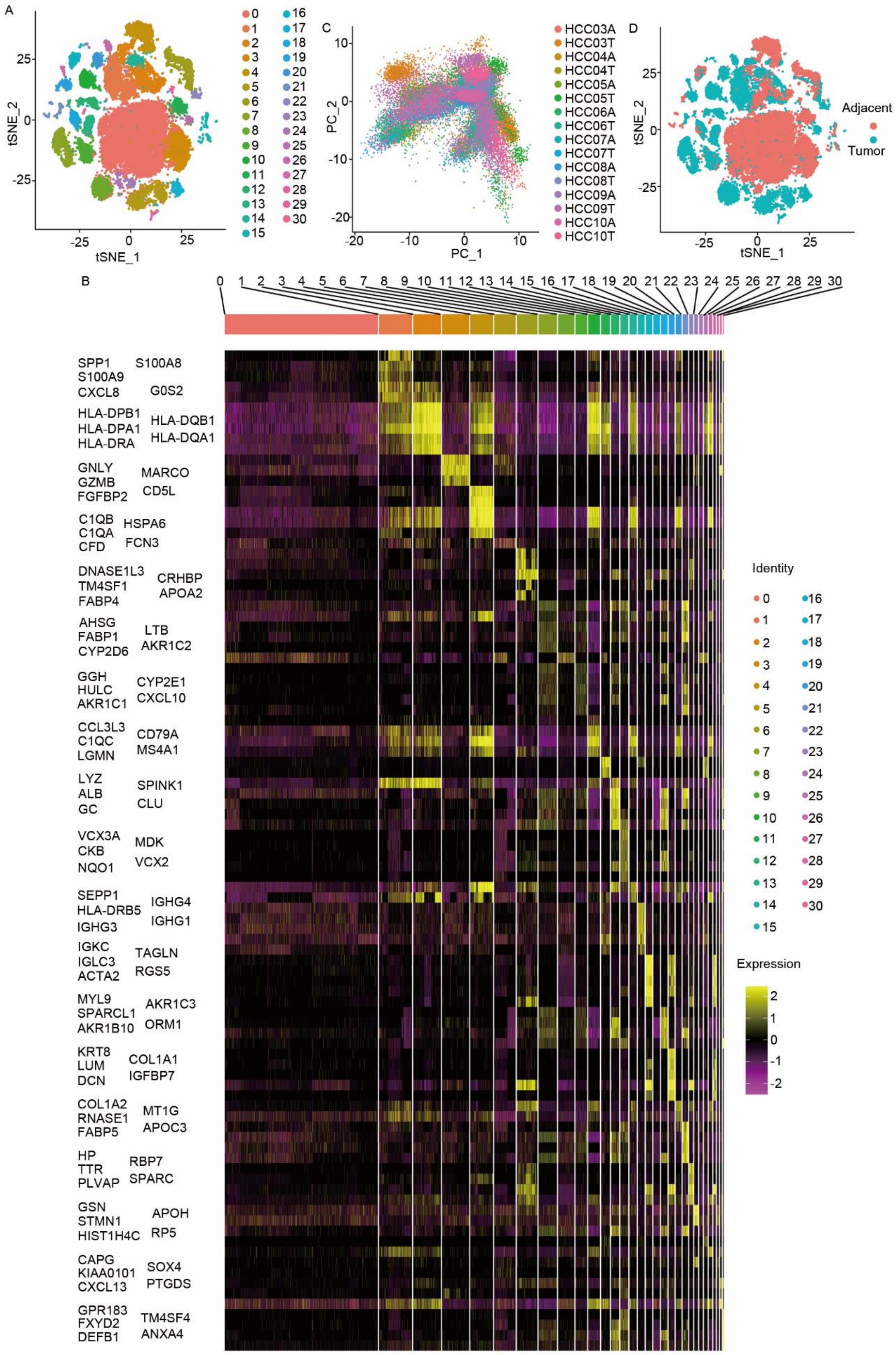
A
tSNE_1
tSNE_2
C
PC_1
PC_2
HCC03A
HCC03T
HCC04A
HCC04T
HCC05A
HCC05T
HCC06A
HCC06T
HCC07A
HCC07T
HCC08A
HCC08T
HCC09A
HCC09T
HCC10A
HCC10T
D
Adjacent
Tumor
B
SPP1 S100A8
S100A9
CXCL8 G0S2
HLA-DPB1
HLA-DPA1 HLA-DQB1
HLA-DRA HLA-DQA1
GNLY MARCO
GZMB CD5L
FGFBP2
C1QB HSPA6
C1QA
CFD FCN3
DNASE1L3
TM4SF1 CRHBP
FABP4 APOA2
AHSG LTB
FABP1 AKR1C2
CYP2D6
GGH
HULC CYP2E1
AKR1C1 CXCL10
CCL3L3
C1QC CD79A
LGMN MS4A1
LYZ SPINK1
ALB CLU
GC
VCX3A MDK
CKB VCX2
NQO1
SEPP1
HLA-DRB5 IGHG4
IGHG3 IGHG1
IGKC TAGLN
IGLC3 RGS5
ACTA2
MYL9 AKR1C3
SPARCL1 ORM1
AKR1B10
KRT8
LUM COL1A1
DCN IGFBP7
COL1A2
RNASE1 MT1G
FABP5 APOC3
HP RBP7
TTR SPARC
PLVAP
GSN
STMN1 APOH
HIST1H4C RP5
CAPG
KIAA0101 SOX4
CXCL13 PTGDS
GPR183
FXYD2 TM4SF4
DEFB1 ANXA4
Identity
Expression

Figure S2

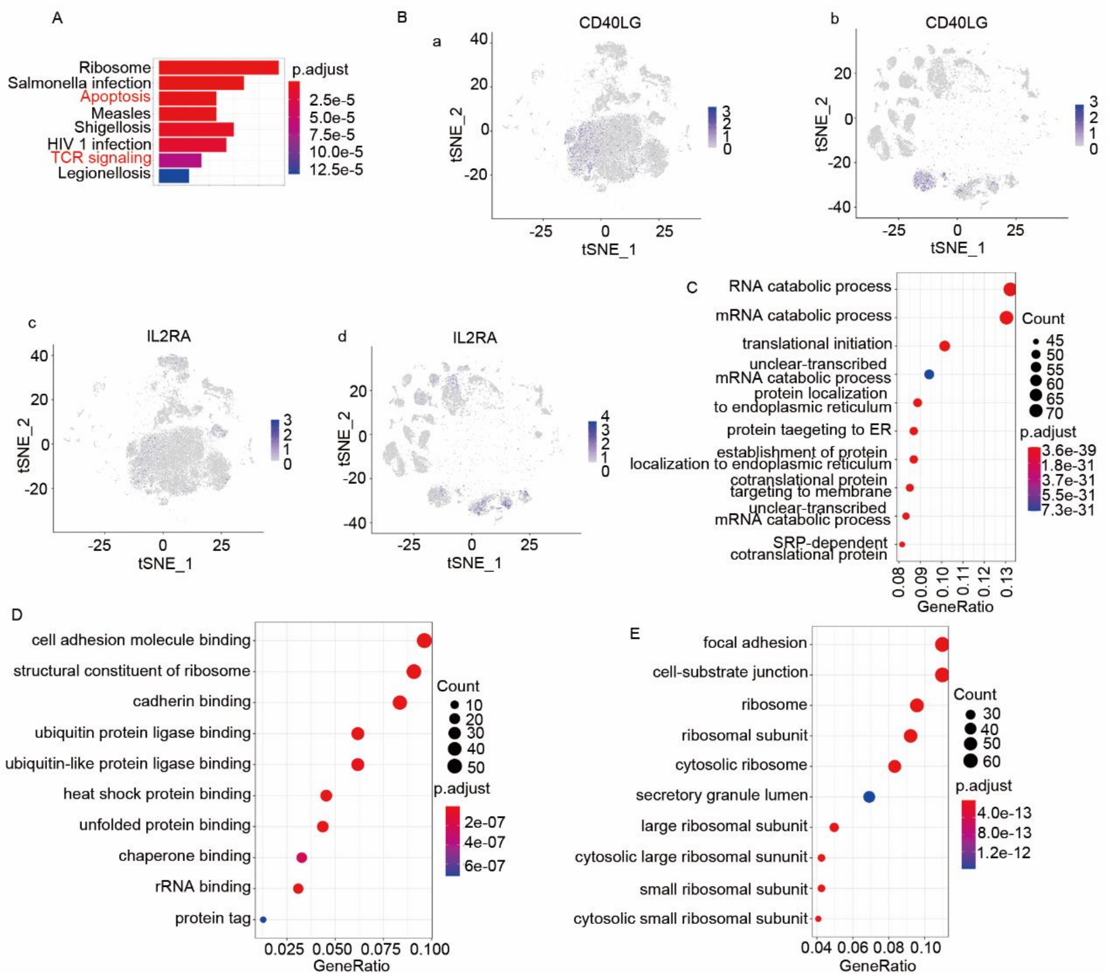
A
Ribosome
Salmonella infection
Apoptosis
Measles
Shigellosis
HIV 1 infection
TCR signaling
Legionellosis
p.adjust
B
a
CD40LG
b
CD40LG
c
IL2RA
d
IL2RA
tSNE_1
tSNE_2
C
RNA catabolic process
mRNA catabolic process
translational initiation
unclear-transcribed mRNA catabolic process
protein localization to endoplasmic reticulum
protein taegeting to ER
establishment of protein localization to endoplasmic reticulum
cotranslational protein targeting to membrane
unclear-transcribed mRNA catabolic process
SRP-dependent cotranslational protein
Count
p.adjust
GeneRatio
D
cell adhesion molecule binding
structural constituent of ribosome
cadherin binding
ubiquitin protein ligase binding
ubiquitin-like protein ligase binding
heat shock protein binding
unfolded protein binding
chaperone binding
rRNA binding
protein tag
Count
p.adjust
GeneRatio
E
focal adhesion
cell-substrate junction
ribosome
ribosomal subunit
cytosolic ribosome
secretory granule lumen
large ribosomal subunit
cytosolic large ribosomal sununit
small ribosomal subunit
cytosolic small ribosomal subunit
Count
p.adjust
GeneRatio

Figure S3

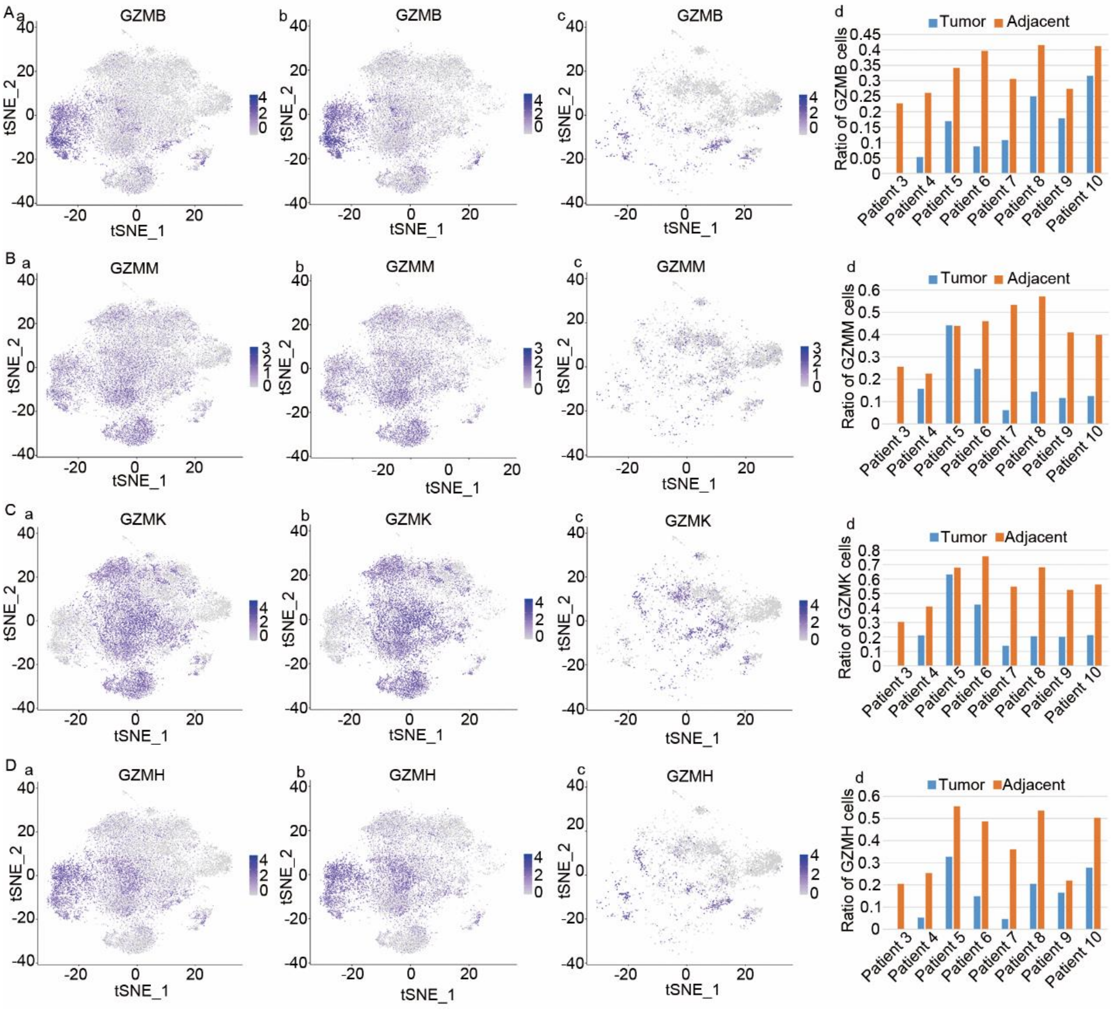

A a GZMB
b GZMB
c GZMB
d
Tumor
Adjacent
Ratio of GZMB cells
B a GZMM
b GZMM
c GZMM
Ratio of GZMM cells
C a GZMK
b GZMK
c GZMK
Ratio of GZMK cells
D a GZMH
b GZMH
c GZMH
Ratio of GZMH cells
tSNE_1
tSNE_2
Patient 3
Patient 4
Patient 5
Patient 6
Patient 7
Patient 8
Patient 9
Patient 10

Figure S4

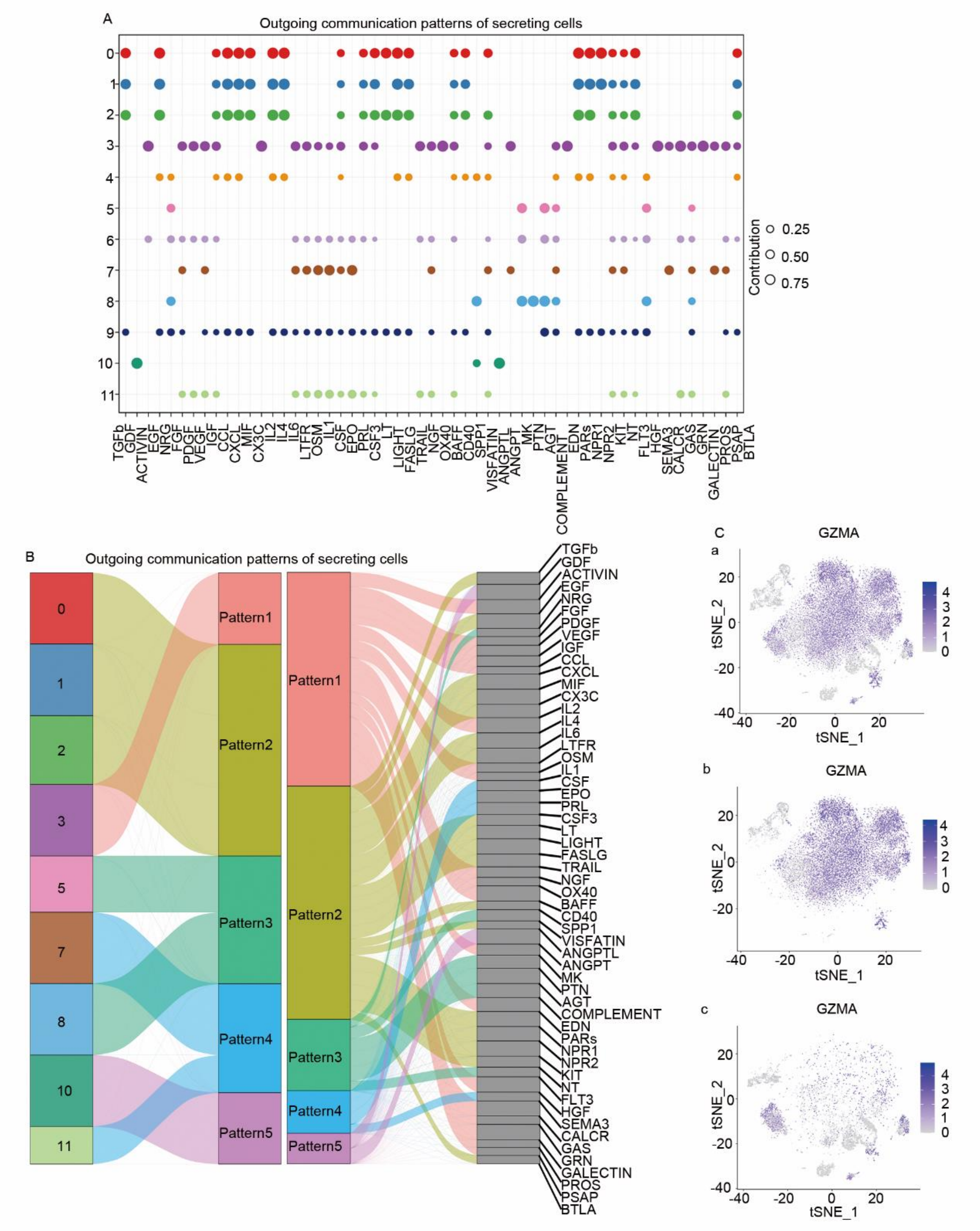
A
Outgoing communication patterns of secreting cells
Contribution
0.25
0.50
0.75
TGFb
GDF
ACTIVIN
EGF
NRG
FGF
PDGF
VEGF
IGF
CCL
CXCL
MIF
CX3C
IL2
IL4
IL6
LTFR
OSM
IL1
CSF
EPO
PRL
CSF3
LT
LIGHT
FASLG
TRAIL
NGF
OX40
BAFF
CD40
SPP1
VISFATIN
ANGPTL
ANGPT
MK
PTN
AGT
COMPLEMENT
EDN
PARs
NPR1
NPR2
KIT
NT
FLT3
HGF
SEMA3
CALCR
GAS
GRN
GALECTIN
PROS
PSAP
BTLA
B
Outgoing communication patterns of secreting cells
Pattern1
Pattern2
Pattern3
Pattern4
Pattern5
C
GZMA
tSNE_1
tSNE_2
a
b
c

Figure S5

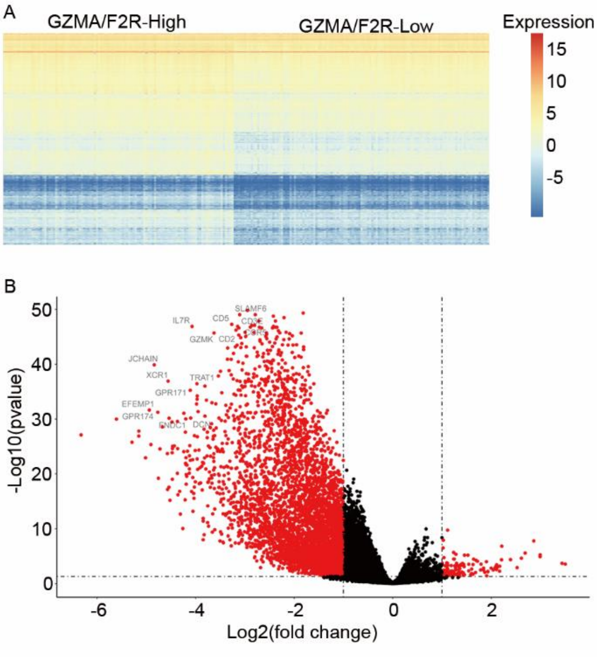
A
GZMA/F2R-High
GZMA/F2R-Low
Expression
15
10
5
0
-5
B
SLAMF6
IL7R
CD5
GZMK
CD2
JCHAIN
XCR1
TRAT1
GPR171
EFEMP1
GPR174
DCN
-Log10(pvalue)
Log2(fold change)
50
40
30
20
10
0
-6
-4
-2
0
2

Figure S6

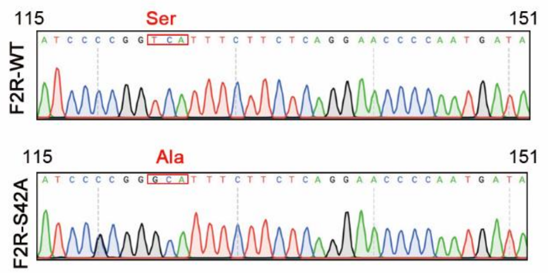
115
Ser
151
F2R-WT
A T C C C C G G T C A T T T C T T C T C A G G A A C C C C A A T G A T A
115
Ala
151
F2R-S42A
A T C C C C G G G C A T T T C T T C T C A G G A A C C C C A A T G A T A

Figure S7

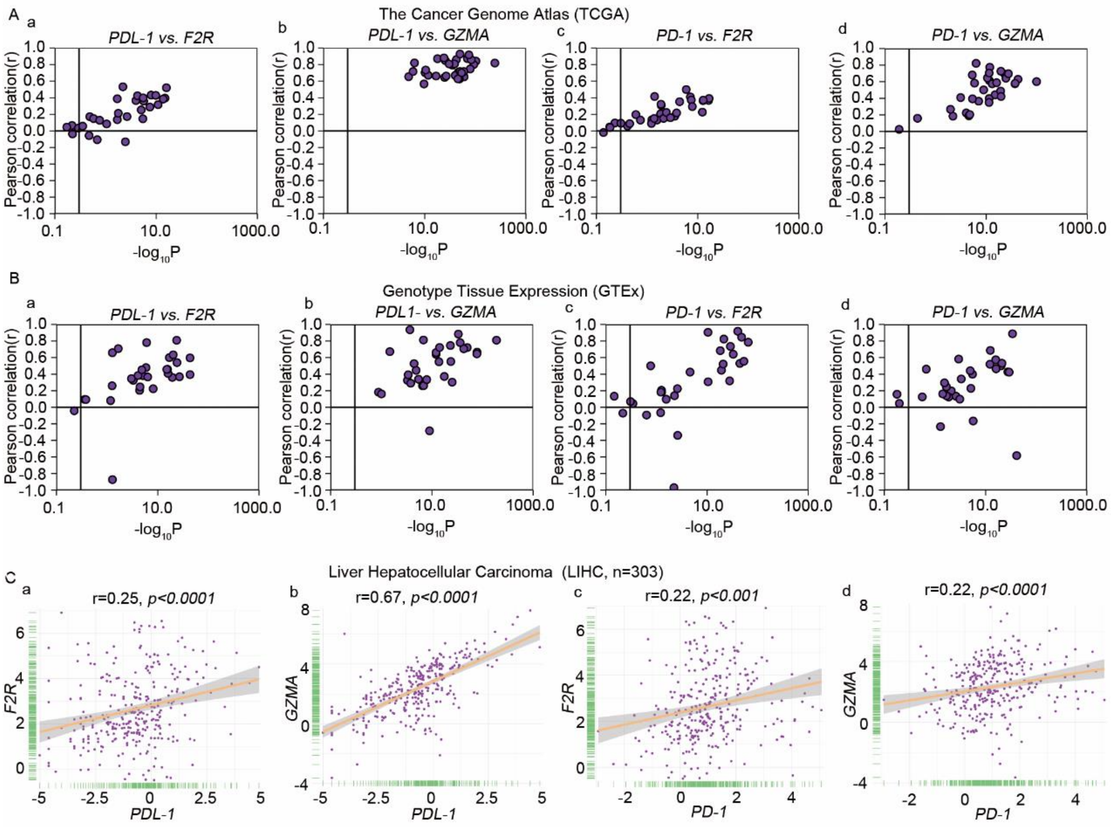

A
The Cancer Genome Atlas (TCGA)
a PDL-1 vs. F2R
b PDL-1 vs. GZMA
c PD-1 vs. F2R
d PD-1 vs. GZMA
Pearson correlation(r)
-log10P
B
Genotype Tissue Expression (GTEx)
a PDL-1 vs. F2R
b PDL1- vs. GZMA
c PD-1 vs. F2R
d PD-1 vs. GZMA
Pearson correlation(r)
-log10P
C
Liver Hepatocellular Carcinoma (LIHC, n=303)
a r=0.25, p<0.0001
b r=0.67, p<0.0001
c r=0.22, p<0.001
d r=0.22, p<0.0001
F2R
GZMA
PDL-1
PD-1

134 Figure S8

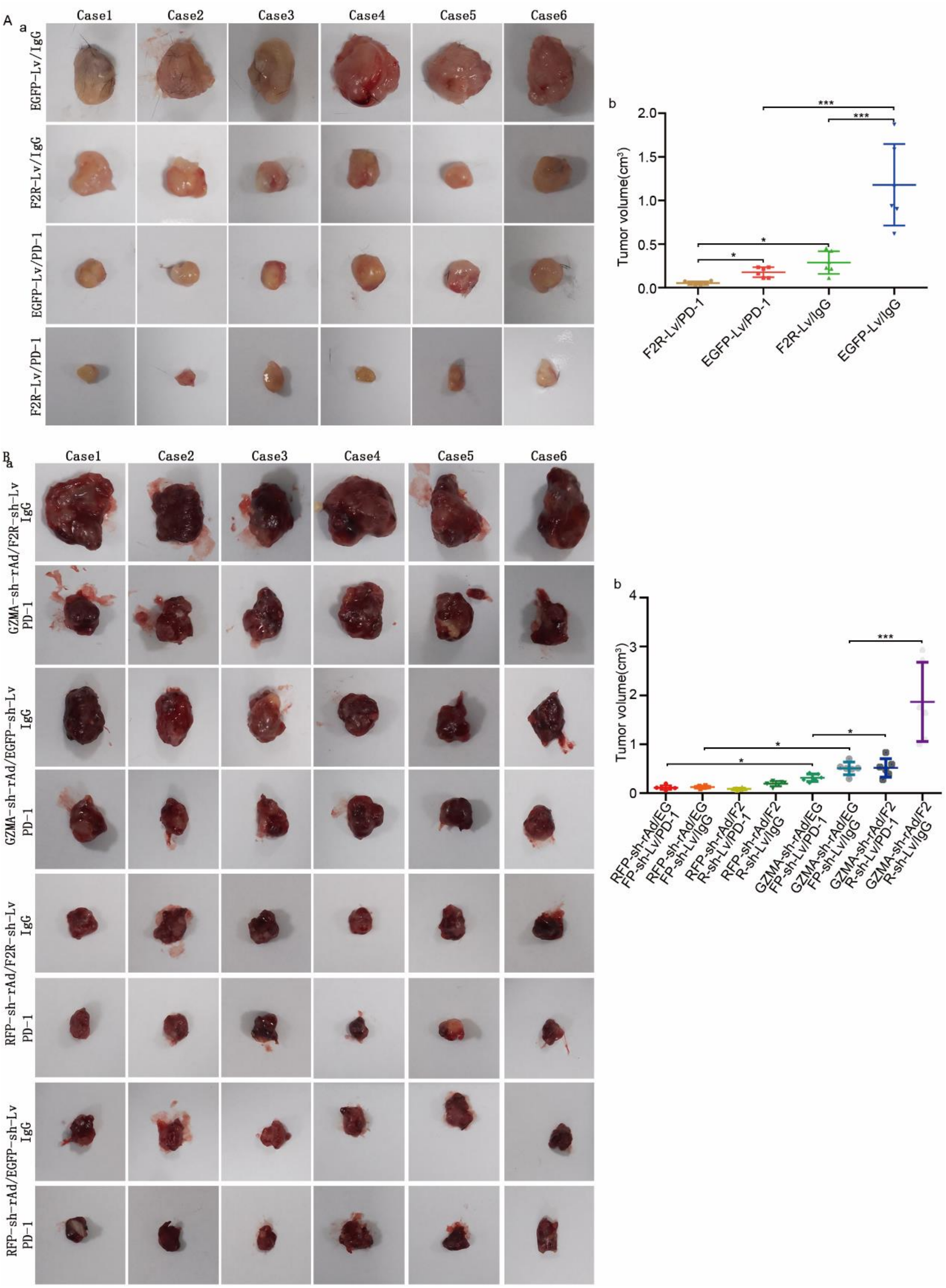

135

136

Figure S9

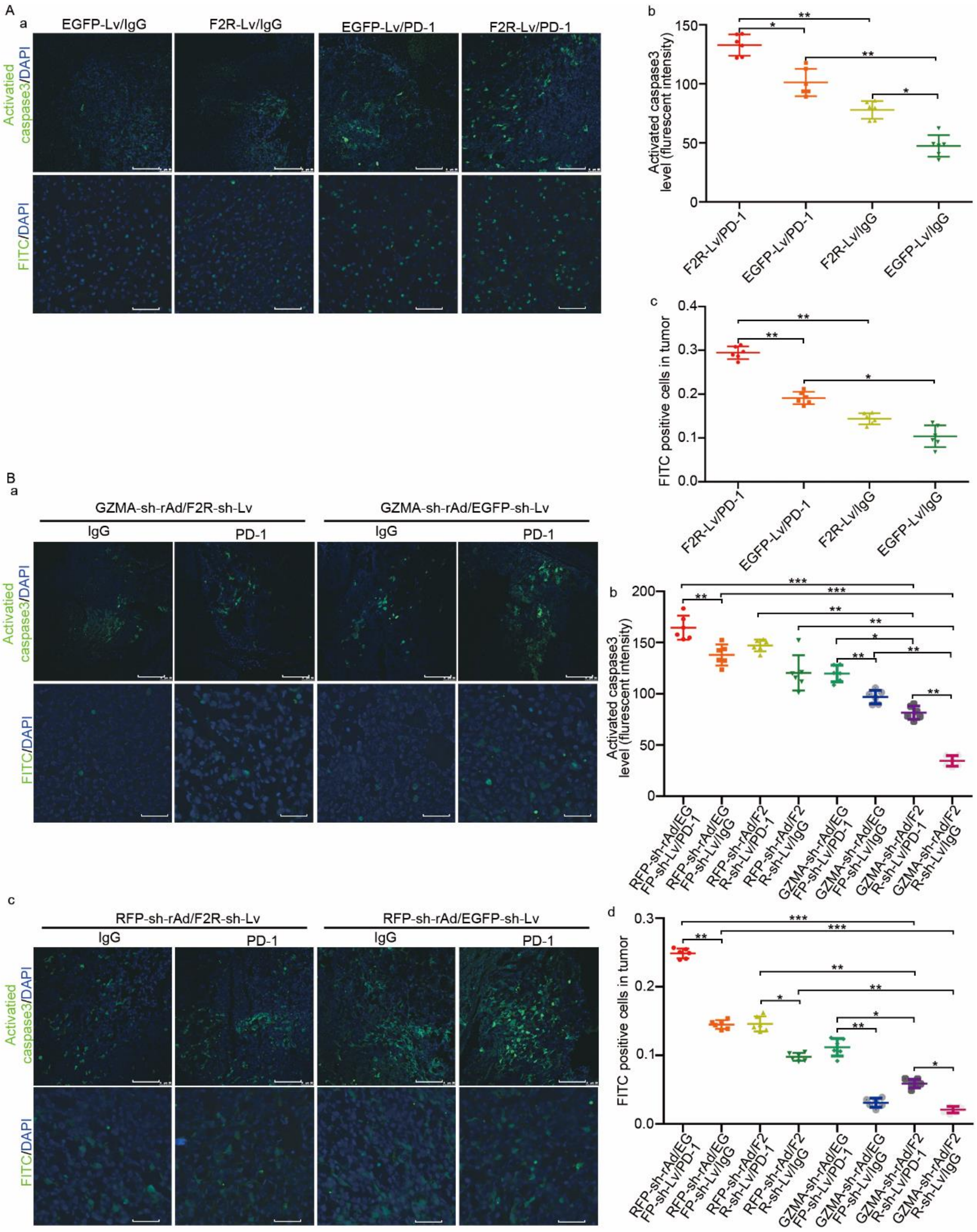
A
a
EGFP-Lv/IgG
F2R-Lv/IgG
EGFP-Lv/PD-1
F2R-Lv/PD-1
Activatied caspase3/DAPI
FITC/DAPI
b
Activated caspase3 level (flurescent intensity)
F2R-Lv/PD-1
EGFP-Lv/PD-1
F2R-Lv/IgG
EGFP-Lv/IgG
c
FITC positive cells in tumor
B
a
GZMA-sh-rAd/F2R-sh-Lv
GZMA-sh-rAd/EGFP-sh-Lv
IgG
PD-1
b
RFP-sh-rAd/EGFP-sh-Lv/PD-1
RFP-sh-rAd/EGFP-sh-Lv/IgG
RFP-sh-rAd/F2R-sh-Lv/PD-1
RFP-sh-rAd/F2R-sh-Lv/IgG
GZMA-sh-rAd/EGFP-sh-Lv/PD-1
GZMA-sh-rAd/EGFP-sh-Lv/IgG
GZMA-sh-rAd/F2R-sh-Lv/PD-1
GZMA-sh-rAd/F2R-sh-Lv/IgG
c
RFP-sh-rAd/F2R-sh-Lv
RFP-sh-rAd/EGFP-sh-Lv
d

Figure S10

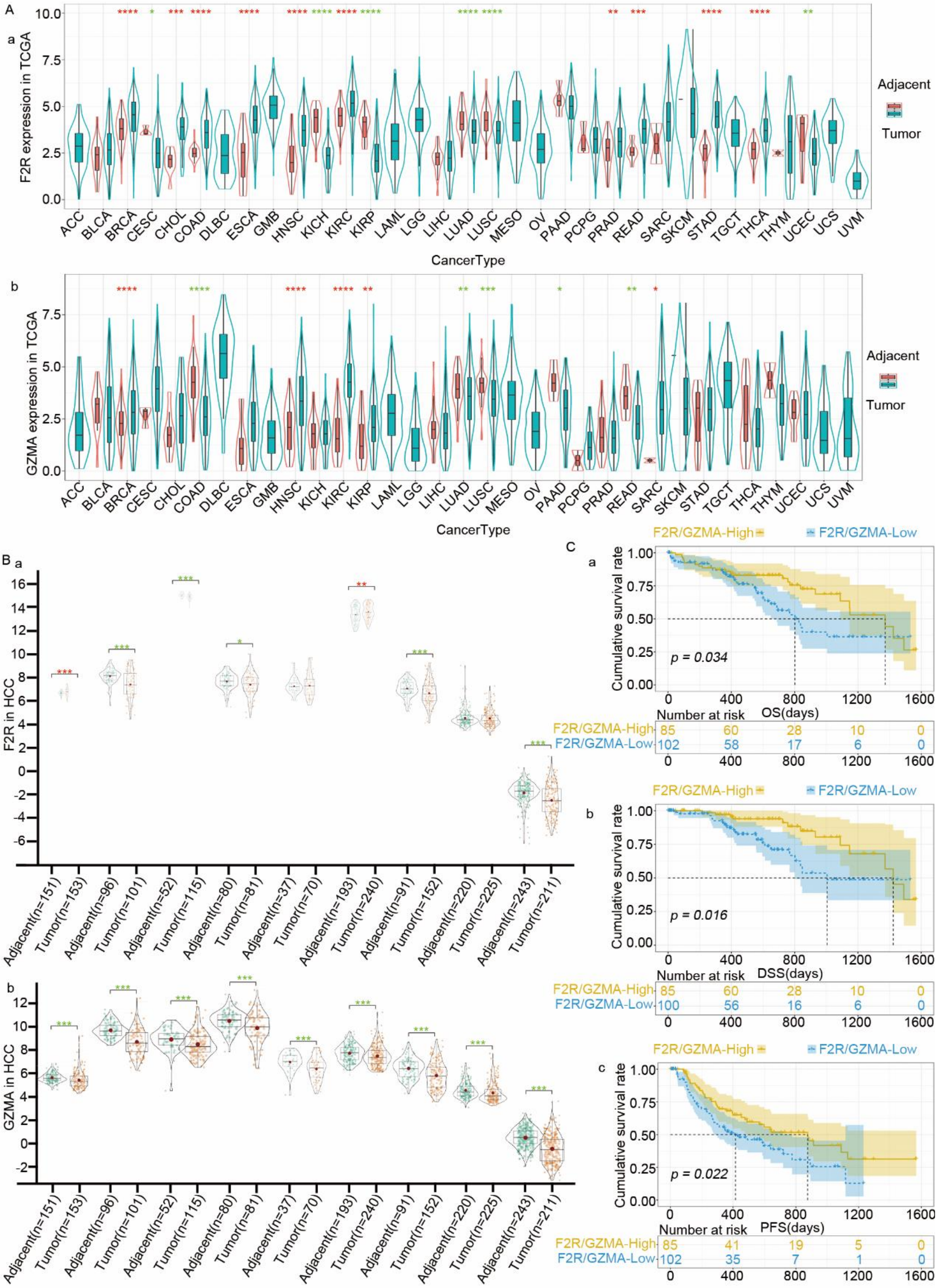

A
a
F2R expression in TCGA
Adjacent
Tumor
CancerType
ACC BLCA BRCA CESC CHOL COAD DLBC ESCA GMB HNSC KICH KIRC KIRP LAML LGG LIHC LUAD LUSC MESO OV PAAD PCPG PRAD READ SARC SKCM STAD TGCT THCA THYM UCEC UCS UVM
b
GZMA expression in TCGA
B
F2R in HCC
GZMA in HCC
Adjacent(n=151) Tumor(n=153) Adjacent(n=96) Tumor(n=101) Adjacent(n=52) Tumor(n=115) Adjacent(n=80) Tumor(n=81) Adjacent(n=37) Tumor(n=70) Adjacent(n=193) Tumor(n=240) Adjacent(n=91) Tumor(n=152) Adjacent(n=220) Tumor(n=225) Adjacent(n=243) Tumor(n=211)
C
F2R/GZMA-High
F2R/GZMA-Low
Cumulative survival rate
p = 0.034
OS(days)
Number at risk
F2R/GZMA-High 85 60 28 10 0
F2R/GZMA-Low 102 58 17 6 0
p = 0.016
DSS(days)
F2R/GZMA-High 85 60 28 10 0
F2R/GZMA-Low 100 56 16 6 0
p = 0.022
PFS(days)
F2R/GZMA-High 85 41 19 5 0
F2R/GZMA-Low 102 35 7 1 0

Figure S11

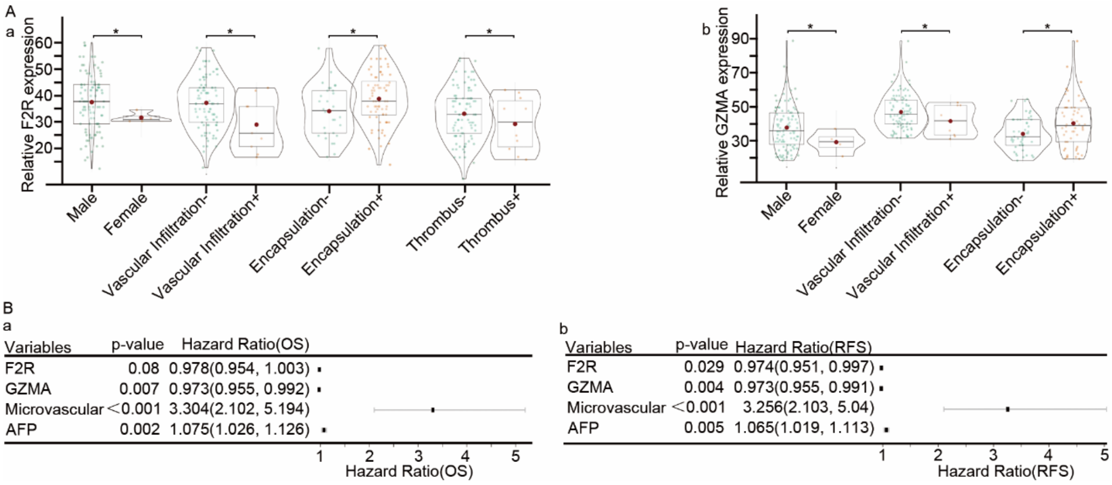
A
a
Relative F2R expression
60
50
40
30
20
*
Male
Female
Vascular Infiltration-
Vascular Infiltration+
Encapsulation-
Encapsulation+
Thrombus-
Thrombus+
b
Relative GZMA expression
90
70
50
30
B
a
Variables p-value Hazard Ratio(OS)
F2R 0.08 0.978(0.954, 1.003)
GZMA 0.007 0.973(0.955, 0.992)
Microvascular <0.001 3.304(2.102, 5.194)
AFP 0.002 1.075(1.026, 1.126)
1 2 3 4 5
Hazard Ratio(OS)
b
Variables p-value Hazard Ratio(RFS)
F2R 0.029 0.974(0.951, 0.997)
GZMA 0.004 0.973(0.955, 0.991)
Microvascular <0.001 3.256(2.103, 5.04)
AFP 0.005 1.065(1.019, 1.113)
Hazard Ratio(RFS)

Figure S12

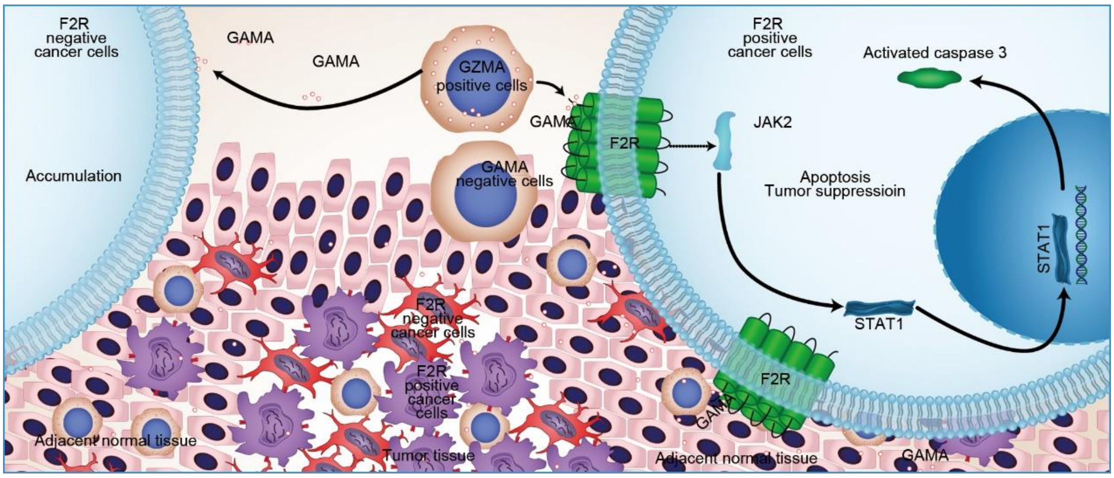
F2R negative cancer cells
GAMA
GAMA
GZMA positive cells
GAMA
F2R
GAMA negative cells
Accumulation
F2R positive cancer cells
Activated caspase 3
JAK2
Apoptosis
Tumor suppressioin
STAT1
STAT1
F2R
F2R negative cancer cells
F2R positive cancer cells
Adjacent normal tissue
Tumor tissue
Adjacent normal tissue
GAMA
